# Telomere-driven replicative crisis is driven by large-scale changes in genomic architecture

**DOI:** 10.1101/gr.281373.125

**Published:** 2026-08

**Authors:** Kate Liddiard, Emmon Coral, Harsh Bhatt, Kez Cleal, Duncan M. Baird

**Affiliations:** Division of Cancer and Genetics, School of Medicine, Cardiff University, Cardiff CF14 4XN, United Kingdom

## Abstract

Telomere-driven replicative crisis transforms the architecture of the evolving cancer genome, yet the mechanisms and consequences remain incompletely resolved, with potential biomarkers undiscovered. To address this, we have employed novel tools and methodologies to explore a human fibroblast model of crisis using high-resolution multiomic analyses. We have developed a unique chromatin conformation capture procedure for identifying distant genomic loci that interact with eroding telomeres, uncovering large-scale structural changes that accompany crisis transition. We reveal the remarkable shift from local to distant genomic interactions consistent with crisis-induced chromatin decompaction and altered gene expression. To resolve variation within challenging repetitive sequences disclosed in the complete telomere-to-telomere human reference, we have designed a targeted capture panel, uncovering aging signatures within centromeric sequences and gross copy number losses within ribosomal DNA tracks and near to the chromosome ends. We have employed both short- and long-read sequencing of purified extrachromosomal circular DNA to expose an unequivocal transition in the abundance, complexity, and sequence content of these sporadic structural variants during crisis. By integrating parallel sequencing data sets, we provide a multifaceted characterization of replicative crisis in unprecedented detail. Our findings demonstrate that telomere dysfunction and transcription-driven chromatin reorganization combine to connect replication stress to telomere fusions, eccDNA emergence and genome instability, generating dynamic biomarkers of cellular stress relevant to cancer progression.

Telomere-driven replicative crisis is characterized by the fusion of eroded and damaged telomeres that can precipitate genomic instability through breakage–fusion–bridge cycles (McClintock 1941) in the context of unchecked cellular proliferation. This volatility is fundamental to malignant transformation (Counter et al. 1992), facilitating the emergence of oncogenic genome recombinations, including those capacitating telomere length (TL) maintenance and cellular immortality (Kim et al. 1994). The crisis state can be modeled in human fibroblasts through the transgenic expression of human papillomavirus 16 (HPV16) *E6* and *E7* oncogenes that suppress endogenous RB1 and TP53 activity, concurrently attenuating cell cycle checkpoints and stimulating cell cycle progression (Shay et al. 1991; Bond et al. 1999). This results in an extended replicative life span, accompanied by aberrant genomic recombinations and inflammatory signaling (Nassour et al. 2019). Because telomere fusions are rare in cells with stable genomes (Capper et al. 2007), their detection can be associated with cancer prognosis (Lin et al. 2010; Simpson et al. 2015). Each individual telomere fusion constitutes a unique genome recombination, so the development of clinical assays will be dependent on the resolution of universal mechanisms or recurrent foci.

The critical contribution of subnuclear architecture to malignancy has been increasingly appreciated with the advent of technologies for the capture and sequence-based determination of chromatin interactions (Dekker et al. 2002; Lieberman-Aiden et al. 2009; Downes et al. 2022). Structural variants (SVs; including telomere fusions) represent both causal agents and consequences of altered chromatin architecture (Dubois et al. 2022). Mutations in chromatin loop anchors have been implicated in oncogenesis, leading to juxtaposition of ordinarily segregated loci (Khoury et al. 2020). These shifts in genomic organization both increase the probability of recombination (Sachs et al. 1997; Lin et al. 2009) between distant loci and provide opportunities for altered gene regulation through aberrant contact with enhancer or insulator sequences (Ryan et al. 2015). Enhancer hijacking (Northcott et al. 2014) is a recognized mode of oncogene activation and influence whereby inappropriate connections between regulatory sequences and gene promoters drive expression of genes that confers a survival or replicative advantage. Conversely, chromosome translocations can disrupt topologically associating domain (TAD) boundaries (Dixon et al. 2012) or the proper compartmentalization of chromatin, deranging DNA repair (Lemaître et al. 2014) and replication (Whalen et al. 2020). Drastic alterations in chromosome contacts can be observed in cancer cells, underpinning the malignant phenotype (Fritz et al. 2014; Hung et al. 2021).

Double-strand DNA breaks (DSBs) are prerequisites for SV formation as effectors of replication fork stalling and substrates of DNA repair. Gene transcription (Williamson and Lees-Miller 2011; Fong et al. 2013) and DNA replication (Xu et al. 2011; Saxena and Zou 2022) are the prevalent sources of endogenous DSBs, associating both active genes and late-replicating repetitive sequences with mutagenic processes. The coordination of these processes and natural phasing with the cell cycle also contribute to unusual and potentially deleterious long-range interactions. DSB processing results in elevated DNA mobility (Miné-Hattab and Rothstein 2012; Cho et al. 2014; Lottersberger et al. 2015), and relocalization of both coding (Penzo et al. 2023) and repetitive sequences (Khadaroo et al. 2009) to the nuclear periphery (Oza et al. 2009; Freudenreich and Su 2016) may further expedite aberrant interactions (Larizza and Colombo 2024), including telomere fusions (Liddiard et al. 2016, 2021). Persistent DNA damage contributes to replication stress that may result in the aberrant recombination of distant sites that share replication timing through a template-switching mechanism (Arlt et al. 2009; Zhang et al. 2009; Böhly et al. 2022).

Extrachromosomal circular DNA (eccDNA) represent SV that are detached from the linear genome and able to replicate independently of the cell cycle (Jaworski et al. 2025). As such, they may have serious consequences for gene copy number (CN) and transcriptional outputs, as well as subclonal heterogeneity. Because DNA circles capture regulatory as well as coding sequences and have the capacity to spatially cluster in hubs (Hung et al. 2021), their influence can extend over entire signaling networks (Hung et al. 2024). Furthermore, their detachment from chromosomal organization licenses the activation of formerly silenced loci, including transposable elements (Kraft et al. 2025) and enhancers (Mortenson et al. 2024). Large eccDNA (including double minutes) bearing oncogenic coding sequences are closely associated with genome instability and poor prognosis among cancer patients (Kim et al. 2020; Luo et al. 2023; Yan et al. 2024). Recurrent events characterize particular cancers, such as the amplification of *EGFR* in glioma (Sauter et al. 1996) and *ERBB2* in breast cancer (Vicario et al. 2015), offering opportunities for novel diagnostics (Hung et al. 2022; Behrouzi et al. 2025) and therapeutics (Von Hoff et al. 1992; Shimizu et al. 2007).

Although the oncogenic impact of large eccDNA is well recognized (Peng et al. 2022; Luo et al. 2023), there exist multiple different species of varying sizes and stability, generated by diverse cellular processes (Yang et al. 2022) that overlap with those implicated in SV formation (Zhou et al. 2024). The detection of eccDNA in healthy (Møller et al. 2016, 2018) as well as cancerous cells (Kumar et al. 2020; Tatman and Black 2022) affirms the formation of at least subsets of these events as byproducts of constitutive processes including transcription, replication, DNA repair, and apoptosis. A classification system based on size, DNA content, and pathogenic effects has been proposed (Zhou et al. 2024), yet functional stratification remains constrained by limited studies of eccDNA heterogeneity in cancer. In particular, the intracellular localization (Kanda et al. 2001; Schenkel et al. 2023), degradation (Qiu et al. 2021), immunogenicity (Wu et al. 2024b), and capacity for reintegration into the linear genome (Alitalo et al. 1983; Vogt et al. 2014) of these different eccDNA species are incompletely understood with respect to cellular function and transformation. However, accumulation in aging tissue in accordance with replication stress, genome instability, and inflammation recommends eccDNA as biomarkers of disease even when recurrent or high CN events cannot be identified.

In this study, we sought to disentangle the relative contributions of telomere attrition, replication stress, genome instability, and SV formation to the evolution of the cancer genome and phenotype. Because these processes are typically investigated in isolation, their interdependencies and shared genomic contexts remain poorly defined. We therefore designed a multimodal sequencing strategy to enable integrated analyses across genomic features, spatial contexts, and variant classes with the aim of identifying common patterns and mechanisms that could inform the rational development of future diagnostic assays and therapeutic strategies.

## Results

### Multiomic profiling of telomere-driven replicative crisis in human fibroblasts

**Figure 1.**
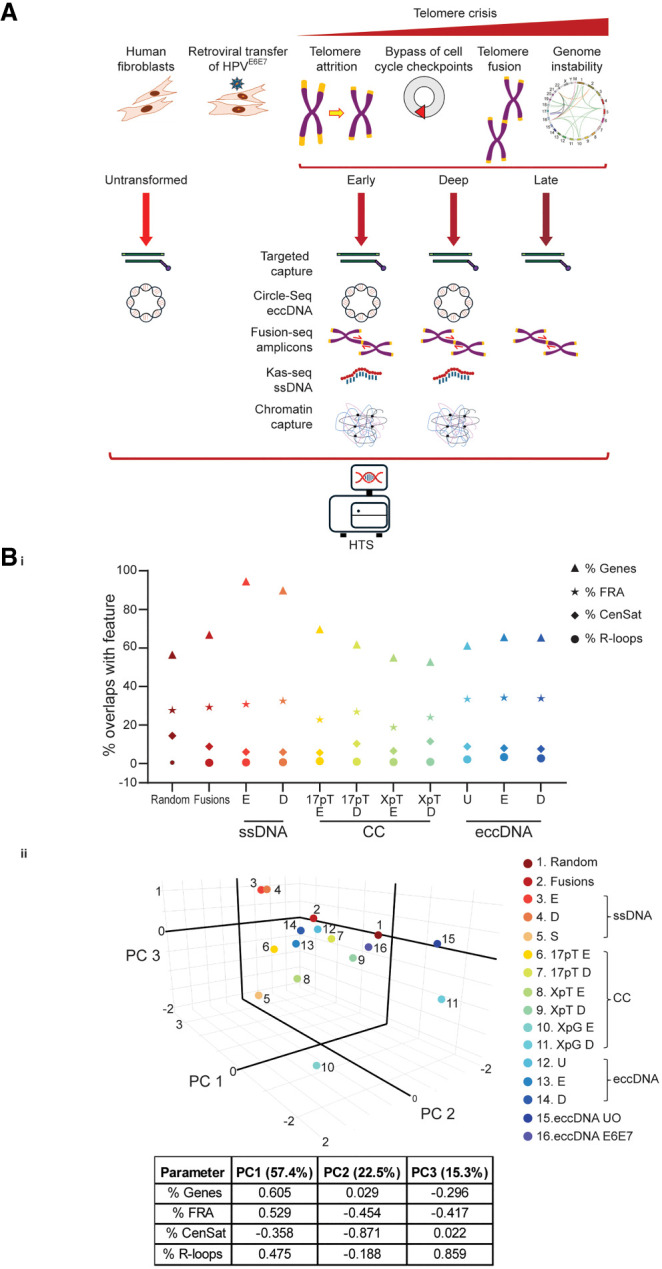
Multiomic profiling of telomere-driven replicative crisis in human fibroblasts. (*A*) A schematic of the human MRC5 fibroblast model of telomere-driven replicative crisis and the high-throughput sequencing (HTS) data sets analyzed in this study. Untransformed and MRC5 transformed by retroviral transfer of human papillomavirus 16 (HPV) *E6* and *E7 (*E6E7) oncogenes (Bond et al. 1999) were sampled for targeted capture sequencing, Circle-Seq (Møller 2020), Fusion-seq (Liddiard et al. 2016), Kas-seq (Lyu et al. 2022), and chromatin capture (Downes et al. 2022) sequencing as indicated. Transformed MRC5 (MRC5^E6E7^) were cultured through replicative crisis toward growth arrest with DNA extracted at Early (23 population doublings [PD]), Deep (PD47), and Late (PD57) time points after the appearance of telomere fusions. (*B*,*i*) The proportions of genomic intervals derived from key data sets analyzed in this study that overlap with genomic features of interest. (Genes) Coding sequence, (FRA) fragile sites, (CenSat) centromere satellites, and (R-loops) three-stranded nucleic acid structures. Samples include a simulated data set of 1 million random genomic loci (random), telomere fusions sequenced from MRC5^E6E7^ crisis cells (fusions), single-strand DNA (ssDNA) peaks determined by KAS-seq in Early (E) or Deep (D) crisis samples, chromatin capture (CC) data sets collected at Early (E) and Deep (D) time points using hybridization probes targeting the Chr17p (17pT) and ChrXpYp (XpT) telomere-adjacent sequence, and extrachromosomal circular DNA (eccDNA) from Untransformed (U) and MRC5^E6E7^ cells at Early (E) and Deep (D) crisis stages. (*ii*) A principal component analysis (PCA) plot (Galaxy Community 2024) of an extended range of data sets reveals the clustering of these samples based on the variance of these core parameters (indicated in the table *below*). Samples are encoded as in *i*, with the inclusion of ssDNA peaks common to both Early- and Deep-crisis samples (Shared [S]), CC data sets from Early (E) and Deep (D) crisis stages generated using the noncoding ChrXp genomic locus hybridization probe (XpG), and eccDNA data sets exclusive to Untransformed MRC5 (UO) or crisis MRC5^E6E7^ (E6E7) cells.

We previously reported a correspondence between genes differentially expressed and recombined with telomeres during replicative crisis in human fibroblasts (Liddiard et al. 2021). To uncover the mechanisms by which eroded telomeres become juxtaposed with transcribed genes and the consequences of altered long-range chromosomal interactions, we performed multimodal sequencing of HPV16 *E6E7* transformed MRC5 cells (Fig. 1A) transiting replicative crisis (Supplemental Fig. S1A). Because replicative crisis is triggered by telomere dysfunction (Capper et al. 2007), we defined “Early,” “Deep,” and “Late” crisis time points according to the emergence of telomere fusions in our MRC5^E6E7^ model (Supplemental Fig. S1B). In most experiments, an Early (23 population doublings [PD]) MRC5^E6E7^ sample was compared with a Deep (PD47) crisis-stage sample (Supplemental Fig. S1A) that exhibited a greater abundance of telomere fusions at multiple chromosome ends (Supplemental Fig. S1B), associated with shortened TL (Supplemental Fig. S1C). These sampling points were selected to elucidate DNA structural and organizational transitions independent of cellular proliferative capacity, volume, and viability (Supplemental Fig. S1D), which are compromised in Late crisis and senescence (Mitsui and Schneider 1976; Neurohr et al. 2019; Liddiard et al. 2021). For eccDNA (Circle-Seq) sequencing (Fig. 1A), baseline genomic information was critical to the interpretation of crisis-mediated effects and was provided by sampling of “Untransformed” MRC5. For targeted capture genome instability assays, a terminal Late (PD57) crisis MRC5^E6E7^ sample was also included for more comprehensive longitudinal comparisons.

Chromatin capture (Capture-C) (Downes et al. 2022, 2023) was performed using probes specific for Chr17p (17pT) and ChrXpYp (XpT) telomere-adjacent sequences, as well as an intergenic internal ChrXpYp (XpG) control locus (Supplemental Fig. S2) to characterize the diversification of telomere contacts in crisis. Circle-Seq (Supplemental Fig. S3; Møller 2020) was employed to distinguish eccDNA produced in “normal” compared with transformed cells and determine interrelationships with telomere fusions and gross karyotypic changes. KAS-seq (Lyu et al. 2022) was used to colocalize sites of DNA replication, transcription, and repair with SVs identified through parallel sequencing processes. Targeted capture experiments were conducted across the whole range of MRC5 samples to explore potentially unstable sequences under the pressure of crisis and their associations with telomere dysfunction.

These related data sets were analyzed for recurrent locations and features informative of the origins and effectors of genomic variation (Fig. 1Bi). Sequence context proved a potent classifier, resulting in clear sample segregation by crisis stage and underlying vulnerabilities (Fig. 1Bii; Supplemental Fig. S4). Specifically, telomere fusions clustered with eccDNA and Late-crisis Capture-C data sets, suggesting common mechanisms driving genomic rearrangements.

### Single-strand DNA peaks outline transcriptional networks and crisis-induced replication stress

**Figure 2.**
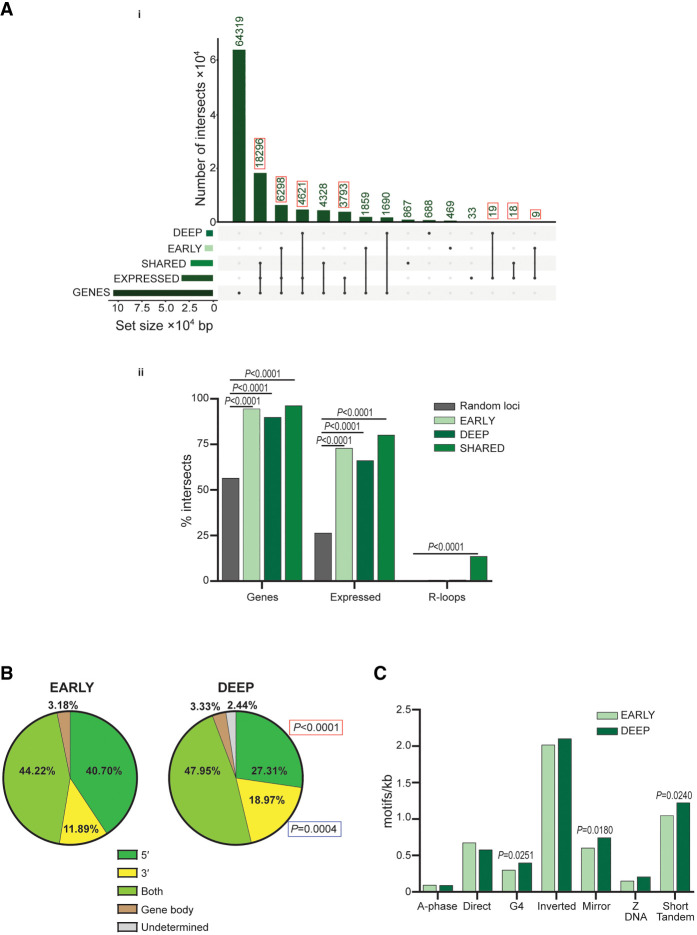
Correspondence of single-strand DNA (ssDNA) peaks with transcription and telomere-interacting sites in replicative crisis. (*A*, *i*) UpSet plot (Lex et al. 2014) depicting the overlaps (intersections) between MRC5^E6E7^ KAS-seq data sets and coding sequence (Genes) or genes expressed by MRC5^E6E7^ during crisis (Expressed) (Liddiard et al. 2021). (Early, Deep) Crisis stages, (Shared) ssDNA peaks common to both stages. Connected dots indicate the data sets included in each intersection, with vertical bars showing the number of elements in that intersection, enumerated *above* the bars. Key intersections are boxed in red. The data set (Set) sizes in nucleotide base pairs are depicted as horizontal bars. (*ii*) The proportions of genomic intervals associated with the specified Kas-seq ssDNA data sets or 1 million random genomic loci that intersect with genomic features are displayed. (Early, Deep) Crisis stages, (Shared) ssDNA peaks common to both stages, (Genes) coding sequence, (Expressed) genes expressed by MRC5^E6E7^ during crisis (Liddiard et al. 2021), and (R-loops) three-stranded nucleic acid structures. Differences were evaluated using the N − 1 χ^2^ method (*P*-values indicated). (*B*) Pie charts representing the fractions of Early- and Deep-crisis genes with ssDNA enriched at 5′ or 3′ locations or detected at both 5′ and 3′ ends or throughout the entire gene body. (Undetermined) Negligible ssDNA signal. The results of N − 1 χ^2^ comparisons of the 5′ and 3′ ssDNA gene enrichments are denoted next to the Deep-crisis chart. (*C*) The 5 kb genomic sequence extending from the 3′ termini of genes with prevailing 3′ ssDNA signal was assessed for presence of putative non-B DNA repeat motifs (*x*-axis; defined by Advanced Biomedical Computational Science [ABCS]) (Cer et al. 2012). Incidence rates (number of motifs per kilobase of input DNA) were compared for Early- and Deep-crisis samples, and the *P*-value for the incidence rate ratio is indicated. (G4) G-quadruplex repeats.

Investigating the impact of DNA damage (KAS-seq) for chromatin remodeling, we realized a correspondence between single-strand DNA (ssDNA) signal peaks and coding sequence (Fig. 2Ai) irrespective of crisis stage. Using our crisis-cell RNA-seq data (Liddiard et al. 2021), we were able to corroborate the active expression of these genes within MRC5^E6E7^ cells. Compared with a data set derived from 1 million random genomic coordinates (generated using BEDTools RandomBED) (Quinlan and Hall 2010; Galaxy Community 2024), ssDNA peaks called from discrete crisis samples exhibited substantially higher proportions of genomic intervals overlapping with genes and expressed genes (Fig. 2Aii). Only the ssDNA peaks detected throughout crisis (“Shared” between data sets) displayed significant association with R-loops (Supplemental Fig. S4), suggesting that, although unique ssDNA peaks demarcate stage-specific transcription, common peaks likely represent sites of replication stress or transcription–replication clashes. Whereas the Early-crisis ssDNA peaks data set (2455 genes) was enriched for genes involved in biogenesis and proliferation (Supplemental Fig. S5A), the Deep-crisis data set (2001 genes) showed the inflammation that accompanies persistent DNA damage and cellular stress (Nassour et al. 2019; Lex et al. 2020; Dust et al. 2022; Gulen et al. 2023). Genes bearing ssDNA marks at both crisis stages (Shared; 1668 genes) were typically longer genes (Supplemental Fig. S5B) more predisposed to replication errors (Wei et al. 2016; Berkemeier et al. 2025). Global transcription declined with crisis transition (Supplemental Fig. S5C), but this was insignificant for genes closer to the telomeres with lower mean lengths (Supplemental Fig. S5D). Consonantly, ssDNA peaks in Deep crisis marked shorter genes (Supplemental Fig. S5B) closer to the chromosome termini (Supplemental Fig. S5E), affirming telomere-proximal gene expression accompanying telomere attrition (Baur et al. 2001).

Ascribing KAS-seq signal peaks to 5′ or 3′ gene locations revealed a compelling reduction in ssDNA peaks overlapping transcription start sites (TSSs) in Deep crisis (Fig. 2B) and corresponding elevation in 3′ DNA damage characteristic of replication fork stalling and structural instability (Li and Manley 2005; Gadgil et al. 2020, 2024; Stoy et al. 2023). We observed increased incidence of G4-quadruplex, mirror, and short tandem repeat (STR) motifs downstream from genes with 3′ ssDNA peaks in Deep crisis (Fig. 2C), proposing these structures as conducive to DNA lesions and replication stress in crisis (Makova and Weissensteiner 2023; Gadgil et al. 2024). Thus, KAS-seq signal peaks may be adopted as surrogate markers of both active transcription and potential sources of replication fork stalling in replicative crisis.

### Crisis reshapes the three-dimensional genome

**Figure 3.**
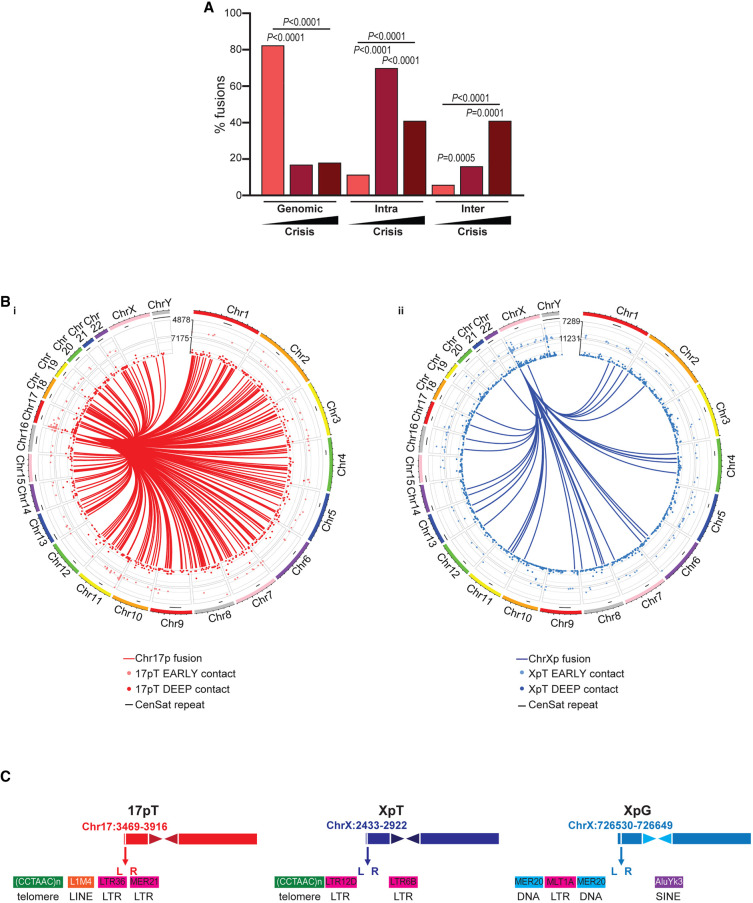
The changing profiles of telomere interactions and fusions during crisis transition. (*A*) The proportions of mapped telomere fusion sequences generated from Early (PD23) MRC5^E6E7^ crisis cells in this study that were classified as “genomic” (telomere fused to internal genomic locus), “intra” (intrachromosomal; telomeres of the same chromosome fused together) or “inter” (interchromosomal; telomeres of different chromosome fused together) are presented alongside comparable data for PD45 and PD49 MRC5^E6E7^crisis cells detailed in our former study (Liddiard et al. 2021). The black triangle and darker colored bars indicate the progression of replicative crisis. Statistical analyses employed the N − 1 χ^2^ method. (*B*) Circos plots (Yu et al. 2018) depicting all MRC5^E6E7^ Early- and Deep-crisis chromatin interactions with Chr17p (17pT; *i*) or ChrXp (XpT; *ii*) telomere-adjacent probes (17pT) determined using Capture-C presented as concentric perimeter tracks. Track height indicates signal intensity. Fusions between the Chr17p (*i*; red) or ChrXp (*ii*; blue) telomeres and genomic sites span the circle centers. Peripheral black lines underneath the chromosome identities demarcate the centromere and pericentromeric (CenSat) repeats. (*C*) Cartoons depicting the sequence contexts and human T2T-CHM13/hs1 genomic reference (Nurk et al. 2022) coordinates of the Chr17p (17pT), ChrXp (XpT), and internal genomic control (XpG) probes used in the Capture-C experiments. L and R denote the positions of the dual oligonucleotide probes at each location (for details, see Supplemental Table S1). Adjacent DNA repeats are represented by colored blocks. (LTR) Long terminal repeat, (LINE) long interspersed repeat, (SINE) short interspersed repeat, and (DNA) DNA repeat.

Telomere fusions amplified from MRC5^E6E7^ displayed progressively increasing proportions of longer-range inter- than intrachromosomal telomere–telomere rearrangements (Fig. 3A) with crisis transition. Conversely, the incidence of telomere–telomere (both intra- and inter-) fusions in Early-crisis samples was far lower than in stochastic genomic rearrangements, likely reflecting the more limited reservoir of eroded and fusogenic telomeres at this stage. Telomere-genomic recombinations may occur in the absence of telomere erosion through synapsis of DSBs in both locations. To understand the differential interactions of functional and eroded telomeres and their potential contributions to malignant recombination (Fig. 3B; Supplemental Fig. S6A,B), we performed Capture-C in Early- and Deep-crisis MRC5^E6E7^ using Chr17p (17pT) and ChrXp (XpT) telomere-adjacent capture probes and a nontelomeric control probe (XpG) (Fig. 3C; Supplemental Fig. S2).

**Figure 4.**
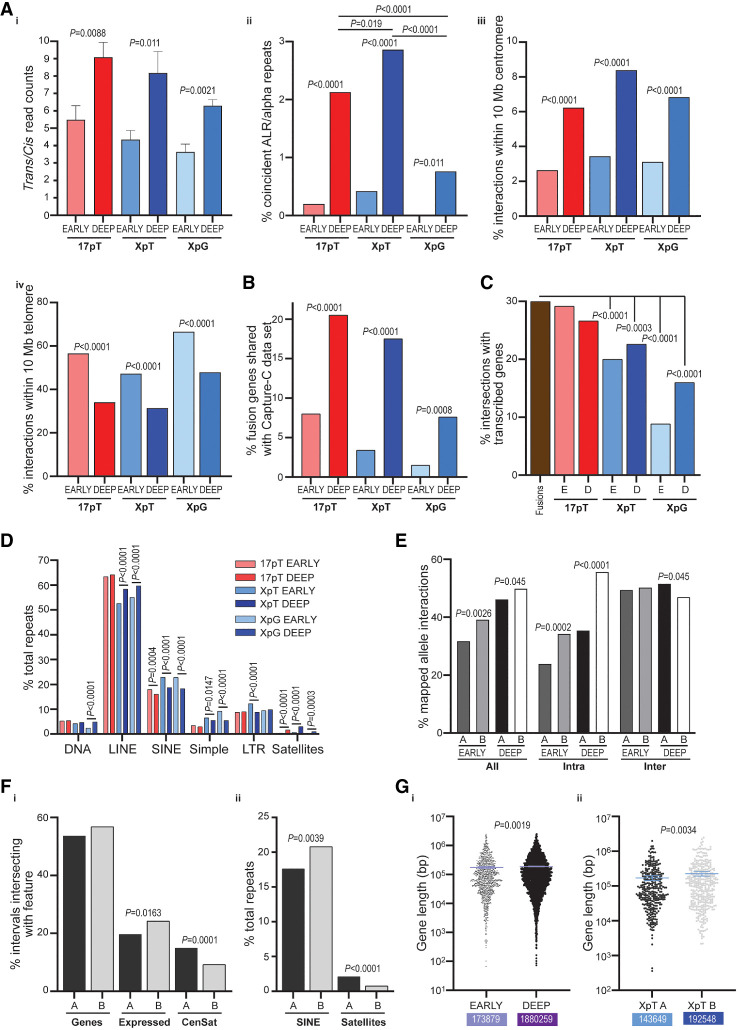
Crisis represents a transition in chromatin state and long-range chromosomal interactions. (*A*, *i*) Normalized read counts for all long-range *trans* compared with local *cis* chromatin Capture-C interactions determined by the Capcruncher pipeline. Triplicate samples of the Early- and Deep-crisis MRC5^E6E7^ sampling points using the Chr17p and ChrXp telomere-adjacent (17pT; XpT) and the ChrXp internal genomic (XpG) probes are displayed as means with 95% confidence intervals (CIs), analyzed using paired parametric *t*-tests. The mean sample percentages of interactions captured by the 17pT, XpT, and XpG probes in Early and Deep crisis that overlap with ALR/alpha satellite repeats (*ii*), are within 10 Mb of centromere (*iii*), or are within 10 Mb of telomere sequences (*iv*) are presented and compared using the N − 1 χ^2^ method. (*B*) The proportions of unique genes identified within crisis MRC5^E6E7^ telomere fusions sequenced in this and our former study (Liddiard et al. 2021) that overlap with genes captured by 17pT, XpT, or XpG probes in chromatin Capture-C experiments are shown and evaluated using the N − 1 χ^2^ method. (*C*) The proportions of genomic intervals associated with telomere fusion and chromatin Capture-C data sets that intersect with genes expressed in comparable crisis stage MRC5^E6E7^ samples (Liddiard et al. 2021) were computed using BEDTools intersect intervals (Quinlan and Hall 2010). Intersections with MRC5^E6E7^ telomere fusions were compared with intersections of each individual 17pT, XpT, or XpG probe Capture-C data set using the N − 1 χ^2^ method. (E) Early crisis, (D) Deep crisis. (*D*) The proportions of genomic intervals derived from chromatin Capture-C data sets (17pT, XpT, and XpG probes; Early- and Deep-crisis stages) that intersect with DNA repeats defined by RepeatMasker (*x*-axis) (Smit et al. 2013) are presented. The order of the data sets displayed corresponds to the adjacent key. (DNA) DNA repeat, (LINE) long interspersed repeat, (SINE) short interspersed repeat, and (LTR) long terminal repeat. Evaluation of differences employed the N − 1 χ^2^ method. (*E*) XpT chromatin Capture-C interactions were categorized according to the presence or absence of single-nucleotide variants (SNVs) characteristic of the long (A) or short (B) ChrXp telomere allele (Baird et al. 2003). The percentages of “all” and “intra”- or “inter”-chromosomal XpT interactions are compared for the Early- and Deep-crisis stages using the N − 1 χ^2^ method. (*F*) The proportions of genomic intervals associated with ChrXp A or B total allelic contacts that intersect with genes, expressed genes, and centromeric satellite (CenSat) repeats (*i*) or SINE and satellite DNA repeats (*ii*; delineated by RepeatMasker) (Smit et al. 2013) were analyzed using the same methodology. (*G*) The lengths of all genes (log_10_ scaling; means annotated within boxes *below* the *x*-axis) captured by the 17pT and XpT subtelomere probes at Early- and Deep-crisis stages (*i*) and the XpT A or B alleles at both crisis stages (*ii*). Means with 95% CI were compared using unpaired nonparametric Mann–Whitney *U* tests.

We observed robust elevations in long-range (trans) interactions (Fig. 4Ai) from both telomere-adjacent (17pT and XpT) and genomic (XpG) capture probes (but not unrelated capture probes targeting five distinct genes) (Supplemental Fig. S6C) with advancing crisis. This *cis*-to-*trans* shift was accentuated by a pronounced increase in centromeric contacts (Fig. 4Aii,iii) and a complementary suppression of telomere-proximal connections (Fig. 4Aiv), potentially linked to telomere shortening (Supplemental Fig. S1; Baur et al. 2001).

Subsets of genes interacting with telomere-adjacent probes were conjointly detected as telomere fusions in crisis MRC5^E6E7^ (Fig. 4B), suggesting recombination is, in part, related to the probability of stochastic contact between distant sites. Gene overlaps were functionally enriched in transmembrane signaling networks (Supplemental Fig. S6D) critical to the DNA damage response (DDR) (Maliszewska-Olejniczak and Bednarczyk 2024), indicating that genomic rearrangements incorporate genes actively expressed and required for crisis-cell viability. This creates a potential liability, whereby ongoing expression and reliance on these pathways increase the likelihood that further rearrangement or mutation compromises essential cellular processes. Intersections between Capture-C data sets and genes expressed by crisis MRC5^E6E7^ cells were consistently lower than for our collection of MRC5^E6E7^ telomere fusions but exhibited marked probe-dependent effects (Fig. 4C). Thus, transcription is less explicitly associated with subtelomere interactions than with fusions. Underlying associations with DNA repeat classes revealed only a dominance of LINE repeats at captured sites, with reductions in SINE and satellite sequences observed for all probes (Fig. 4D) in Deep crisis. Reassuringly, we detected no spurious enrichment of LTR sequences despite the prominence of these repeats at the chromosome ends (Fig. 3C).

Exploring the totality of Capture-C gene contacts, we found Deep-crisis subtelomere interactions to be specifically enriched in sodium transport genes (Supplemental Fig. S7A), and so we queried the ICGC/TCGA pancancer database (ICGC/TCGA Pan-Cancer Analysis of Whole Genomes Consortium 2020) for cognate gene abnormalities in cancer patients. We discovered aberrations in 70% patients (1808 of 2583 patients sampled), with amplifications being the most prevalent (Supplemental Fig. S7B). Variations in six of these genes (Supplemental Fig. S7C) had significant impacts for patient survival, linking the altered chromatin configurations we observed during crisis transition with cancer progression.

MRC5 cells have ChrXpYp alleles with disparate TLs (Supplemental Fig. S1C), enabling the longer (A) and shorter (B) alleles to be distinguished through heterozygous single-nucleotide polymorphisms in the telomere-adjacent DNA (Baird et al. 1995). This allelic asymmetry allows TL-dependent effects on long-range subtelomeric interactions to be assessed independently of crisis stage or replicative age, as both long and short telomere alleles can be simultaneously tracked within the same crisis cells. The shorter telomere (B) allele contributed to the greater proportion of intrachromosomal interactions throughout crisis compared with the longer telomere (A) allele (Fig. 4E), whereas no differential interchromosomal association was discerned. Both alleles echoed the diminished telomere-proximal contacts in Deep crisis (Supplemental Fig. S8A) observed with the wider data sets (Fig. 4Aiv) but only a robust association between the long allele and centromeres at the same stage (Supplemental Fig. S8B). Overall, the shorter (B) telomere allele displayed elevated intersections with expressed genes (Fig. 4Fi) and SINE repeats (Fig. 4Fii; Supplemental Fig. S8C) in contrast to the longer (A) telomere allele that had proportionally more interactions with centromere satellite (CenSat) and satellite repeats. These observations infer compartmentalization of extended repeat tracts within the nucleus, abrogated by telomere shortening that confers extraordinary mobility (Cho et al. 2014) and capacitates fusion with sister chromatids and genomic DSB (Liddiard et al. 2016). The increased mean lengths of genes captured in Deep crisis (Fig. 4Gi) or with the shorter B allele (Fig. 4Gii) is consistent with longer genes precipitating replication stress (Wei et al. 2016) and DSBs that promote cosegregation with deprotected telomeres. These results also corroborate recent findings of allele-specific chromatin organization (Irastorza-Azcarate et al. 2025).

We performed XSTREME motif discovery and enrichment analysis (Bailey 2021) in sequences flanking Capture-C probe interactions with genes also disrupted by telomere fusions (Fig. 4C) to identify organizing factors (Supplemental Fig. S9). Binding sites for chromatin insulator loop factors (including ZNF770, ZNF335, ZFX, and PITX2) (Trieu et al. 2020) were among the most enriched motifs, pinpointing topological boundaries as sites of particular vulnerability in the evolving crisis genome. Binding motifs for the inflammatory modulators BCL6 (Sawant et al. 2012) and EGR3 (Baron et al. 2015; Ogbe et al. 2015) were exclusively enriched in sequence overlaps with the telomere-adjacent capture probes and not with XpG or coding sequence probes. RNA-seq and KAS-seq data confirmed expression of *BCL6* and *EGR3* genes in MRC5^E6E7^ cells, propounding crisis-activated transcription (and the consequential disruption of this) as being instrumental in stress-induced genome reorganization (Dimitrova et al. 2008; Baron et al. 2015; Cardenas et al. 2017; Arnould et al. 2023).

### Cellular stress drives a shift in eccDNA profiles

**Figure 5.**
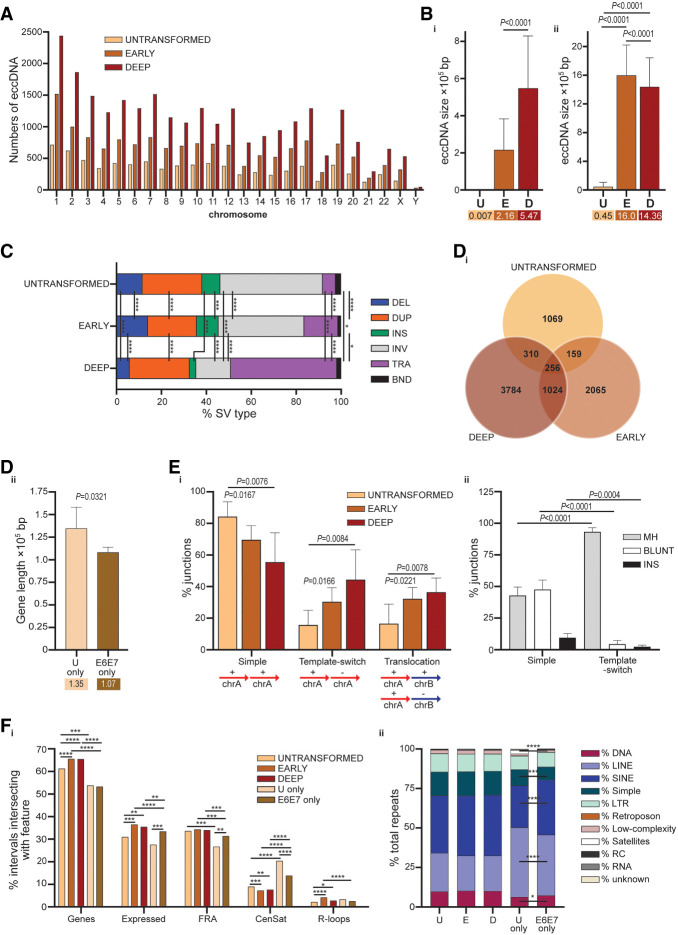
Distinct quantities and characteristics of extrachromosomal circular DNA are detected in untransformed and crisis MRC5 cells. (*A*) The quantities of all sequenced eccDNA junctions amplified from Untransformed MRC5 (U; six replicas) and Early (nine replicas) and Deep (six replicas) crisis MRC5^E6E7^ cells are displayed by chromosome. (*B*) Bar chart displaying mean sizes in base pairs with 95% CI for Illumina short-read sequenced (*i*) or Pacific Biosciences (PacBio) long-read sequenced (*ii*) simple eccDNA. Mean values × 10^5^ bp are displayed in colored boxes *below* the bars. Statistical evaluation was by Mann–Whitney unpaired nonparametric *U* tests. (*C*) Stacked chart displaying the relative proportions of distinct structural variants (SVs) called from the PacBio long-read eccDNA sequencing data for Untransformed MRC5 and Early- and Deep-crisis MRC5^E6E7^ cells. (DEL) Deletions, (DUP) duplications, (INS) insertions, (INV) inversions, (TRA) translocations, and (BND) break-end rearrangements that cannot unambiguously be categorized as a single canonical SV. Vertical black lines connect data sets evaluated using the N − 1 χ^2^ method, with results displayed as (*) *P* < 0.05, (**) *P* < 0.01, (***) *P* < 0.001, (****) *P* < 0.0001. (*D*, *i*) Venn diagram showing the numbers of direct gene overlaps among the eccDNA derived from Untransformed MRC5 and Early- or Deep-crisis MRC5^E6E7^ cells. (*ii*) The lengths of genes (means annotated below the *x*-axis) uniquely identified within genomic intervals assigned to eccDNA from Untransformed (U only) or transformed (E6E7 only) MRC5 cells are displayed in a bar chart with means and 95% CI. Mean values × 10^5^ bp are displayed in colored boxes *below* the bars. Differences were assessed by unpaired parametric *t*-tests with Welch's correction for unequal standard deviations (SD). (*E*, *i*) The proportions of eccDNA junctions amplified from Untransformed and Early- and Deep-crisis MRC5 samples that comprise DNA segments derived from the same strand (“Simple”) or opposing strands (“Template-switch”) or distant chromosome locations (“Translocation”) are depicted as a bar chart of means with 95% CI and compared using parametric unpaired *t*-tests with Welch's correction. A schematic depicting DNA orientation at the eccDNA junctions (ChrA, ChrB) is shown *below* the chart. (*ii*) Simple and template-switch junctions sequenced from all samples were examined for microhomology usage >1 bp (MH; pale gray) and inserted DNA sequences >1 bp (INS; black) or blunt joins (BLUNT; white). The proportions of junctions displaying each feature are presented in a bar chart and evaluated using unpaired nonparametric Mann–Whitney *U* tests. (*F*, *i*) Genomic intervals for eccDNA derived from Untransformed MRC5, Early- and Deep-crisis MRC5^E6E7^, or subsamples comprising Untransformed-only (U only) or transformed-only (E6E7 only) were intersected with the features indicated on the *x*-axis. (Genes) All coding sequence, (Expressed) genes expressed in crisis MRC5^E6E7^, (FRA) fragile sites, (CenSat) peri/centromeric repeats, and (R-loops) trinucleotide DNA structures. Pairwise comparisons were performed using the N − 1 χ^2^ method and results are displayed as (*) *P* < 0.05, (**) *P* < 0.01, (***) *P* < 0.001, (****) *P* < 0.0001. (*ii*) The same eccDNA sample (indicated on the *x*-axis) genomic intervals were intersected with DNA repeats (defined by RepeatMasker) (Smit et al. 2013), and the proportions of each repeat class (*right*-hand color key) are displayed in a stacked bar chart. The notable differences between the discrete Untransformed-only (U only) and transformed-only (E6E7 only) were evaluated with the N − 1 χ^2^ method, as in *i*. (RC) Rolling circle repeats, (RNA) grouped representation of all classes of RNA repeats: rRNA, tRNA, snRNA, scRNA, and srpRNA.

Telomere fragments (Lovejoy et al. 2012; Geiller et al. 2022) and telomere fusions (Muyas et al. 2024) have been detected as circular DNA species, and so, we next sought to clarify the interrelationship between telomere dysfunction and these specialized SVs. To ascertain the hierarchy and salient features of events (Tatman and Black 2022), we first purified, amplified, and sequenced eccDNA (Møller et al. 2016; Møller 2020; Chen et al. 2022) from Early- and Deep-crisis MRC5^E6E7^ fibroblasts, as well as the parental Untransformed cells (Supplemental Fig. S3). The chromosomal locations of all eccDNA junctions sequenced in four independent experiments are displayed in Figure 5A and Supplemental Figure S10A and are enumerated in Supplemental Table S1. Contrary to the high CN DNA circles serving as oncogenic drivers of specific cancers (Schwab et al. 1983; Sauter et al. 1996), we did not identify breakpoints common to replica samples (Yi et al. 2022), advocating transient and labile eccDNA formation resemblant of the unique telomere fusions that arise during crisis (Liddiard et al. 2016). Furthermore, the eccDNA we sequenced contained both coding and intergenic sequences, rather than entire genes, and varied in length from hundreds to millions of base pairs, affirming the capture of diverse eccDNA species (Yang et al. 2022).

Consonant with the increased abundance of eccDNA amplified from MRC5 progressing deeper into replicative crisis (Supplemental Fig. S3Biii), we measured escalations in both mean eccDNA sizes (Fig. 5B) and complexity (Fig. 5C). Long-read sequencing revealed a substantial expansion in the proportion of eccDNA comprising DNA translocations for the Deep-crisis samples that mirrors the increasing disorganization of the crisis genome (Cleal et al. 2019) and the enhanced capacity for long-range interactions (Fig. 4A). Thus, eccDNA derived from Untransformed cells are evidently distinct from those detected in transformed MRC5^E6E7^ cells. Indeed, intersections across data sets demonstrated that both the overall eccDNA profiles (Supplemental Fig. S10B) and genes captured within eccDNA (Fig. 5Di) showed greater concordance between Early and Deep crisis than between Untransformed samples and either crisis stage.

Although eccDNA were smaller in Untransformed cells than in crisis cells (Fig. 5B), the genes within the genomic intervals from which they originated had longer mean lengths (Fig. 5Dii), suggesting that intermediates of late replication may circularize during normal cell division (Møller et al. 2018; Berkemeier et al. 2025; Eugen-Olsen et al. 2025). Functional enrichments in related gene networks (cell junctions and transmembrane signaling) were determined for both direct overlaps (Fig. 5Di; Supplemental Fig. S11A) and shared genomic intervals (Supplemental Figs. S10B, S11B), with crisis sample genes also reflecting metabolic divergence. Together, these observations suggest that eccDNA-associated gene content reflects cellular processes engaged during crisis, including changes in energetic demand (Turner et al. 2017).

Because eccDNA may be generated by homology-dependent and non-homology-dependent DNA repair (Cohen et al. 2006; Gadgil et al. 2024; Kang et al. 2025), we investigated eccDNA junction sequences for mechanistic insights. A prominent transformation in junction attributes was evident (Fig. 5Ei), with progressive increases in the proportions of both template-switch (opposite strand orientation) junctions and translocations, aligned with the expansion in translocations observed for the crisis compared with Untransformed samples (Fig. 5C). Although template-switched eccDNA junctions almost exclusively presented sequence microhomologies (MH) (Fig. 5Eii; Tatman and Black 2022), simple (same orientation) events were represented by blunt junctions as well as those bearing insertions or MH. These observations may signify a prevalence of replicative- or synthesis-dependent recombination processes underpinning template-switched events over larger genomic distances in contrast to nonhomologous end-joining (NHEJ) localized at simple junctions (Lee et al. 2007; Sotiriou et al. 2016; Zhu et al. 2017; Kishkevich et al. 2022; Yang et al. 2023; Hu et al. 2024; Qin et al. 2025). Thus, eccDNA provide a molecular readout of the deranged crisis genome.

Intersections of eccDNA genomic origins with sequence features (Fig. 5F; Supplemental Fig. S12) clearly distinguished events amplified from Untransformed MRC5 cells. In particular, the genomic intervals exclusive to Untransformed eccDNA (“U only”) commanded greater association with centromeric (Fig. 5Fi) and satellite, simple, and LINE DNA repeats (Fig. 5Fii; Supplemental Fig. S12A) and fewer intersections with expressed genes and SINE repeats. Conversely, crisis-derived eccDNA more commonly originated in coding sequence (including fragile sites) and SINE DNA repeats, an attribute shared with telomere fusions (Supplemental Fig. S12A). Associations with DNA repeats proved an effective means of sample stratification (Supplemental Fig. S12B), effectively separating crisis from Untransformed samples, with comparable variance contributions from each DNA repeat class.

Overall, these discoveries support divergent mechanisms for synthesis of eccDNA in Untransformed (normal) and transformed (crisis) cells. Untransformed cells exhibit low levels of small eccDNA derived from long genes and repeat tracks, likely reflecting end-joining-mediated circularization of replication byproducts. Crisis marks an upsurge in eccDNA formation, conceivably mediated by both replicative and homology-independent DNA repair processes and more often occurring within actively transcribed chromatin.

### eccDNA signatures of cellular stress and malignancy

To assess the relevance of our observations in the MRC5^E6E7^ crisis model to human disease, we investigated the impact of environmental stress (Supplemental Fig. S13) and established malignancy (glioma) (Supplemental Fig. S14) for eccDNA profiles. Hypoxia influences both transcription (Johnson et al. 2008) and replication stress (Kindrick and Mole 2020; Ma et al. 2023), so we first explored the effects of transient (24 h) exposure to low (2%) compared with standard (20%) tissue culture oxygen (O_2_) levels on eccDNA production and telomere fusions in MRC5 cells. Low O_2_ resulted in higher eccDNA yields in the Untransformed cells (Supplemental Fig. S13Ai), homogenizing these to Early-crisis levels. Accordingly, we determined a substantial (33%) enlargement in mean eccDNA size under low-O_2_ conditions (Supplemental Fig. S13Aii), implicating cellular stress in the generation or maintenance of larger eccDNA. We also measured an increased incidence of simple eccDNA junctions (Supplemental Fig. S13Aiii) closer to the chromosome ends (Supplemental Fig. S13Aiv), and with enhanced associations with genes (Supplemental Fig. S13Bi), simple and low-complexity repeats (Supplemental Fig. S13Bii) relative to standard-O_2_ conditions. The limited sizes of the data sets constrained our ability to detect direct overlaps between cognate fusion and eccDNA samples (Supplemental Fig. S13Ci). Nonetheless, analysis at the gene level revealed shared amplifications across both SV types (Supplemental Fig. S13Cii). We discerned 24 genes (1.53% of 1571 total) in common between fusion and eccDNA data sets, with overlaps between the Early-crisis and low-O_2_-treated samples the most abundant. Examples of genes conjointly detected fused to telomeres and within eccDNA are displayed in Supplemental Figure S13D, in which *VWF* and *CCSER1* appeared as fusions and eccDNA in Early-crisis low-O_2_-treated samples, TTN was a fusion and eccDNA in standard-O_2_-treated samples, and *EXT1* was identified in fusions from both Early-crisis low- and standard-O_2_-treated samples and an eccDNA in Untransformed standard-O_2_-treated samples. These results indicate a degree of parity among genes susceptible to DSB and recombination by quite different processes. However, the lack of junction proximity or similarity precludes definitive assignment of sequential or related mechanisms at present.

Gliomas, particularly glioblastoma multiforme (GBM), display considerable intratumoral heterogeneity and therapy resistance that may be conferred by eccDNA-driven oncogene expression (Noorani et al. 2022). We obtained unfixed tumor and matched-adjacent tissue from three patients with astrocytoma (WHO grade 2/3; *IDH1*, *ATRX*, and *TP53* mutated) (Louis et al. 2021) and three patients with GBM (WHO grade 4; *TERT* promoter mutated) (Supplemental Table S2). *ATRX* mutations are associated with decompressed chromatin at telomeres (Episkopou et al. 2014; Clynes et al. 2015) that is permissive of alternative lengthening of telomeres (ALT) and the production of extrachromosomal DNA containing telomere repeats (Lovejoy et al. 2012). We initially tested the capacity of eccDNA sequencing to detect such extrachromosomal telomere circles by evaluating the prevalence of telomere repeats (three contiguous hexamers) within SVs (Supplemental Fig. S14Ai). Notwithstanding intersample variability, eccDNA derived from astrocytoma patients exhibited a fourfold higher incidence of telomere repeat modules compared with the GBM patient samples, despite having similar eccDNA content overall (Supplemental Fig. S14Aii). We also detected examples of directly inverted telomere repeats in one astrocytoma patient sample that may indicate the circularization of pre-existing telomere fusions (Muyas et al. 2024). Junction analyses revealed salient disease-specific characteristics (Supplemental Fig. S14B), with evidence of enhanced MH usage in astrocytoma-derived eccDNA. Gene lengths were comparable across the samples (Supplemental Fig. S14Ci) but were located closer to the telomeres in eccDNA amplified from tumors compared with matched tissue samples (Supplemental Fig. S14Cii), even though TLs were not patently different (Supplemental Table S2).

Analyzing underlying genomic features exposed a hypoxic signature for the astrocytoma tumor samples (Goncalves et al. 2025) comparable with the low-O_2_-treated MRC5 cells. The astrocytoma (but not GBM) tumor samples shared more overlapping genomic intervals with the totality of the MRC5 low-O_2_ (2%) samples than the standard-O_2_ (20%) samples (Supplemental Fig. S14D). Repeat associations (Supplemental Fig. S14E) also mirrored those observed in low-O_2_ MRC5 samples, with increased intersections with simple, satellite, and low-complexity repeats (Yehuda et al. 2025) and reduced overlap with LINE elements. STRING functional protein interaction analyses revealed a single reactome pathway enrichment in VEGFA–VEGFR2 signaling (FDR 0.0125) (Supplemental Fig. S14Fi) characteristic of chronic hypoxia (Shweiki et al. 1992; Blouw et al. 2003; Godard et al. 2003) for the GBM tumor sample. Circularized fragments of oncogenes (*CRK*, *SRC*, *ALK*), DNA repair components (*FANCC*, *FAN1*, *MGMT*, *FANCE*, *MLH1*, *PARP8*, *HIPK2*), and immune effectors (*HLA-E* and *C2*) were detected in the GBM and astrocytoma (Supplemental Fig. S14F) samples, with potential impact for pathological progression. We additionally detected amplification of the *EGFR* gene in one GBM patient (Supplemental Fig. S14G) that was more extensive and exclusive than the recurrent punctate signal associated with simple repeats within the terminal intron. *EGFR* mutations and amplifications are key drivers of glioma (Libermann et al. 1985), and the same GBM patient was determined to have cytogenetic rearrangement of exons 14 and 15 of this gene (Supplemental Table S2). Thus, eccDNA profiles from patient samples capture key oncogenic processes driving malignancy.

### Targeted evaluation of genome instability

**Figure 6.**
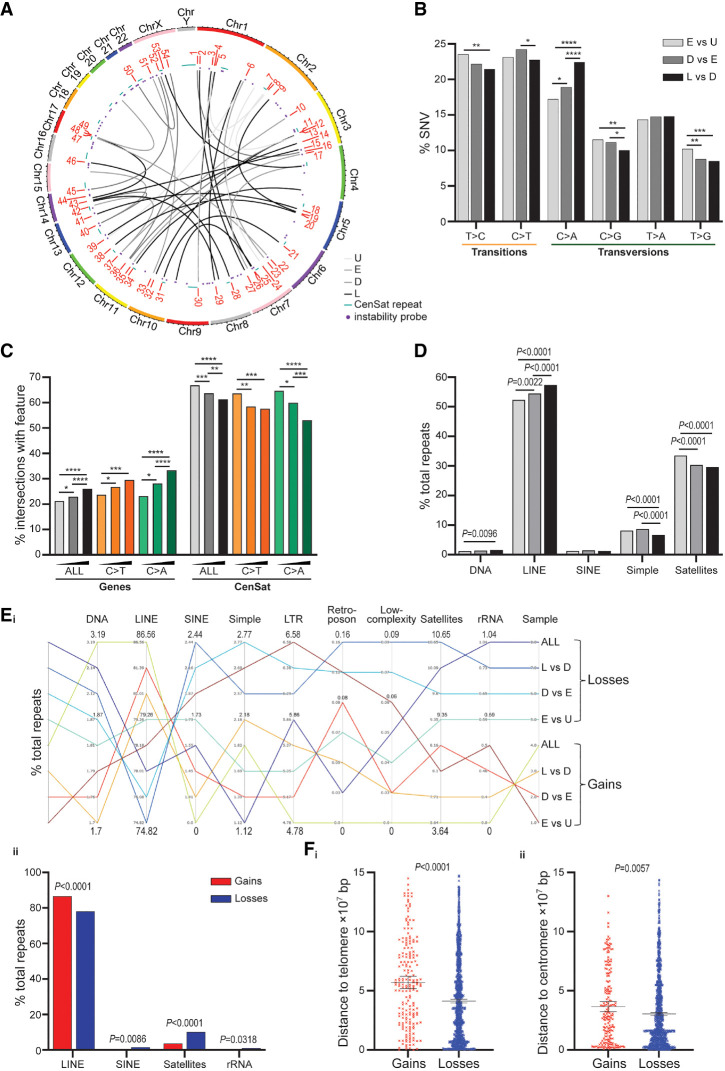
Mutational shifts with crisis progression. (*A*) The genomic distributions of 38 structural variants (SVs) called by Manta (Chen et al. 2016) from targeted capture sequence data for Untransformed (U; pale gray) and MRC5^E6E7^ undergoing replicative crisis depicted as Circos plot (Yu et al. 2018) links. (E) Early crisis, (D) Deep crisis, and (L) Late crisis. Colors are from mid-gray to black. Genes incorporated into the SVs are numbered and decoded in Supplemental Table S3. The chromosome identities, centromere positions (denoted by CenSat repeats), and locations of the genome instability probes featured on the targeted capture panel are indicated. (*B*) The percentages of all single-nucleotide variants (SNV) called using VarDict (Lai et al. 2016) for pairwise pseudo-tumor-normal (Early crisis vs. Untransformed, Deep vs. Early crisis, and Late vs. Deep crisis) comparisons of MRC5 samples sequenced following targeted capture are displayed. Transitions (purine–purine or pyrimidine–pyrimidine) are underscored in orange (*left*); transversions (purine ↔ pyrimidine), in green (*right*). Comparisons of SNV proportions in progressive samples were performed with the N − 1 χ^2^ method, and results are displayed as (*) *P* < 0.05, (**) *P* < 0.01, (***) *P* < 0.001, and (****) *P* < 0.0001. (*C*) The proportions of all SNV or specifically C > T transitions or C > A transversions identified in pairwise pseudo-tumor-normal MRC5 comparisons that coincide with coding sequence (Genes) or centromeric satellites (CenSat) are displayed in a bar chart and compared using the N − 1 χ^2^ method. (*D*) Overlaps between all SNVs called from progressive pseudo-tumor-normal MRC5 sample pairings and specific repeat elements (*x*-axis) are displayed as proportions of the total overlaps with repeats and evaluated in the same way (*P*-values recorded); (SINE) Short interspersed repeat, (LINE) long interspersed repeat. (*E*, *i*) A parallel plot displaying the proportions of specific DNA repeat elements (listed along the *top*) comprising the total repeats intersections with the altered copy number (CN) data sets indicated on the *right* (lower gains and upper losses for each pairwise sample comparison). (All) The genomic intervals common to all pairwise CN gains or CN losses. The upper and lower boundaries of the proportions are annotated at the *top* and *bottom* of the chart, respectively. (LTR) Long terminal repeat, (rRNA) ribosomal RNA repeats. (*ii*) A bar chart displaying the most salient differentials (evaluated using the N − 1 χ^2^ method) between the genomic intervals common to CN gains or CN losses with respect to DNA repeat associations. (*F*) The distance to the telomere on the same chromosome arm (*i*) or centromere (*ii*) of CN gains and losses common to all pairwise sample comparisons; statistical assessments by Mann–Whitney unpaired nonparametric *U* tests.

Using a custom-designed targeted capture gene and genomic features panel, we pursued indices of genome-wide instability accompanying replicative crisis. We employed Manta (Chen et al. 2016) to call SV in pseudo-tumor-normal sequential MRC5 sample pairs (Early vs. Untransformed, Deep vs. Early, Late vs. Deep). Filtering for “break-end” (SV) calls outside of the targeted capture probe regions with junctions unique to individual samples revealed 38 translocations (Fig. 6A) linking 54 genes from remote loci. SVs were most abundant in Late-crisis samples (Supplemental Fig. S15A) and notably coincident with fragile sites (Supplemental Fig. S15B), highlighting replication stress as a potential mechanism for the formation of these nonclonal rearrangements.

We next performed similar pairwise MRC5 sample evaluations of single-nucleotide variants (SNVs) for a more granular investigation of genome instability and mutational processes. Categorizing the nucleotide substitutions (Fig. 6B), we found that individual transitions were more common than any single transversion, but the overall proportion of transversions was greater for all samples and elevated in the latest crisis samples (Supplemental Fig. S15C). Recognizing that transversions may have more deleterious consequences compared with transitions (Freudenberg-Hua et al. 2003; Liang et al. 2021), we explored SNV that altered with crisis transit. Among all SNV classes, only C > A transversions showed a notable increase with crisis progression (Fig. 6B), as exemplified by the mutation observed within the first intron of *HAVCR2* (*TIM-3*) (Supplemental Fig. S16A). This SNV alters putative binding sites for regulators, including FOXQ1 (HFH1) (Supplemental Fig. S16B), which, concurrently with *HAVCR2*, is substantially upregulated in Late-crisis MRC5^E6E7^ cells (Supplemental Fig. S16C), affirming the possibility of functional consequences of such SNV during replicative crisis.

In accordance with the inclusion of peri/centromeric repeat targeted probes on the custom panel, we found that >60% all identified SNVs were coincident with CenSat repeats (Fig. 6C), although this proportion declined with crisis progression, consistent with the reductions in satellite and simple repeat associations (Fig. 6D). A reciprocal rise in the proportions of SNV within coding sequence was most evident for the C > A transversions (Fig. 6C), but the overall incidence within the promoter sequence was <1% for any sample pairing, as anticipated from the panel design. These altered SNV distributions reflect a shift from replication slippage (Saayman et al. 2023; Maruta et al. 2024) or alignment ambiguity within satellite sequence (Altemose et al. 2022) toward a more compelling manifestation of the deteriorating integrity of the crisis genome revealed by our multiomic analyses (Figs. 1–5). This was supported by evaluating the SNV classes within their trinucleotide context (Supplemental Fig. S17), resulting in mutational signatures resembling “aging” profiles (Alexandrov et al. 2020; Degasperi et al. 2022).

Although inferring absolute CN changes from targeted capture sequence data is problematic, we were able to evaluate relative copy-number variation (CNV) using the same pseudo-tumor-normal MRC5 sample pairings for sample normalization implemented in VarScan 2 (Koboldt et al. 2012). We assessed the overlap of SNVs and CNVs with sites of ssDNA breaks and DSBs from our previous data sets (including ssDNA peaks, expressed genes, eccDNA, and telomere fusions) (Fig. 1; Liddiard et al. 2021). These genomic features effectively distinguished SNV events from CNV gains and losses in samples progressing through replicative crisis (Supplemental Fig. S18A). CNV were less often coincident with R-loops and telomere fusions than SNVs but were more frequently within genomic intervals shared with crisis-derived eccDNA, demonstrating a relationship between sporadic SVs and more enduring gross CN changes.

CN losses were more commonly associated with simple, satellite, SINE, and rRNA repeats than with CN gains (Fig. 6E), conceivably explaining the reduced incidence of SNV within these sequences during crisis (Fig. 6C,D) owing to real changes in genomic content. Pertinently, we also measured 1.38- and 1.2-fold reductions in the distance of CN losses (compared with gains) to telomere (Fig. 6Fi) or centromere (Fig. 6Fii) repeats on the same chromosome arm, respectively. This altered spatial distribution is consistent with satellite repeat rearrangements and repeat contractions reported during crisis (Stults et al. 2009; Liddiard et al. 2022), which are known to exacerbate replication stress (Gadgil et al. 2020; Shaikh et al. 2022; Ma et al. 2023) and erroneous repair (Hastings et al. 2009; Xu et al. 2017). Although extrapolation from the targeted capture methodology is limited, our data support the illustration of intrinsic instability at long genes and repeat tracts, with accumulating disruption of coding sequences as crisis advances.

## Discussion

### Telomere-driven replicative crisis instigates global chromatin reorganization

We previously demonstrated that inflammatory gene activation accompanies telomere fusion and genome instability in four independent human fibroblast models of replicative crisis (Liddiard et al. 2021). Here, using orthogonal sequencing approaches (Fig. 1) in an MRC5 model, we have produced an integrated characterization of replicative crisis (Supplemental Fig. S18B), sampling earlier time points after HPV16 *E6E7* transduction and including Untransformed cell comparators to distinguish events driving entry into and progression through crisis.

Capture-C sequencing revealed replicative crisis to be a potent driver of expansive remodeling analogous to cellular differentiation rather than a more plastic stress response (Shanel et al. 2025). Telomere attrition reduces the density of proteins bound at the chromosome termini (Takai et al. 2010; Analikwu et al. 2025), establishing a more open chromatin configuration with crisis progression. Substantive chromatin reorganization was exemplified by the elevated long-range (trans) interactions and contacts between eroded telomeres and centromere repeats (Fig. 4A) in Deep-crisis MRC5^E6E7^ cells. Telomere fusions mirrored this extended engagement (Fig. 3A), with an increased prevalence of interchromosomal and concomitant reduction in local intrachromosomal recombinations with crisis progression. Allelic resolution showed preferential engagement of the shorter ChrXpYp telomere with coding sequence and intrachromosomal sites, whereas the longer telomere displayed increased associations with repeat tracks (Fig. 4F). This coalescence of the short telomere allele with expressed genes and the sister chromatid is consistent with our affirmation of this as the fusogenic allele in crisis (Liddiard et al. 2016) and the patent relationship between transcription, DSBs, and telomere fusions (Escudero et al. 2019; Liddiard et al. 2021).

Both TP53 (Scheffner et al. 1990) and RB1 (Dyson et al. 1989) function is abrogated in our MRC5^E6E7^ model of replicative crisis (Supplemental Fig. S1), impacting chromatin structure (Gonzalo and Blasco 2005) and cell cycle progression and DNA repair (Obanya et al. 2025). Chromatin decompaction attenuates the insulating capacity of telomeres, derepressing gene expression (Baur et al. 2001) and condensin-mediated DNA loop extrusion, thereby constraining the error-free resolution of telomere fusions (Analikwu et al. 2025) that occur with increasing frequency in crisis (Supplemental Fig. S1B). We additionally observed elevated incidence of larger and more complex eccDNA (Fig. 5) and nonclonal translocations (Fig. 6) at later stages of replicative crisis. Thus, telomere attrition and chromatin remodeling support the formation of alternative chromatin loops with extended genomic reach that enable synapsis of distant loci.

The release and redistribution around the genome (Stock et al. 2022) of DNA binding factors formerly sequestered within the telomere structure (Maillet et al. 1996; Platt et al. 2013) amplify the persistent DNA damage signaled by the eroded telomere (Supplemental Fig. S5; Cesare et al. 2013; Nassour et al. 2024). The release of chromatin fragments (including replication intermediates and eccDNA) into the cytosol (Zhang et al. 2021) and telomeric repeat-containing transcripts (TERRA) into exosomes compounds the inflammatory environment (Wang et al. 2015; Lex et al. 2020). Telomeric structural transformations further impact the transcriptome through relief from the repressive telomere position effect (TPE) (Baur et al. 2001; Rey-Millet et al. 2023) that regulates expression of critical mediators of aging and inflammation (Dong et al. 2021; Liddiard et al. 2021) enriched at the chromosome termini. Although our results distinguish telomere fusions from chromosomal interactions (Figs. 3, 4), loci represented in both data sets propose the DDR as a potent reorganizing force (Supplemental Fig. S5A; Wu et al. 2024a), with binding sites for stress response mediators including BCL6 and EGR3 being overrepresented in recombined sequences (Supplemental Fig. S9).

### eccDNA profiling reveals signatures of cellular stress and disease

The relative abundance and ease of eccDNA purification from crisis cells and patient samples make these SVs more attractive and robust indicators of genome instability compared with telomere fusions, particularly when input material is scarce. Notably, eccDNA derived from transformed MRC5^E6E7^ cells were quantitatively and qualitatively distinct from those amplified from Untransformed MRC5 (Fig. 5; Supplemental Figs. S10–S13), corroborating their potential as surrogates of malignancy (Bruhm et al. 2025). Lower yields of smaller eccDNA were harvested from Untransformed than from transformed cells (Fig. 5), and these were typified by simple junctions originating from longer genes and repeat elements that included centromeres and other satellite sequences. These characteristics suggest a constitutive NHEJ-mediated circularization (Zhu et al. 2017; Hu et al. 2024; Kang et al. 2025; Qin et al. 2025) of the byproducts of replication and transcription at challenging repeat tracks and late-replicating genes with limited impact for cellular function (Møller et al. 2018). In contrast, eccDNA amplified from crisis MRC5^E6E7^ captured shorter genes with greater proportions of template-switched junctions and long-range complex translocation events, compatible with our Capture-C data and suggestive of replicative repair (Sotiriou et al. 2016; Kishkevich et al. 2022; Zhang et al. 2023). Furthermore, we detected increased association with transcribed genes and SINE repeats, linking this transition to transcriptional reprogramming induced by crisis (Liddiard et al. 2021).

Regarding eccDNA as tangible indices of genome instability, we determined a sensitivity of profiles to environmental (Supplemental Fig. S13) and pathological stressors (Supplemental Fig. S14). Under conditions of transient low O_2_ (2%), eccDNA purified from Untransformed MRC5 approximated the yields and size distributions characteristic of crisis MRC5^E6E7^ eccDNA (Supplemental Fig. S13A), independent from effects on cell viability or proliferation. Cellular responses to mild hypoxia (Hu et al. 2003; Johnson et al. 2008; Kindrick and Mole 2020; Wu et al. 2022) resulted in eccDNA enriched in simple junctions capturing genes closer to telomeres, potentially reflecting NHEJ activity within sequences transcribed from the reservoir of inflammatory and immune regulators close to the chromosome ends (Lex et al. 2020; Lopes et al. 2021). Testifying to the replication stress exacerbated by hypoxia, eccDNA from cells cultured in low-O_2_ conditions were enriched in simple, satellite, and low-complexity repeat motifs that may challenge replication fork or transcriptional progression (Hammond et al. 2002; Chan et al. 2008; Yehuda et al. 2025), facilitating eccDNA biogenesis (Qin et al. 2025). Among our cohort of glioma patients (Supplemental Table S2), eccDNA properties distinguished astrocytoma from GBM pathology and tumor tissue from matched-adjacent samples (Supplemental Fig. S14). *ATRX*-mutated astrocytomas produced eccDNA enriched for telomeric and replication-stalling repeats (Clynes et al. 2015; Nguyen et al. 2017), with extended MH at junctions, consistent with replication stress and end-joining repair (Wimberly et al. 2013; Ngo et al. 2021). Whereas the astrocytoma tumor samples displayed significant parallels with MRC5 low-O_2_ cultures (Supplemental Fig. S14D; Goncalves et al. 2025), the GBM tumors evidenced functional gene networks characteristic of the hypoxic environment that drives this aggressive pathology (Supplemental Fig. S14F; Park and Lee 2022; Marallano et al. 2024). Thus, by analyzing eccDNA contents and junction features, we have been able to surmise both active gene networks and sites of replication stress. In a clinical setting, eccDNA profiling could likewise identify critical cancer dependencies for novel targeting strategies and therapeutic monitoring (Li et al. 2023; Wang et al. 2025).

### Interrelationship of SVs

We previously reported the assimilation of circular plasmid and mitochondrial DNA into telomere fusions (Liddiard et al. 2016), raising the possibility of eccDNA being captured by deprotected telomeres during crisis. Moreover, we have exposed the association between telomere fusions, eccDNA, and sites of CNVs (Supplemental Fig. S18; Escudero et al. 2019; Liddiard et al. 2021) symptomatic of DNA excision and extrachromosomal amplification (Von Hoff et al. 1992; Verhaak et al. 2019). Accordingly, we identified recurrent genes incorporated into telomere fusions and eccDNA amplified from parallel MRC5 cell cultures, confirming that destabilized loci are substrates for both forms of SVs (Supplemental Fig. S13D). With clinical relevance, we identified eccDNA comprising inverted telomere repeats amplified from a patient with *ATRX-*mutated astrocytoma, consistent with the production of telomeric circles in this cancer (Cesare and Griffith 2004). The concurrent amplification and cytogenetic rearrangement of *EGFR* observed for one GBM patient (Supplemental Fig. S14G) are also suggestive of successive or interdependent mutational processes. Although we did not identify shared junctions among distinct SVs in parallel analyses, our findings sustain the possibility of a common origin for telomere fusions and eccDNA in malignancy.

Overall, our integrated analyses reveal the critical contributions of telomere and centromere repeats to chromatin reorganization under conditions of replicative and environmental stress. We demonstrate the interdependence of telomere attrition, replication, and transcription in the collective translation of the cellular stress response into a three-dimensional structural variation within the evolving cancer genome (Supplemental Fig. S18B). Our data support a model of replicative crisis in which the inherent instability of long, late-replicating sequences is progressively compounded by increasingly complex long-range interactions that disrupt crisis-response genes, shaping both cancer genotype and phenotype. We additionally uncover the unrealized potential of eccDNA as biomarkers of pathological processes, even in the absence of clonal amplifications. These findings highlight novel diagnostic opportunities and provide mechanistic insights into genome instability in replicative crisis.

## Methods

### Cells and treatments

MRC5 cells were authenticated by STR profiling with the American Type Culture Collection (ATCC) and routinely screened for the absence of mycoplasma. Cells were cultured in Eagle's Minimum Essential Medium (EMEM) supplemented with 1× nonessential amino acids, 10% (v/v) FCS, 1 × 10^5^ IU/L penicillin, 100 mg/L streptomycin, and 2 mm glutamine and buffered with 0.2% NaHCO_3_ solution.

The crisis MRC5 cells were generated in our previous publication (Liddiard et al. 2021) using amphitropic retroviral vectors containing HPV16 *E6* and *E7* oncogenes (*E6E7*) and a neomycin-resistance cassette (*NEO*), as outlined previously (Bond et al. 1999). Selection of transduced and expressing MRC5^E6E7^ cells was by culture in the presence of Geneticin (G418) at 0.4 mg/mL. Cells were maintained at 70%–85% confluency, with PD calculated at each passage following cell counts using an NC-3000 image cytometer (Chemometec). The PDs indicated in this paper refer to population growth after the point of retroviral transduction because this constitutes a bottleneck event (Supplemental Fig. S1A). Crisis staging (Early, Deep, and Late) was assigned according to the appearance and accumulation of telomere fusions at multiple chromosome ends (Supplemental Fig. S1B) following telomere attrition (Supplemental Fig. S1C).

For most experiments, cells were cultured in 20% O_2_ and 5% CO_2_ at 37°C. For experiments comparing standard with low-O_2_ stimuli, parallel cell cultures of 7 × 10^5^ Untransformed MRC5 or Early-crisis (PD23) MRC5^E6E7^ were established in incubators with 20% or 2% O_2_, respectively, with nitrogen gas compensation and regular monitoring with a handheld gas analyzer (Geotech G100).

Cell cycle analyses were performed using the Chemometec fixed cell cycle DAPI assay and analyzed using Floreada.io software.

### Chromatin capture

Capture-C experiments were performed for MRC5^E6E7^ Early-crisis (PD23) and Deep-crisis (PD47) cells according to the method previously described (Downes et al. 2022, 2023). NlaIII restriction enzyme (NEB R0125) cleaved nuclear 3C libraries were prepared from each of three replicas of independent cell cultures of MRC5^E6E7^ cells for each time point (5 × 10^6^ cells each). Evaluation of library quality was made by agarose gel electrophoresis and qPCR. Libraries were sheared to 200 bp using an ME220 Covaris focused ultrasonicator and indexed using NEBNext multiplex oligos for Illumina (primer sets 1–3; E7335S, E7500S, and E7710S) in a modification of the NEBNext Ultra II DNA library prep kit for Illumina protocol. Each library was subjected to two rounds of capture hybridization with each HPLC-purified 5′ biotinylated viewpoint probe set (Merck) (Supplemental Table S1) and amplification ahead of 150 bp paired-end sequencing on an Illumina MiSeq. The stringent design of the viewpoint probes to capture interactions from the Chr17p and ChrXpYp (abridged to ChrXp throughout) subtelomeres was facilitated by the CapSequm tool (Davies et al. 2016). A nontelomeric control probe (XpG) was also designed within a noncoding locus 724 kb 5′ of the ChrXp subtelomere (XpT) probe for comparison with the subtelomere sequence context (Fig. 2F; Supplemental Fig. S2). Agilent TapeStation analyses were performed at multiple stages to verify library yields and size profiles.

Initial data analysis steps employed the CapCruncher software pipeline (Downes et al. 2022) to call genomic loci captured by each probe and produce BAM files for visualization using the Integrative Genomics Viewer (IGV) (Robinson et al. 2011). For evaluations of long-range interchromosomal interactions, IGV was used to delineate and exclude local *cis*-interactions from downstream analyses. A signal threshold of 500 was applied to filter the remaining capture sites to mitigate the contribution of nonspecific interactions to the analyses. Corroboration of the credibility of these *trans* interactions was provided by intersections with orthogonal data sets, including telomere fusion sequencing and eccDNA sequencing. BLAT was used to verify selected alignments (Kent 2002). Allelic segregation of the ChrXp data was achieved by partitioning subtelomere alignments according to definitive presence of single-nucleotide polymorphisms diagnostic of the long (A) or short (B) allele (Baird et al. 1995).

### KAS-seq ssDNA mapping

KAS-seq identification of ssDNA genomic locations was implemented according to the method of Lyu et al. (2022). Three Early-crisis (PD23) and Deep-crisis (PD47) MRC5^E6E7^ samples were labeled with N_3_-kethoxal, sheared to 200 bp with the Covaris ME220 ultrasonicator, and enriched following biotinylation using the AccuraDX KAS-direct ssDNA labeling and enrichment kit. Sequencing libraries were prepared from enriched and nonenriched fractions of the same samples (used as input controls) using xGen ssDNA low-input DNA library prep (IDT 10009859) and xGen UDI primers (IDT 10005975). Sequencing employed the Illumina NextSeq 550 high-output 75 bp single-end sequencing platform to meet the protocol requirements of 30 million reads per library. Ultimately, one crisis sample resulted in a low signal sequencing output and was eliminated from the downstream analyses.

Preliminary read processing and alignment was executed with KAS-Analyzer (V1.0) (Lyu et al. 2023), and ssDNA signal peaks in the enriched Early- and Deep-crisis samples compared with the input controls were identified using epic2 (Stovner and Sætrom 2019). DiffBind (http://bioconductor.org/packages/release/bioc/vignettes/DiffBind/inst/doc/DiffBind.pdf; Ross-Innes et al. 2012) was used to perform differential analysis of consensus peaks, distinguishing peaks unique and common to the Early- and Deep-crisis states. An evaluation of false-discovery rates (FDR) and fold changes of the differences was also reported. Full commands and settings developed for this study are available at GitHub (https://github.com/e-coral/crisis_analyses) and in the Supplemental Material.

### Circle-seq

Glioma tissue samples were obtained from adult patients following informed consent, under the approval of the Welsh Neuroscience Research Tissue Bank (reference 19/WA/0058). Individual identifying information is not included in this paper. The methodology established by Møller (2020) was used to purify, amplify, and sequence eccDNA from Untransformed MRC5, MRC5^E6E7^, and glioma patient tissue samples (Supplemental Fig. S3). Approximately 7.5 × 10^5^ cells or 4 mm glioma tissue biopsies were harvested and processed for circular DNA extraction using the plasmid mini AX DNA kit (A&A Biotechnology). Digestion with Proteinase K was for 48 h at 700 RPM and 50°C; reduction of mitochondrial DNA (mtDNA) with MssI (PmeI) was for 1 h at 37°C; and elimination of linear genomic DNA involved five sequential doses of 25 U/sample plasmid-safe DNase (Biosearch Technologies) over 36 h at 37°C. Purified eccDNA were amplified by rolling circle amplification (RCA) with φ29 polymerase (Qiagen REPLI-g mini kit) over 60 h and were visualized by SYBR gold staining of electrophoresed products. Amplified eccDNA were sheared to 300–500 bp by ultrasonication with the Covaris M220 for Illumina 150 bp paired-end sequencing. Libraries from 500 ng sheared eccDNA were prepared utilizing the NEBNext Ultra II DNA library prep kit for Illumina and indices supplied in the NEBNext multiplex oligos for Illumina (primer sets 1–3). Agilent TapeStation analyses were performed at multiple stages to verify yields and size profiles. Samples were multiplexed and sequenced on the Illumina MiSeq.

Parallel Pacific Biosciences (PacBio) long-read sequencing experiments were also performed for Untransformed MRC5 and Early- and Deep-crisis MRC5^E6E7^ cells, as well as a single glioma tumor sample in pre- and post-RCA formats. To preserve long-read integrity, eccDNA samples were debranched with T7 endonuclease I for 1 h at 37°C, purified using Agencourt AMPure XP magnetic beads at a 1:1 ratio, and visualized by SYBR gold staining of electrophoresed products. Multiplexed amplicon libraries were prepared using the SMRTbell prep kit 3.0 (102-182-700) in conjunction with the SMRTbell barcoded adapter plate 3.0 (102-009-200). Verification and sizing of amplicons were conducted using an Agilent fragment analyzer system and Qubit 4 Fluorometer. One hundred nanograms of each adapted amplicon was pooled for PacBio sequencing using the Sequel II sequencing kit 2.0 (102-194-400) and SMRT cell 8m single-use tray (102-281-700). Sequencing was over 30 h, resulting in 15 Gb HiFi reads.

### Targeted capture sequencing experiments

For a focused investigation of genomic instability during replicative crisis, a custom targeted capture panel was designed incorporating 68 genes recurrently identified in our SV data sets; three with full-length probe coverage, 64 with exon-only (coding and UTR with 50 bp flanks) coverage, and one intronic MSI locus. The panel additionally contained probes for capturing potentially unstable repeat elements, including selected LINE, centromeric satellites, subtelomere sequences, tRNA and rRNA clusters, G-quadruplex binding motifs, and an MSI locus (details available on request). The custom panel was synthesized and quality validated by Twist Bioscience.

The Twist Bioscience cfDNA library preparation kit was used to prepare sequencing libraries from 50 ng each of MRC5 Untransformed and MRC5^E6e7^ Early-crisis (PD23), Deep-crisis (PD47), and Late-crisis (PD57) genomic DNA that had been sheared (Covaris) to a mean fragment size of 350 bp. Amplified libraries were subjected to a 16 h hybridization with the custom probe panel at 70°C in a thermocycler. Following targeted enrichment, multiplexed libraries were sequenced using Illumina NextSeq 550 high-output 300 cycle (2 × 150 bp) flow cell and reagents to achieve approximately 1500× coverage of the 8 Mb panel.

### Code availability

New computer code developed in this research is available at GitHub (https://github.com/e-coral) and as Supplemental Code.

## Data access

All raw and curated sequencing data generated in this study have been submitted to the NCBI BioProject database (https://www.ncbi.nlm.nih.gov/bioproject/) under the accession number PRJNA1181623.

## Supplemental Material

Supplement 1

Supplement 2
