# Supplementary material for: Telomere-driven replicative crisis is driven by large-scale changes in genomic architecture
Source: Genome Res. 2026 Aug;36(8):1529–47. doi: 10.1101/gr.281373.125 (PMC13431167; doi:10.1101/gr.281373.125)
Supplement: Supplement 1 [file Supplemental_Material.pdf]

Telomere-driven replicative crisis is driven by large-scale changes in genomic architecture

Kate Liddiard, Emmon Coral, Harsh Bhatt, Kez Cleal and Duncan M. Baird

Supplemental Material

Supplemental Figures S1-18

Supplemental Tables S1-3

Supplemental Methods

Supplemental Methods References

Supplemental Methods Table S1

Supplemental Code Index

Supplemental Figure S1

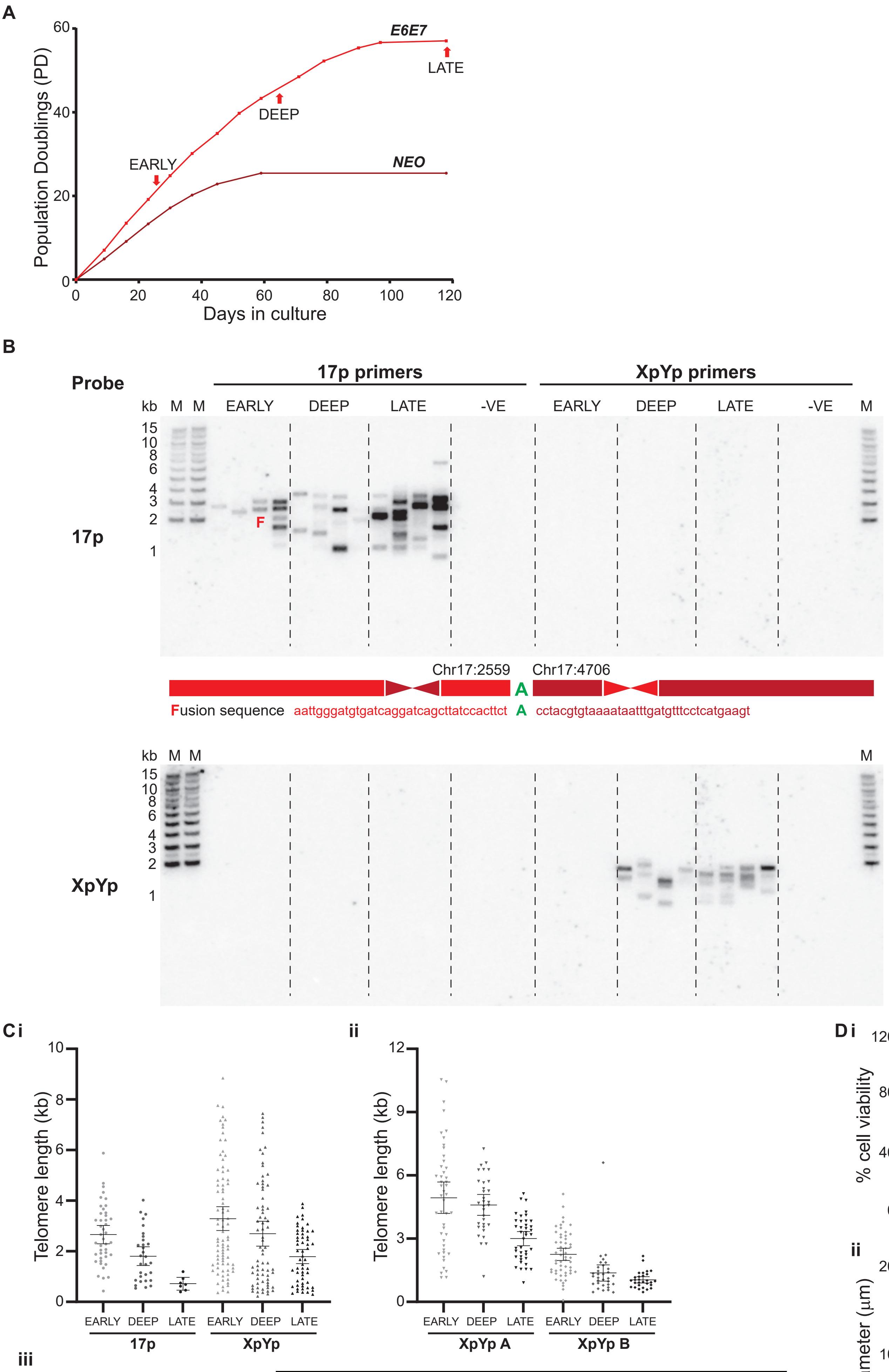

iii

	Mean TL (upper, lower 95% CI)			
**Crisis stage**	17p	XpYp total	XpYp A	XpYp B
EARLY	2.66 (2.30,3.02)	3.29 (2.82,3.76)	4.94 (4.19,5.69)	2.26 (1.97,2.55)
DEEP	1.81 (1.43,2.18)	2.70 (2.21,3.19)	4.6 (4.10,5.09)	1.37 (0.98,1.76)
LATE	0.72 (0.47,0.97)	1.79 (1.51,2.07)	3 (2.67,3.34)	1.05 (0.90,1.20)

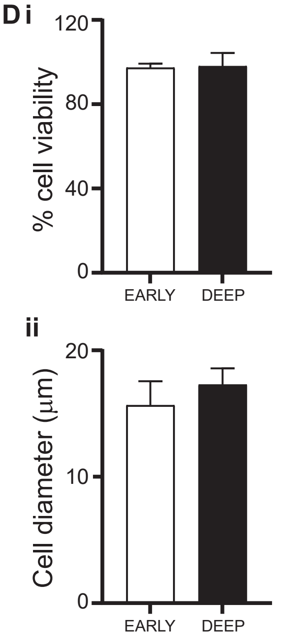

Supplemental Figure S1: MRC5 model of telomere-driven crisis

A Growth curves showing population doublings (PD) against time in culture for MRC5 human fibroblasts retrovirally-transduced with empty neomycin selection cassette (*NEO*) or coding sequences of human papillomavirus E6 and E7 proteins (*E6E7*) that abrogate TP53 and RB1 cell cycle and DNA damage response activity (Bond et al. 1999). Early (PD23), Deep (PD47) and Late (PD57) crisis sampling points (calculated from the point of transduction) are indicated with arrowheads for the $MRC5^{E6E7}$-transformed cells. **B** Imaged Southern blot revealing telomere fusions amplified using single orientation primers targeting unique Chr17p (left) and ChrXpYp (right) telomere-adjacent (subtelomere) sequences in Early, Deep and Late crisis $MRC5^{E6E7}$ cells, with 4 replica reactions per sample. The upper and lower panels display telomere fusion amplicons detected with Chr17p or ChrXpYp subtelomere-specific radiolabelled probes, respectively. M indicates lanes containing molecular weight markers with kilobase (kb) sizes noted to the left. A Sanger sequence-verified Chr17p intra-chromosomal fusion is annotated with 'F' and the corresponding sequence is indicated below the panel, with genomic junction positions (T2T CHM13v2.0/hs1 reference) detailed in black and a single adenine nucleotide junction insertion marked in green. **C** Single Telomere length measurements **i** at the Chr17p and ChrXpYp chromosome ends and **ii** for the long ChrXpYp A allele and short B allele in $MRC5^{E6E7}$-transformed cells at Early, Deep and Late crisis time points. **iii** Mean telomere lengths (TL) with 95% confidence intervals (CI) are detailed in the table below. **D** Mean **i** % cell viability and **ii** cell diameter (in micrometres) for exemplary (duplicate harvests) $MRC5^{E6E7}$ cultures at the Early (white) and Deep (black) crisis time points.

Supplemental Figure S2

A **17pT**

Chr17:3469-3916

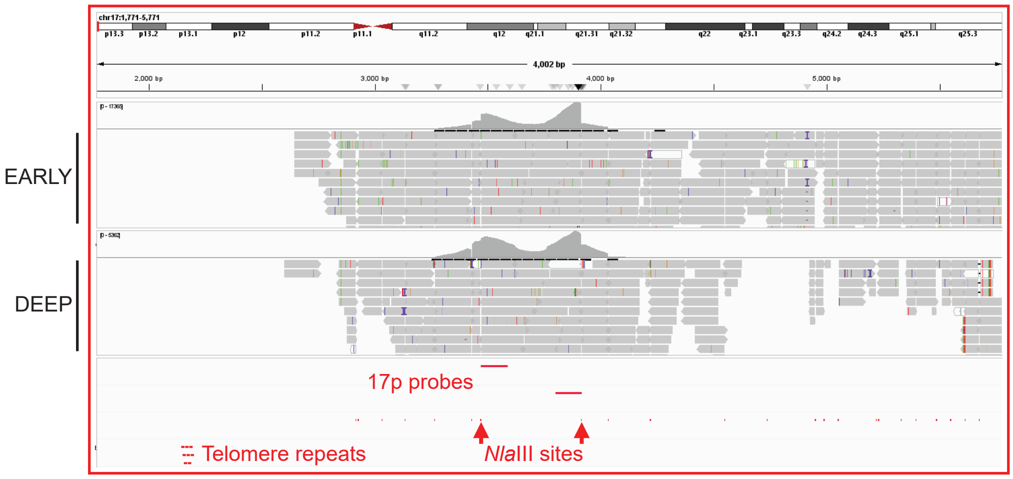

EARLY	DEEP
1.7	1.1
1.1	1.1
1.1	1.2

Chr17:1-100000

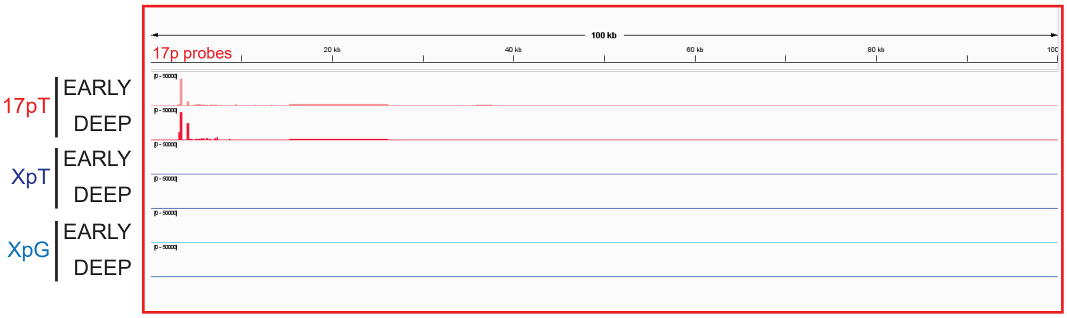

B **XpT**

ChrX:2433-2922

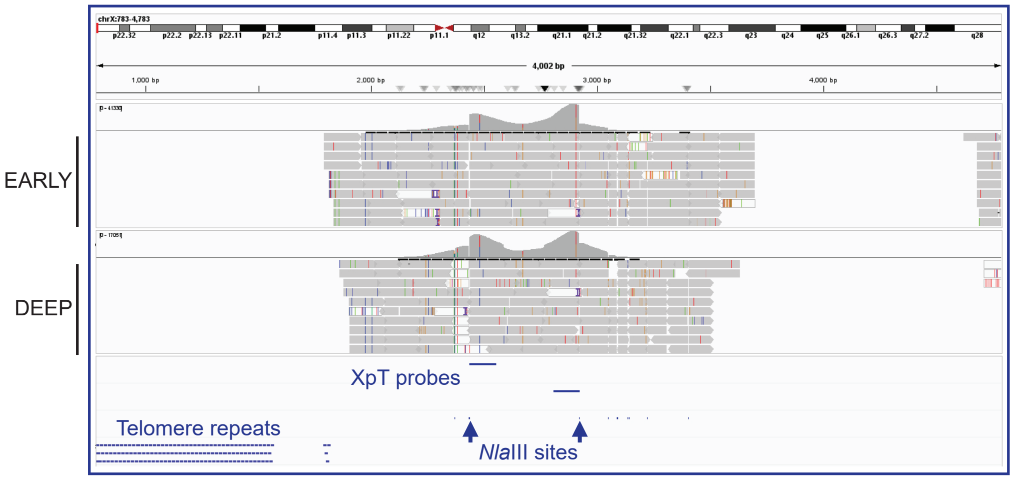

EARLY	DEEP
1.2	0.8
1.2	1.3
0.9	1.3

ChrX:1-100000

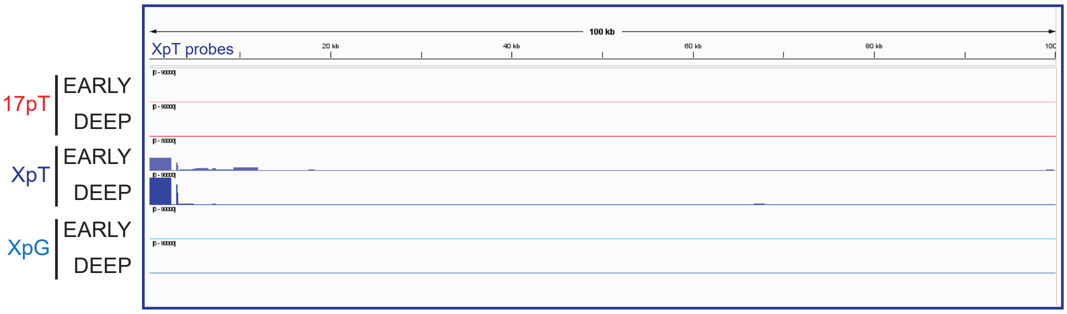

Supplemental Figure S2

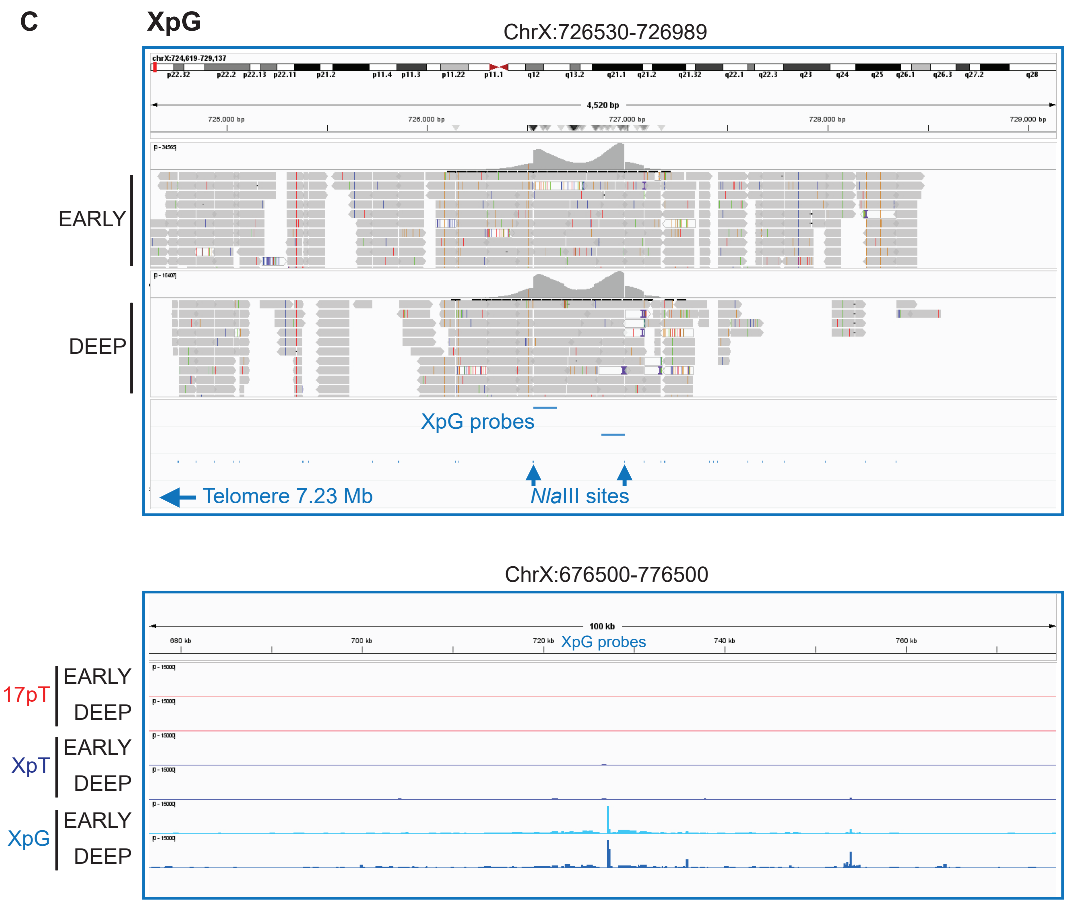

EARLY	DEEP
1.1	1
1.2	1.2
0.9	1.1

Supplemental Figure S2: Chr17p and ChrXpYp telomere-adjacent chromatin capture

Integrative Genomics Viewer (IGV) displays of sequence reads mapping **A** the Chr17p (17pT) and **B** ChrXp telomere-adjacent (XpT) and **C** ChrXp internal genomic (XpG) Capture-C (Downes et al. 2022) viewpoints (with T2T CHM13v2.0/hs1 human reference genomic locations indicated above each panel) designed to investigate long-range chromatin interactions between telomeres and the crisis genome in $MRC5^{E6E7}$ cells. Signal for one Early and one Deep crisis sample is shown in the upper panel for each probe set and the adjacent tables reveal the raw read pair counts per sample (in millions). The positions of the Capture-C probes and *Nla*III restriction enzyme sites employed in the assays are annotated, as well as the telomere repeat arrays for orientation. The lower panels corroborate the specificity of each probe set over 100 kb adjacent to the target hybridisation sites, with signal enriched only within the expected samples focussed at these sites.

Supplemental Figure S3

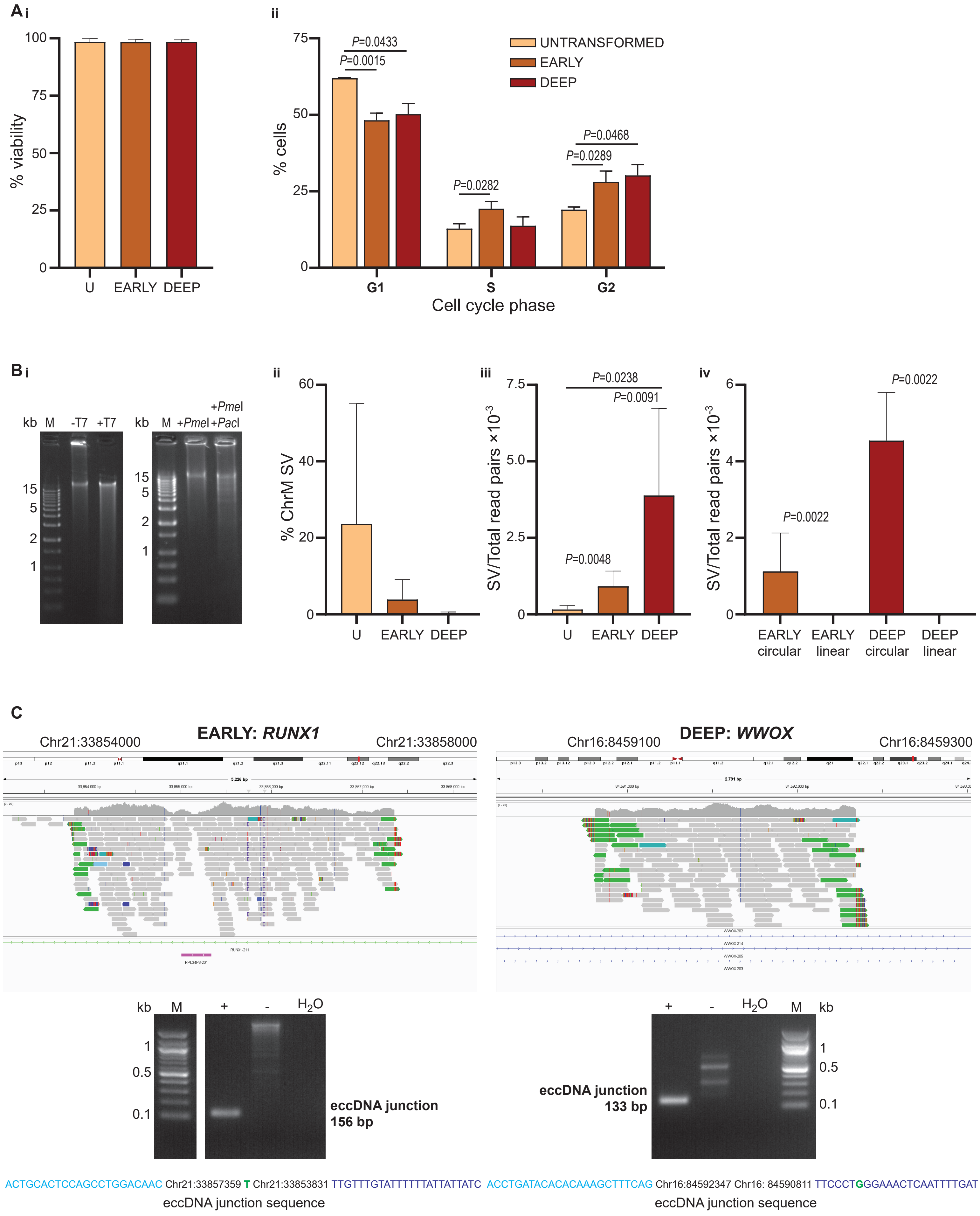

Supplemental Figure S3: Validation of extra-chromosomal circular DNA Circle-Seq approach

Ai Confirmation of comparable cell viability in samples from which eccDNA were amplified. Mean % cell viability for MRC5 Untransformed (U; 5 replicas) and Early (PD23; 12 replicas) and Deep (PD47; 5 replicas) crisis samples with 95% CI. **ii** Cells in Deep crisis display the anticipated expansion of the G2 cell cycle fraction. Cell cycle fractions for Untransformed MRC5 (2 replicas), Early (4 replicas) and Deep (2 replicas) crisis samples assessed using Floreada.io. Data were compared using unpaired parametric *t*-tests. **Bi** Amplification of legitimate eccDNA following extensive digestion of linear nuclear DNA was verified by retention of high molecular weight species following debranching and cleavage with restriction enzymes that degrade mitochondrial DNA. Agarose gel electrophoresis images of native (-T7 endonuclease) and debranched (+T7 endonuclease) circular DNA preparations, indicating the high molecular weight molecules (left panel). Resistance of bulk circular DNA preparation to restriction enzymes that efficiently cleave mitochondrial DNA, *Pme*I and *Pac*I, affirming the presence of non-mitochondrial DNA in the samples. M denotes the lanes containing the molecular markers and DNA size in kb is indicated to the left of the gel images. **ii** Mitochondrial (ChrM) content was more challenging to eliminate from Untransformed (U) than crisis samples, plausibly owing to a skew in the ratio of ChrM:eccDNA. Relative proportions of residual mitochondrial DNA within Untransformed (U; 6 replicas), Early (9 replicas) and Deep (3 replicas) crisis stage Circle-Seq datasets revealed as the proportions of structural variant (SV) calls aligned to human Chromosome M (ChrM) normalised to total read outputs. Comparisons employed unpaired non-parametric Mann-Whitney U-tests. **iii** Elevated abundance of eccDNA with progression to Deep crisis. Total structural variant (SV) calls normalised to total Circle-Seq read outputs for the same samples in **ii**. Unpaired non-parametric Mann-Whitney U-tests were utilised to evaluate the differences. **iv** Comparisons of structural variant (SV) calls in Circle-Seq experiments employing eccDNA (circular) or standard phenol-chloroform genomic DNA (linear) samples as library inputs to authenticate eccDNA (and not linear genomic) amplification and sequencing. Data are plotted as SV reads normalised to total reads and analysed using unpaired non-parametric Mann-Whitney U-tests. **C** Inverted PCR validation of sequenced eccDNA junctions. Integrative Genomics Viewer displays of eccDNA incorporating *RUNX1* (left) and *WWOX* (right) coding sequences resolved by SV mapping of Circle-Seq data in Early and Deep crisis MRC5^{E6E7} cells. Flanking soft-clipped reads marking the eccDNA junctions were verified by PCR and Sanger sequencing, as shown below. M denotes the molecular weight markers in kb. Expected eccDNA junction amplicon size is indicated adjacent to the images. PCR was performed using circular DNA (+), linear

genomic DNA (-) or water (H_2O) as input material. The eccDNA junction sequences and T2T CHM13v2.0/hs1 reference locations are noted.

Supplemental Figure S4

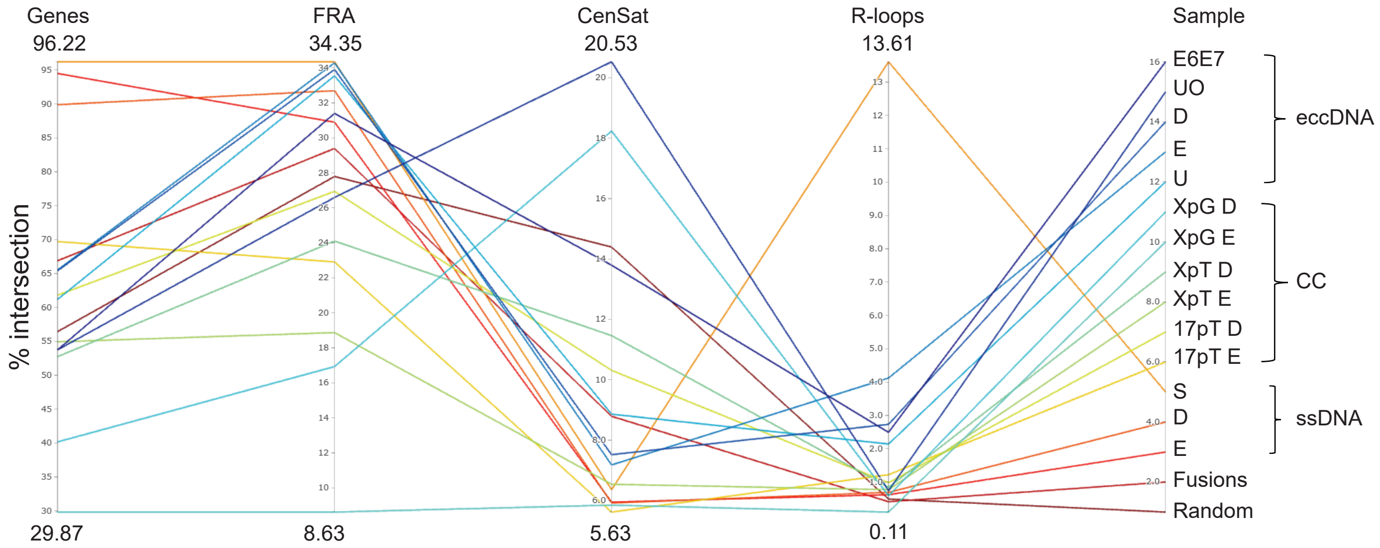

Supplemental Figure S4: Features of MRC5 multiomics datasets generated in this study

A parallel plot displaying the proportions of genomic intervals (defined by 'BEDTools intersect' (Quinlan and Hall 2010) intersecting with specified genomic features for key datasets analysed in this study; Genes, coding sequence; FRA, fragile sites; CenSat, centromere satellites; R-loops, 3-stranded nucleic acid structures. Samples include; extra-chromosomal circular DNA (eccDNA) from Untransformed (U) and MRC5^{E6E7} cells at Early (E) and Deep (D) time points, as well as eccDNA detected exclusively in MRC5^{E6E7} (E6E7) or Untransformed cells (UO). Chromatin capture (CC) datasets were collected at Early (E) and Deep (D) time points using hybridisation probes targeting the Chr17p and ChrXpYp telomere-adjacent sequence (17pT; XpT) or a non-coding ChrXp genomic locus (XpG). Single-strand DNA (ssDNA) peaks determined by KAS-seq in Early (E) or Deep (D) crisis samples or common to both datasets (Shared; S) are also displayed, alongside telomere fusions sequenced from MRC5^{E6E7} crisis cells and a simulated dataset of 1 million random genomic loci (Random). The upper and lower boundaries of the proportions are annotated at the top and bottom of the chart, respectively.

Supplemental Figure S5

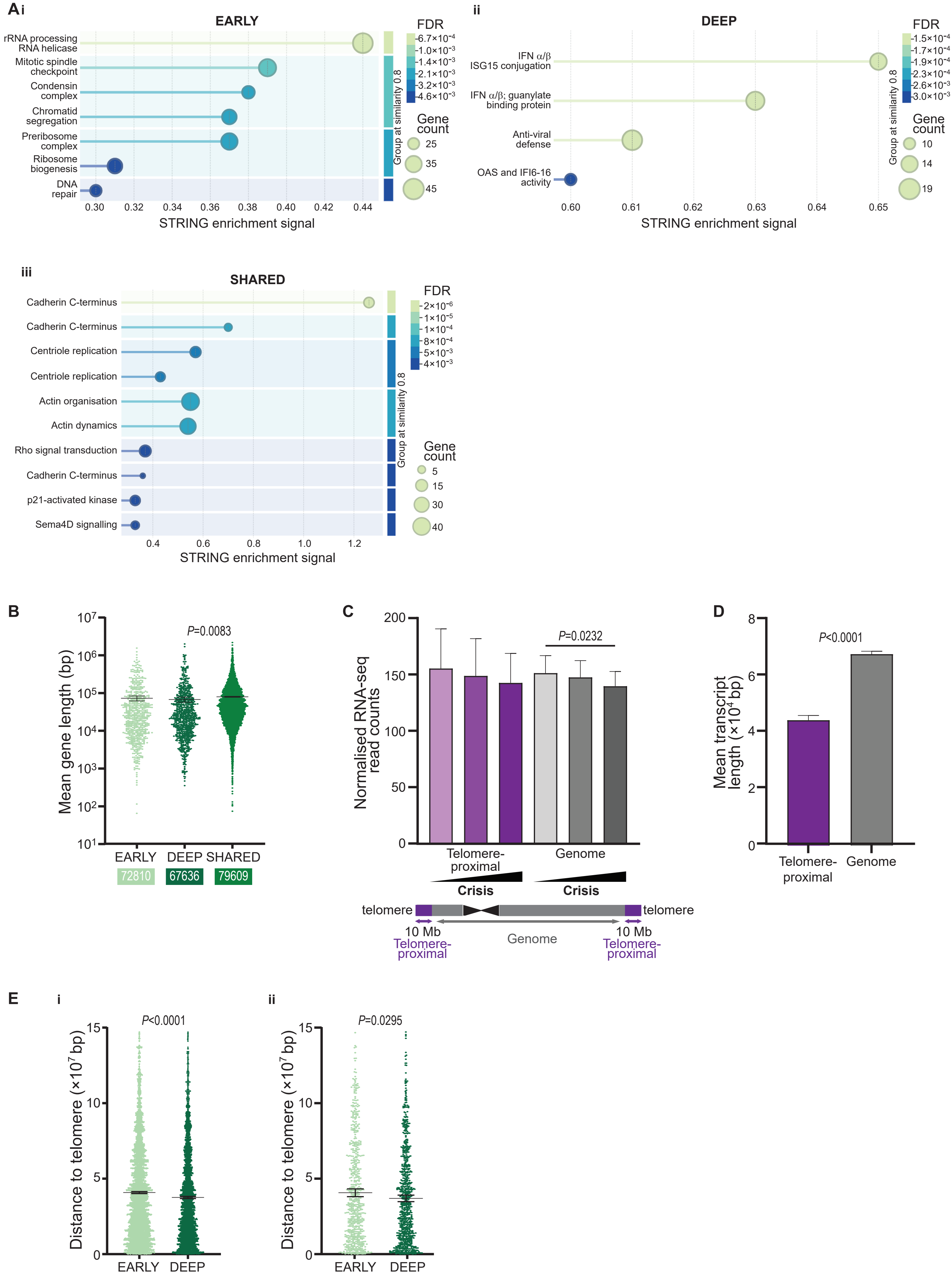

Supplemental Figure S5: Distributions, features and functions of genes enriched in MRC5^{E6E7} crisis datasets

A Genes associated with ssDNA signal peaks in **i** Early, **ii** Deep or **iii** both crisis stages ('Shared') determined by KAS-seq of MRC5^{E6E7} crisis cells were investigated for functional interactions and enrichments using STRING (Szklarczyk et al. 2023). STRING protein functional enrichment (using local network clustering) profiles of the **i** Early, **ii** Deep or **iii** 'Shared' MRC5^{E6E7} crisis datasets are displayed as lollipop plots with rows merged by term similarity $\geq$ 0.9. Enriched biological terms are noted to the left of the panels. Circle size represents group gene count and the background plot colour indicates the False Discovery Rate (FDR) according to the key in the top right. The STRING enrichment signal (X-axis) is a composite score that balances enrichment magnitude (observed/expected) and statistical significance (FDR). **B** The lengths of genes (means annotated below the X-axis in base pairs, bp) exclusive to Early or Deep crisis KAS-seq ssDNA datasets or Shared by both are displayed and analysed using unpaired parametric *t*-tests. **C** Mean expression (RNA-seq) of telomere-proximal (purple) and more distant genes (grey) in MRC5^{E6E7} cells (progression of replicative crisis indicated by the black triangle) are presented as means with 95% CI for the 3 crisis time points described in (Liddiard et al. 2021). Paired parametric *t*-tests were used to test whether the differences between crisis time points for the same data series were statistically significant. **D** The lengths in base pairs (bp) of all gene transcripts within 10 Mb of each telomere (telomere-proximal) or further away (Genome) are displayed as means with 95% CI. Statistical analyses utilised unpaired parametric *t*-tests with Welch's correction for unequal Standard Deviation (SD). **E** The distances (bp) of all unique Early (3 replicas) and Deep (2 replicas) crisis **i** ssDNA signal peaks or **ii** genes coincident with these to the telomere on the same chromosome arm are plotted with means and 95% CI. Statistical evaluation was by unpaired parametric *t*-test.

Supplemental Figure S6

A

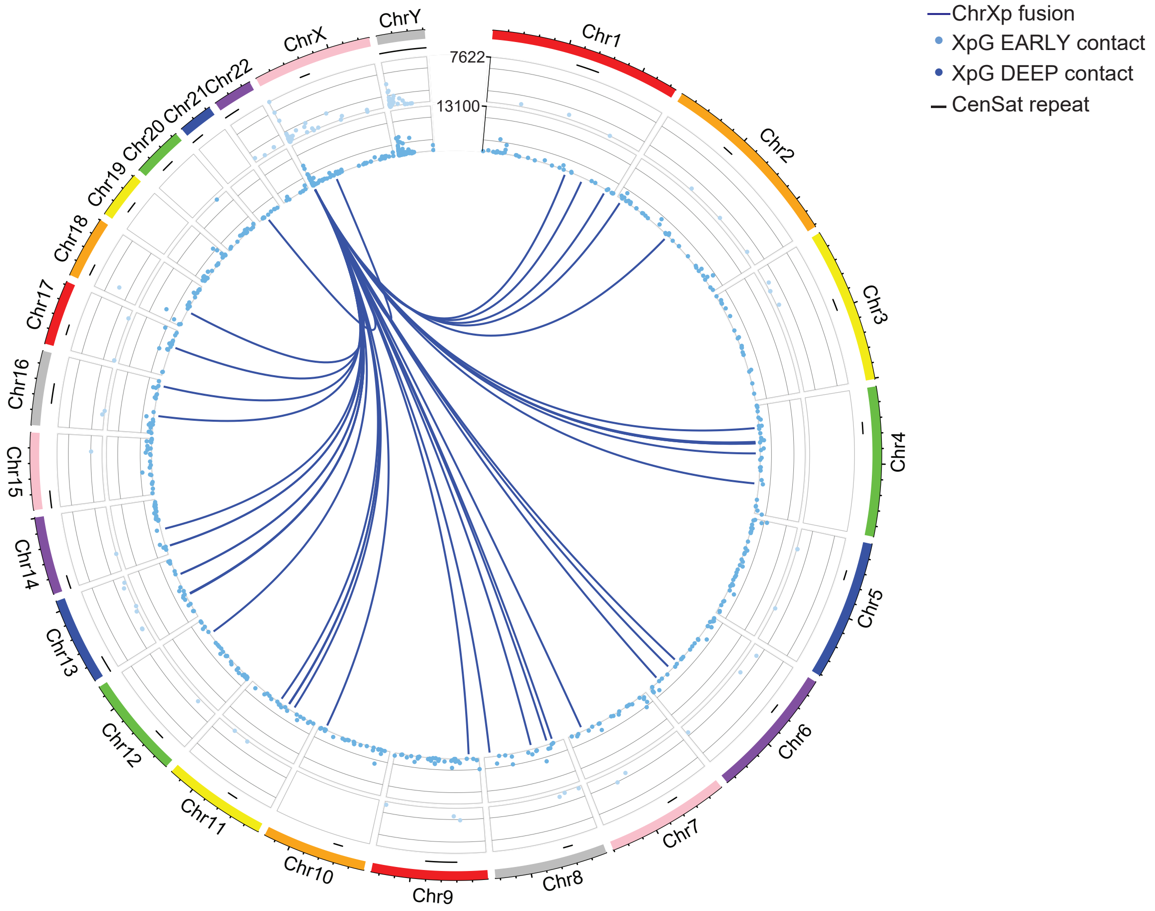

B

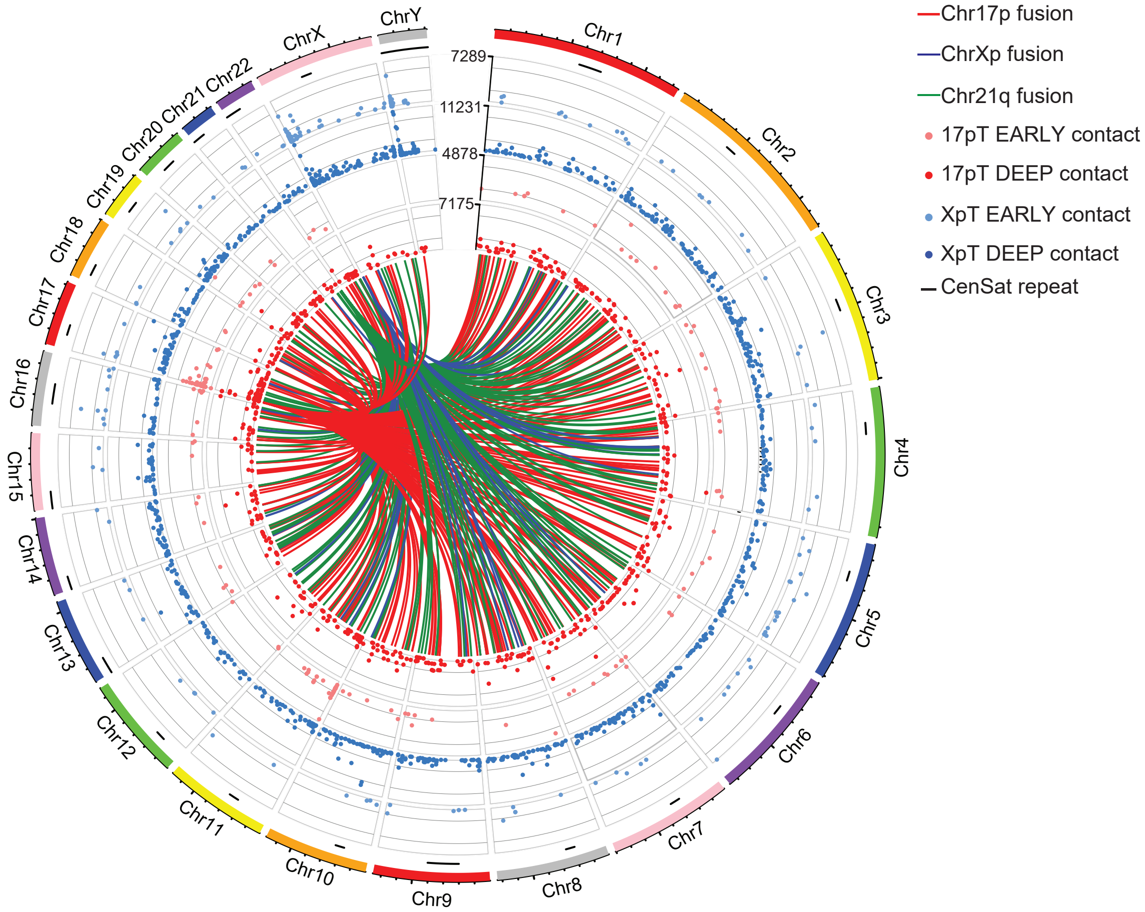

Supplemental Figure S6

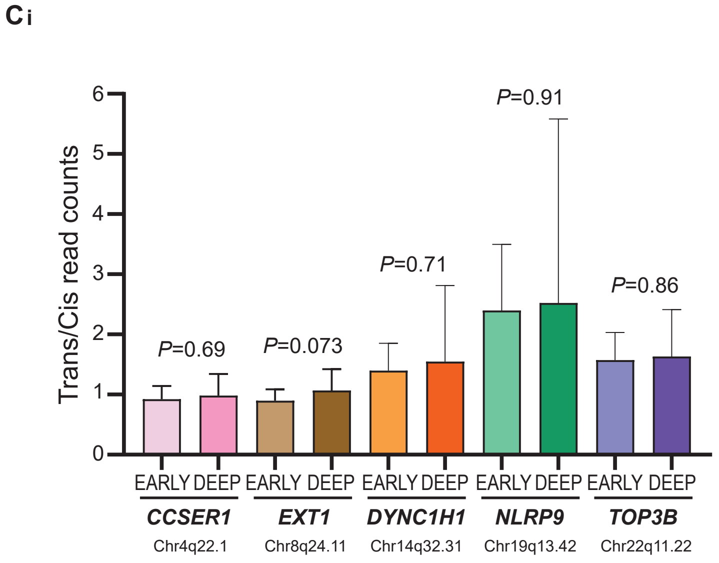

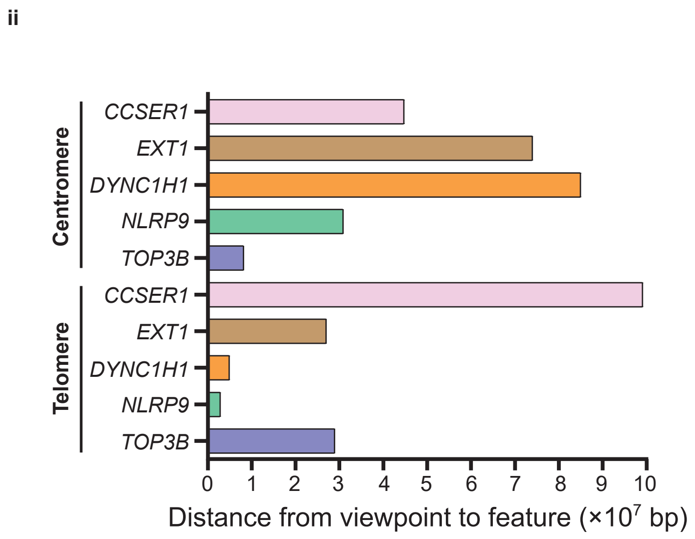

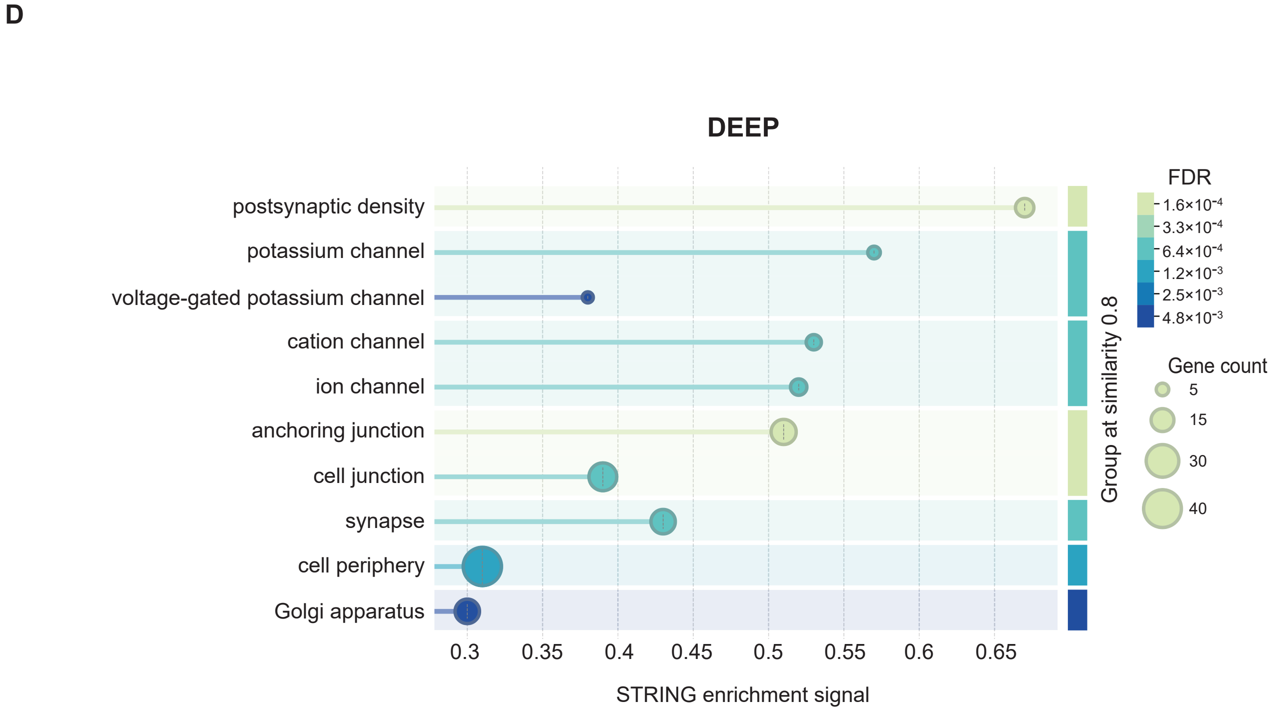

Supplemental Figure S6: Functional overlaps between telomere contacts and telomere fusions

A Circos plot depicting all MRC5^{E6E7} Early and Deep crisis chromatin interactions with the ChrX internal control (XpG) probe determined using Capture-C as concentric tracks. Track height indicates signal intensity. Fusions of the ChrXp telomeres (blue links) with genomic sites span the circle centre. Peripheral black lines underneath the chromosome identities demarcate the centromere and pericentromeric satellite (CenSat) repeats. **B** Circos plot depicting all MRC5^{E6E7} Early and Deep crisis chromatin interactions sequenced following Capture-C with telomere-adjacent (17pT, red; XpT, blue) probes as concentric tracks. Track height indicates signal intensity. Telomere fusions with genomic loci span the circle centre (Chr17p fusions, red; Chr21q fusions, green; ChrXp fusions, blue). Black lines underneath the chromosome identities demarcate the CenSat repeats. **Ci** Normalised read counts for all long-range trans compared with local cis chromatin Capture-C interactions for the coding sequence control viewpoints indicated (*CCSER1, EXT1, DYNC1H1, NLRP9* and *TOP3B*), as determined by the Capcruncher pipeline. Genomic locations of the viewpoints are noted below the gene names. Triplicate samples of the Early and Deep crisis MRC5^{E6E7} sampling points using the each probe set are displayed as means with 95% confidence intervals (CI), analysed using paired parametric *t*-tests. **ii** The distance in base pairs (bp) between the Capture-C coding sequence control viewpoints indicated and the centromere (upper) or telomere (lower) on the same chromosome arm. **D** STRING (Szklarczyk et al. 2023) protein functional enrichment profiles of genes overlapping between telomere fusion and telomere-adjacent probe (17pT, XpT) Capture-C datasets derived from Deep crisis MRC5^{E6E7} cells. Enrichments in cellular components are displayed, with the biological terms noted to the left of the panel and rows merged by term similarity $\geq$ 0.9. Circle size represents group gene count and the background plot colour indicates the False Discovery Rate (FDR) according to the key in the top right. The STRING enrichment signal (X-axis) is a composite score that balances enrichment magnitude (observed/expected) and statistical significance (FDR).

Supplemental Figure S7

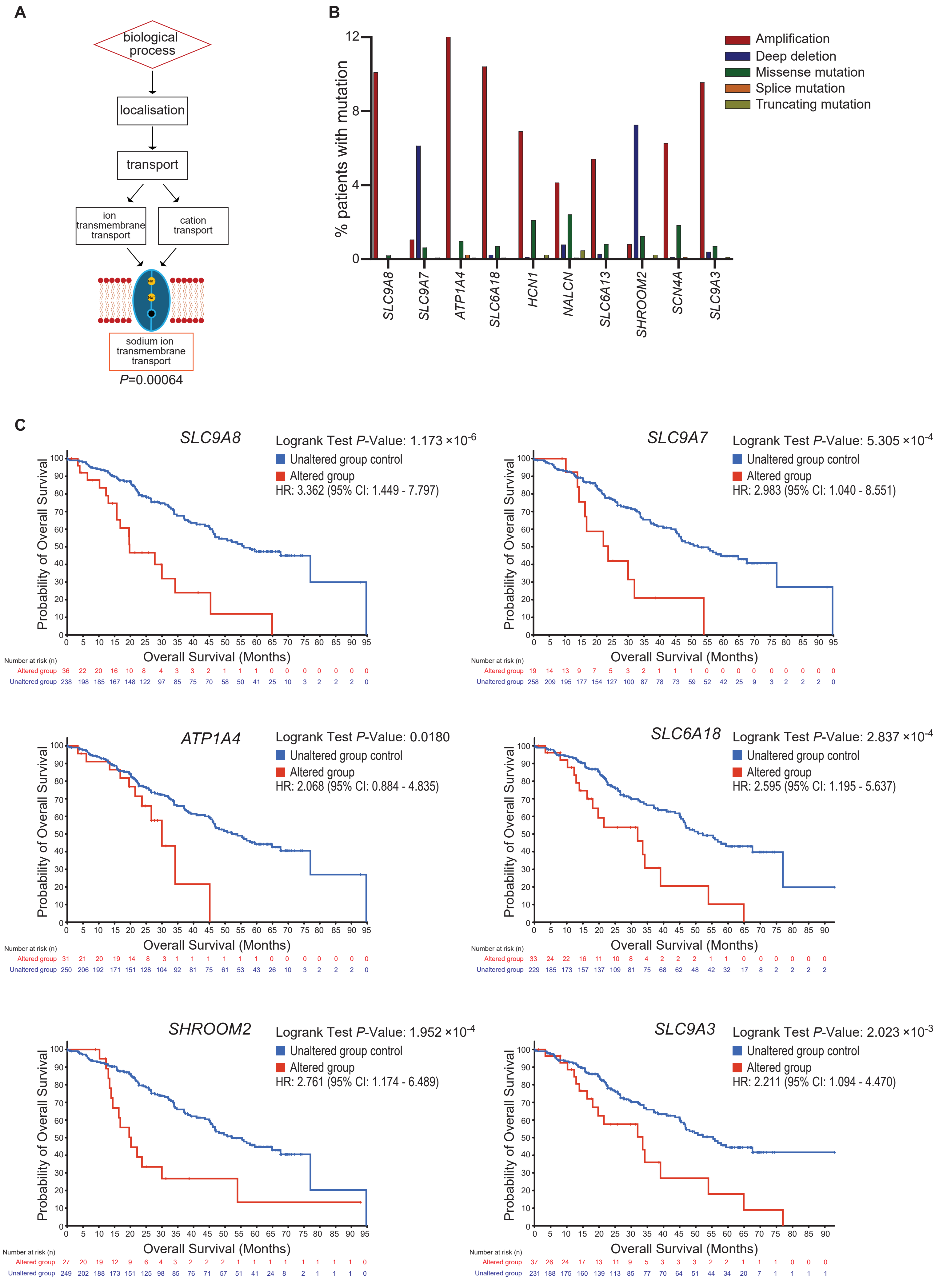

Supplemental Figure S7: Impact of disruptions to crisis gene circuits for cancer patient survival

A Over-representation of genes within cation transport ontology groups (P-value indicated) for the Deep crisis compared with Early crisis Capture-C telomere-adjacent probe datasets identified using GOrilla comparative analyses (Eden et al. 2009). **B** The cBioPortal TCGA Pan-cancer database (ICGC/TCGA Pan-Cancer Analysis of Whole Genomes Consortium 2020) of 2565 patients was interrogated for mutations in the 40 sodium ion channel genes comprising the enriched pathway indicated in **A**. These genes are altered in 70% queried patients (2583 patients total) and 66% queried samples (2922 samples total). The proportions of patients with various types of mutations (indicated in the key) in 10 genes with mutation prevalence exceeding 10% are displayed. **C** Six genes conferred a significantly reduced survival rate for the same patient dataset when sequence or expression was affected. Kaplan-Meier curves disclosing the differential overall survival (months) in patients with (red) or without (blue) alterations in each of these genes are shown with the associated hazard ratios (HR) and 95% CI.

Supplemental Figure S8

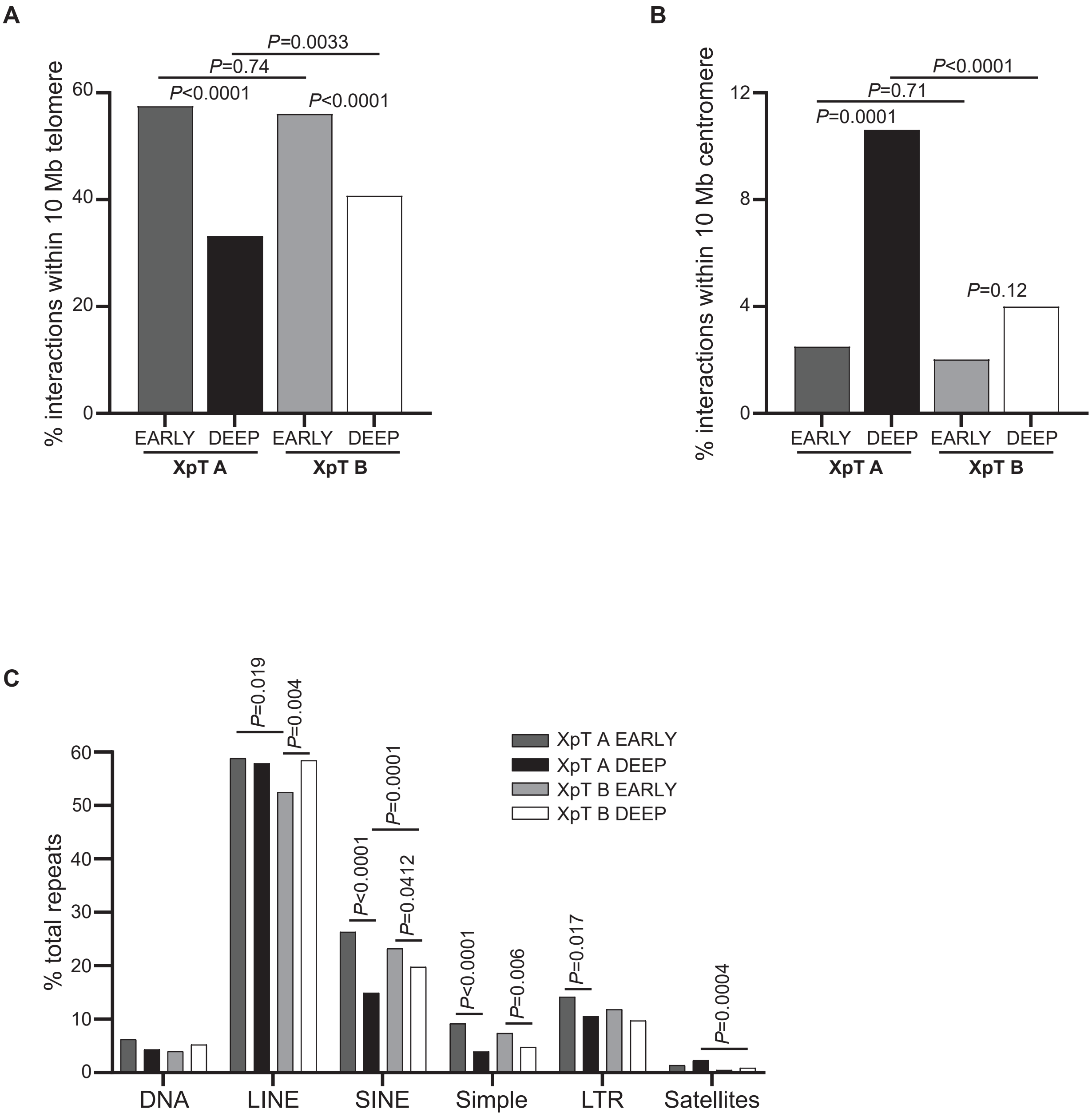

Supplemental Figure S8: Distinct genomic interactions of long and short MRC5 ChrXp telomere alleles during crisis progression

XpT telomere-adjacent Capture-C probe interactions were categorised according to the presence or absence of single nucleotide polymorphisms (SNP) characteristic of the long (A) or short (B) ChrXp telomere allele. **A** The proportions of A and B allelic interactions detected within 10 Mb of telomere sequences during Early and Deep crisis phases are plotted in a bar chart and compared using the N-1 Chi-square method. **B** The proportions of A and B allelic interactions detected within 10 Mb of telomere sequences during Early and Deep crisis phases are presented and analysed using the N-1 Chi-square method. **C** The proportions of genomic intervals of A and B allelic interactions at Early and Deep crisis stages that intersect with distinct repeat classes are displayed as percentages of the total intersections with DNA repeats and evaluated using the N-1 Chi-square method. LINE, long interspersed repeat; SINE, short interspersed repeat; LTR, long terminal repeat.

Supplemental Figure S9

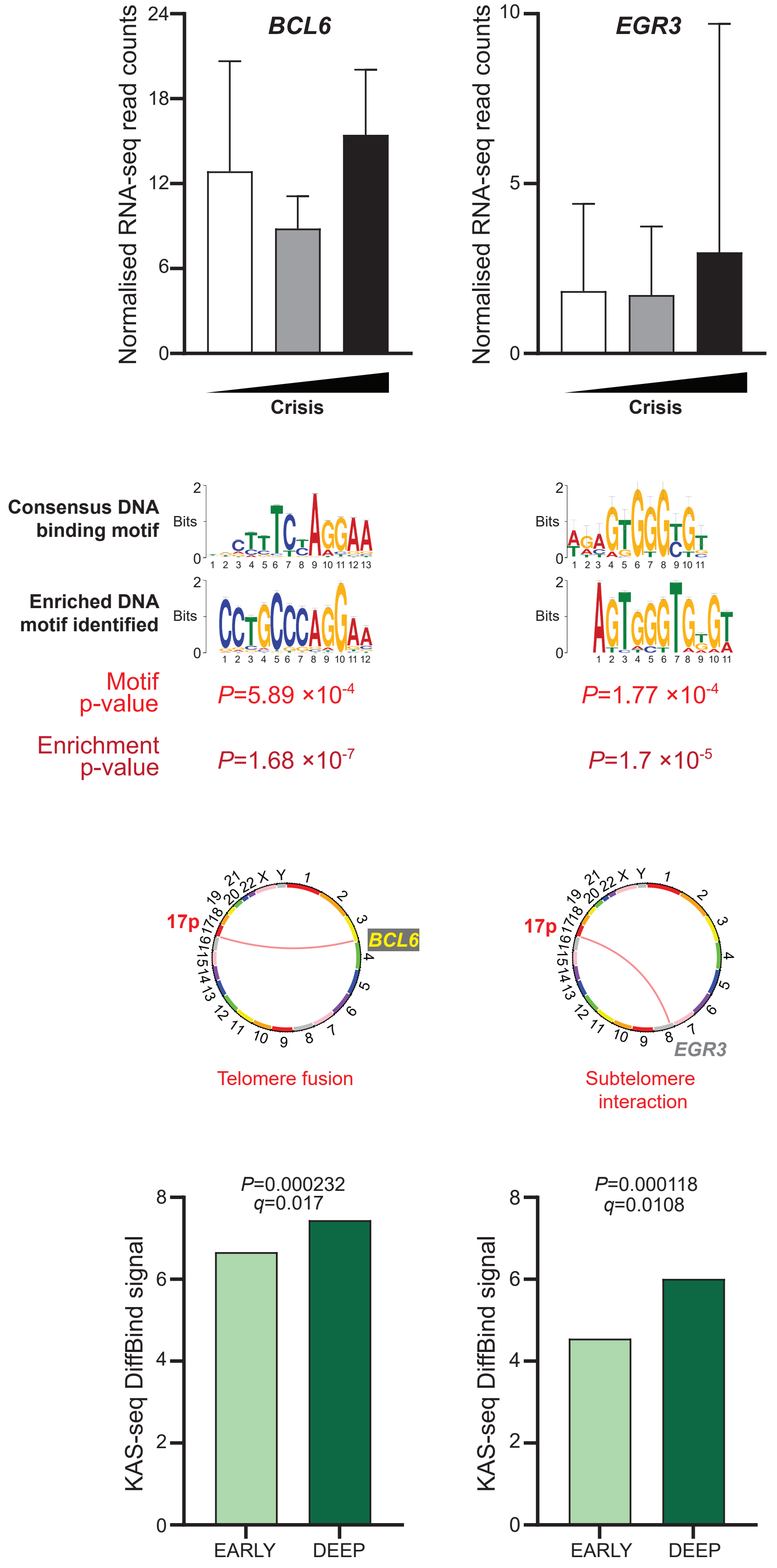

Supplemental Figure S9: DNA motifs that underpin telomere interactions with the genome

DNA motifs (HOCOMOCO human v11 FULL database) enriched within 5 kb up- and downstream of genomic sites contacting and fusing with telomere-adjacent sequences in MRC5^{E6E7} crisis cells were identified using the MEME suite (version 5.5.5) XSTREME tool. BCL6 and EGR3 putative binding sites were the top-ranking DNA motifs enriched only within telomere-adjacent sequence interactions (17pT and XpT probes). The upper bar charts display the normalised RNA-seq reads (means with 95% CI) for *BCL6* and *EGR3* re-analysed from our former study using HPV16 *E6E7*-transformed MRC5, HCA2, IMR90 and WI38 human fibroblasts sampled at progressive time points in replicative crisis (denoted with black triangle) (Liddiard et al. 2021). The central panels reveal the DNA motif logos for BCL6 and EGR3 derived from the Tomtom motif comparison tool. The upper logo details the consensus sequence for the binding site (target) and the lower logo represents the motif enriched within the input sequences (queries). The logo X-axis indicates the position along the motif, while the Y-axis shows information content (bits). At each position, the total stack height reflects sequence conservation and the relative heights of the letters represent the nucleotide frequencies at that position. The Tomtom motif P-values (upper), as well as the Simple Enrichment Analysis (SEA) P-values (lower) are given. The Circos plots below portray a Chr17p telomere fusion (left) and a long-range Chr17p subtelomere interaction (right) with *BCL6* and *EGR3* genes, respectively, identified in our MRC5^{E6E7} crisis datasets. The lower bar charts represent ssDNA signal peaks coincident with these genes, as determined by DiffBind (Stark and Brown 2011) analysis of KAS-seq outputs from Early and Deep crisis MRC5^{E6E7} cells, with the associated statistical evaluations (*P* and *q*-values) displayed above.

Supplemental Figure S10

A

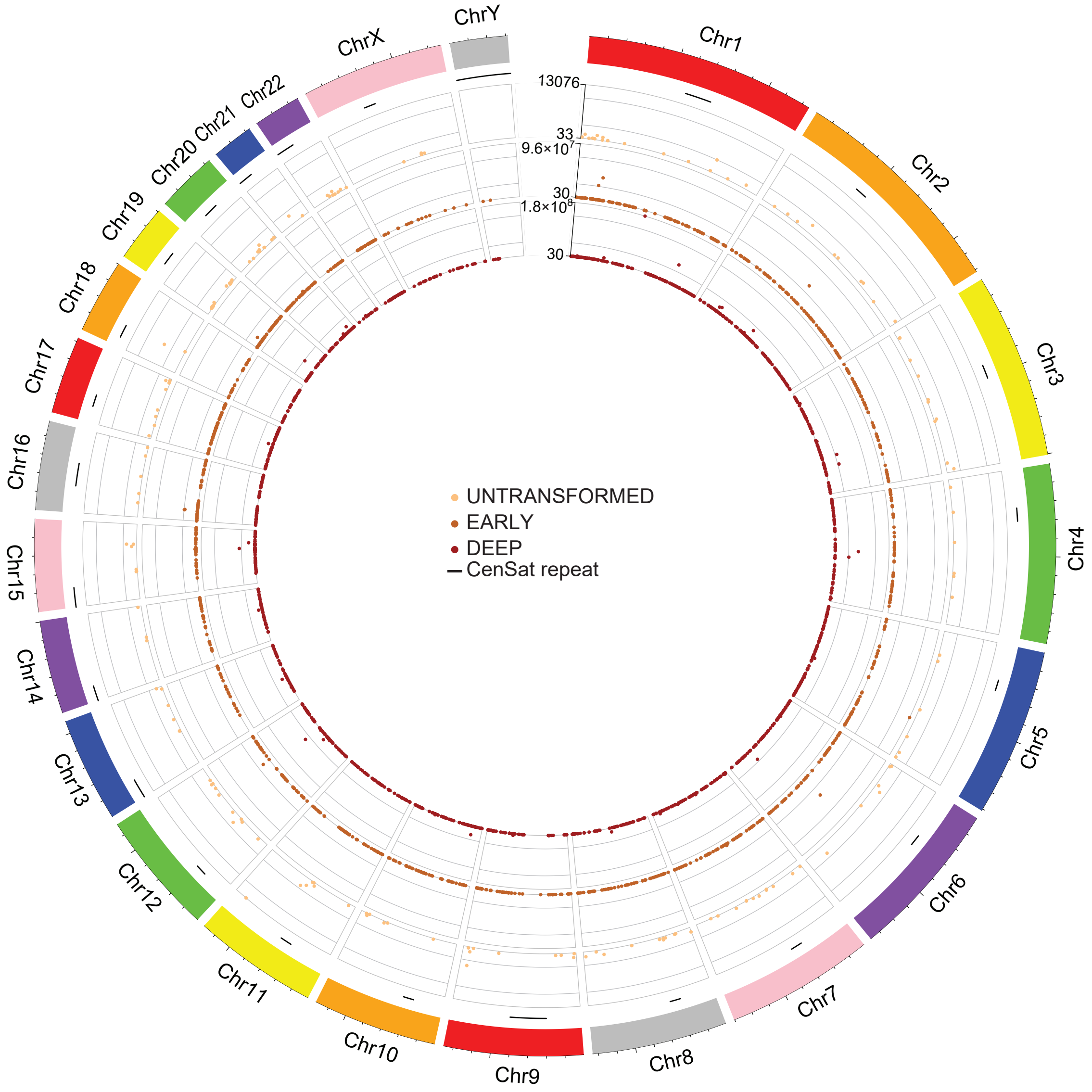

B

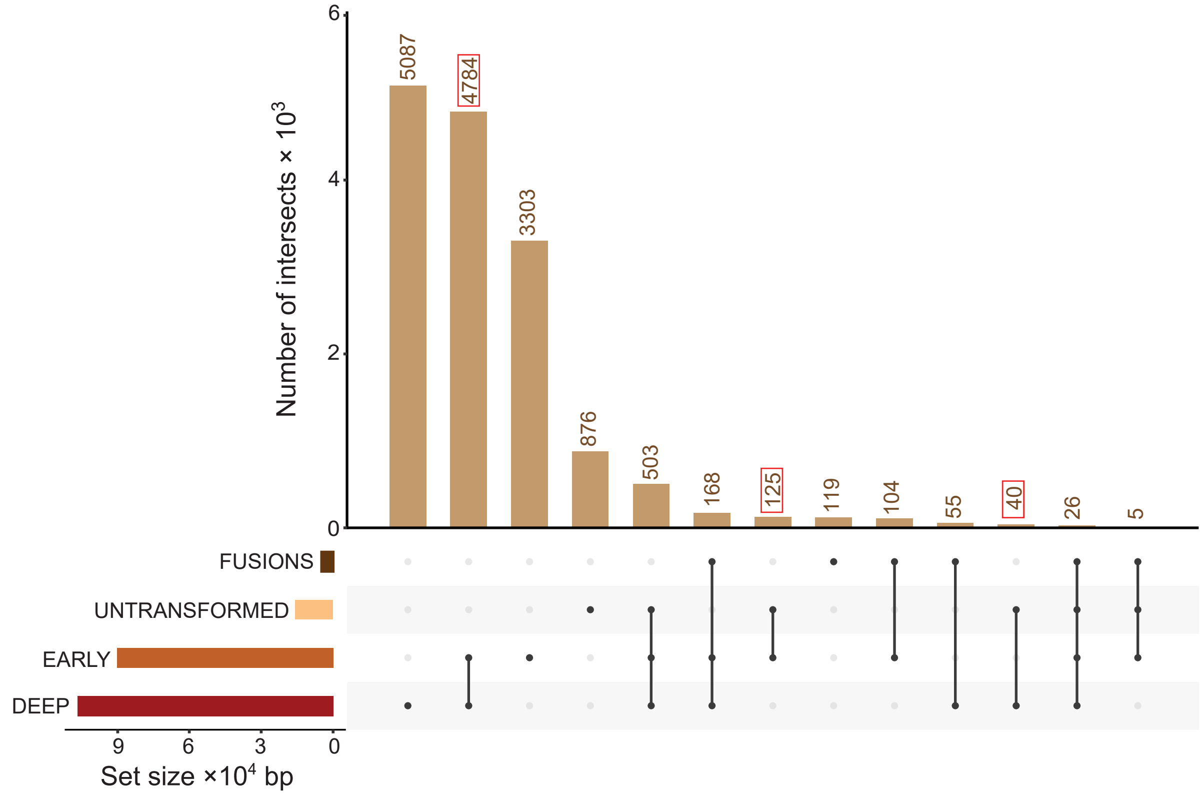

Supplemental Figure S10: Genomic distributions of extra-chromosomal DNA circles from Untransformed MRC5 and $MRC5^{E6E7}$ undergoing replicative crisis

A Circos plot depiction of the genomic positions of all eccDNA sequenced from Untransformed MRC5 (U; 6 replicas) and Early (E; 9 replicas) and Deep (D; 6 replicas) crisis $MRC5^{E6E7}$ presented in **Figure 5A** displayed as concentric tracks. Track height indicates signal intensity and the positions of the centromeres are indicated with black peripheral lines marking the CenSat repeats. **B** UpSet plot illustrating intersecting genomic intervals shared by these eccDNA datasets and telomere fusions sequenced from $MRC5^{E6E7}$ crisis cells. Vertical lines connect the overlapping datasets, with vertical bars showing the number of elements in each intersection, enumerated above the bars. Key intersections are boxed in red. The dataset (Set) sizes in nucleotide base pairs (bp) are depicted as horizontal bars, lower left.

Supplemental Figure S11

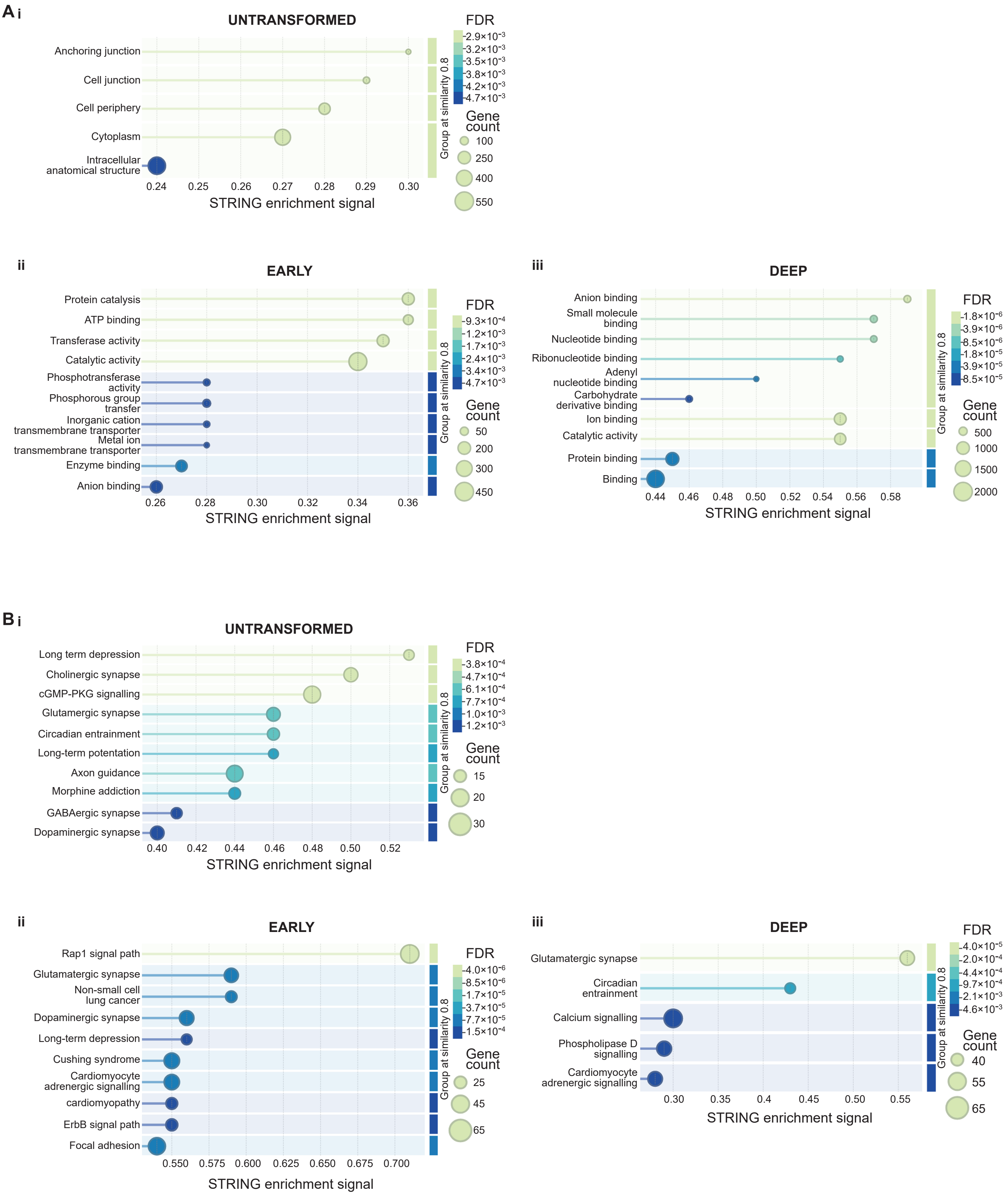

Supplemental Figure S11: eccDNA bear genes enriched in ion transport and synaptic signalling

STRING (Szklarczyk et al. 2023) protein functional enrichment lollipop plots of **A** genes exclusively detected in **i** Untransformed and **ii** Early and **iii** Deep crisis MRC5 cells as defined by the Venn diagram in **Figure 5Di**. Since the datasets do not share a common category of functional enrichment, 'cellular components' are displayed for the Untransformed MRC5 eccDNA genes, whereas 'molecular functions' are shown for the Early and Deep crisis eccDNA genes. **B** Functional enrichments in KEGG (Kyoto Encyclopedia of Genes and Genomes) pathways are displayed for genes associated with genomic intervals defined for the same **i** Untransformed, **ii** Early and **iii** Deep crisis eccDNA datasets using BEDTools (Quinlan and Hall 2010). For both **A** and **B**, enriched biological terms are noted to the left of the panels and rows were merged by term similarity $\geq$ 0.9. Circle size represents group gene count and the background plot colour indicates the False Discovery Rate (FDR) according to the key in the top right. The STRING enrichment signal (X-axis) is a composite score that balances enrichment magnitude (observed/expected) and statistical significance (FDR).

Supplemental Figure S12

A

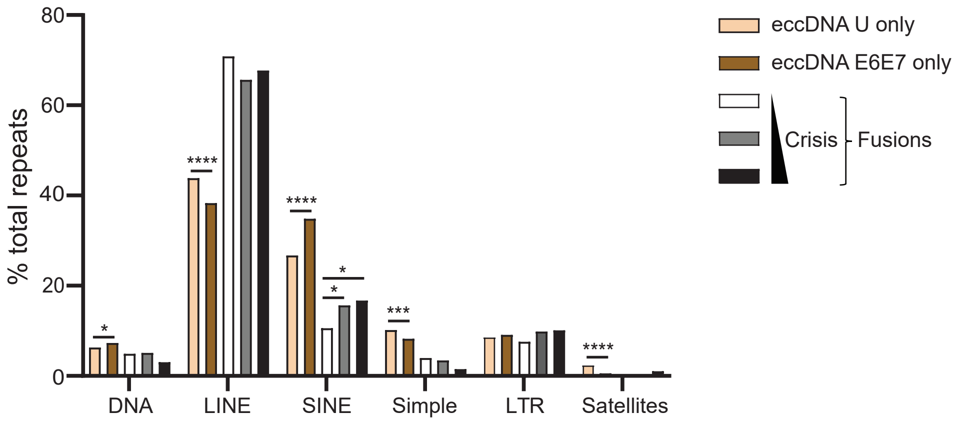

B

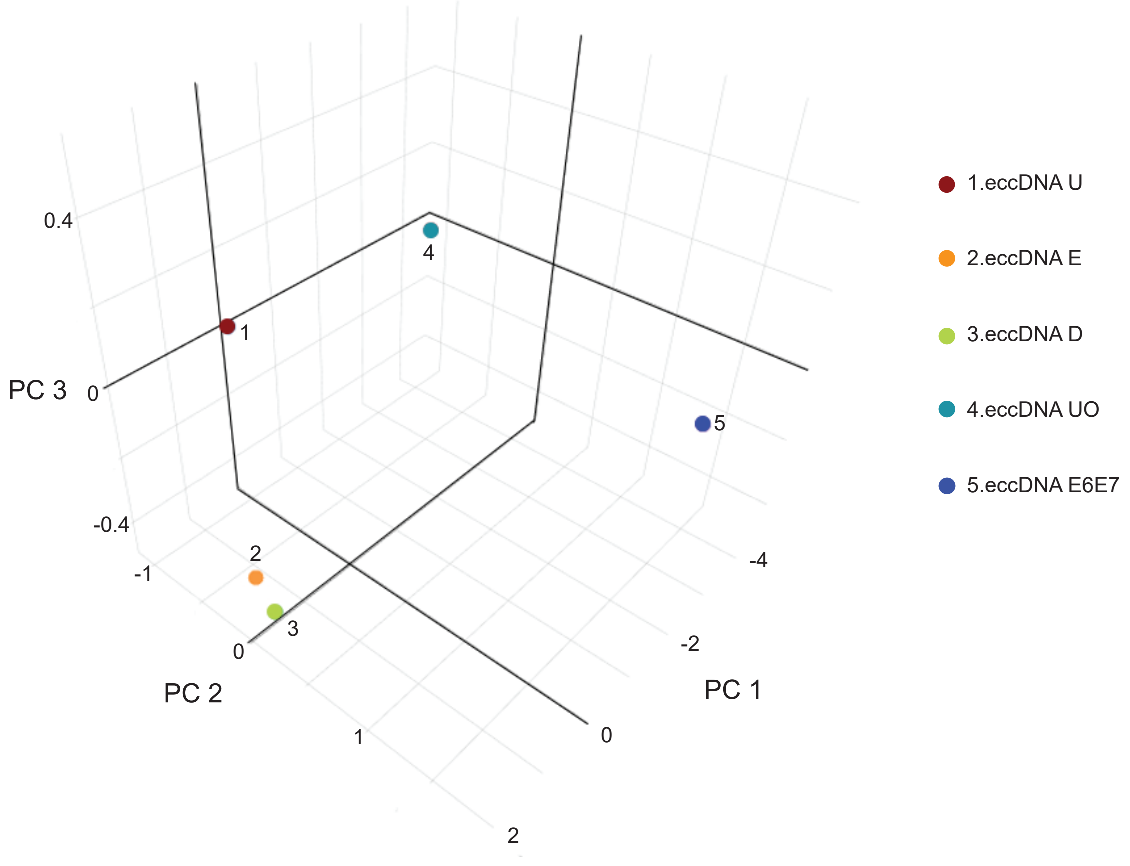

Parameter	PC1 (86.2%)	PC2 (11.9%)	PC3 (1.8%)
% DNA	-0.377	-0.127	-0.167
% LINE	0.376	0.114	0.296
% SINE	-0.345	0.376	-0.548
% Simple	-0.334	-0.486	-0.164
% LTR	-0.364	-0.204	0.567
% Retroposon	0.325	-0.531	-0.044
% Low-complexity	-0.362	-0.318	0.099
% Satellite	0.342	-0.41	-0.474

Supplemental Figure S12: Divergence of eccDNA exclusive to Untransformed or crisis samples

A Bar chart displaying the differential associations of eccDNA exclusive to Untransformed or E6E7-transformed MRC5 or telomere fusions sequenced from progressive $MRC5^{E6E7}$ crisis stages (denoted by the black triangle and darker greyscale) and expressed as a proportion of the total repeats associations for each sample. Statistical evaluation employed the N-1 Chi-square method, with results displayed as: * P<0.05; ** P<0.01; *** P<0.001; **** P<0.0001. **B** A Principle Component Analysis (PCA) plot (Galaxy Community 2024) of the eccDNA datasets detailed in the key,

based on associations with DNA, LINE, SINE, simple, LTR, retroposon, low-complexity and satellite DNA repeats (characterised by RepeatMasker (Smit et al. 2013)). The table below displays the parameter contributions to the variance driving sample clustering. U, Untransformed MRC5; E, Early crisis $MRC5^{E6E7}$; D, Deep crisis $MRC5^{E6E7}$; UO, Untransformed-only (exclusive); E6E7, transformed-only (exclusive).

Supplemental Figure S13

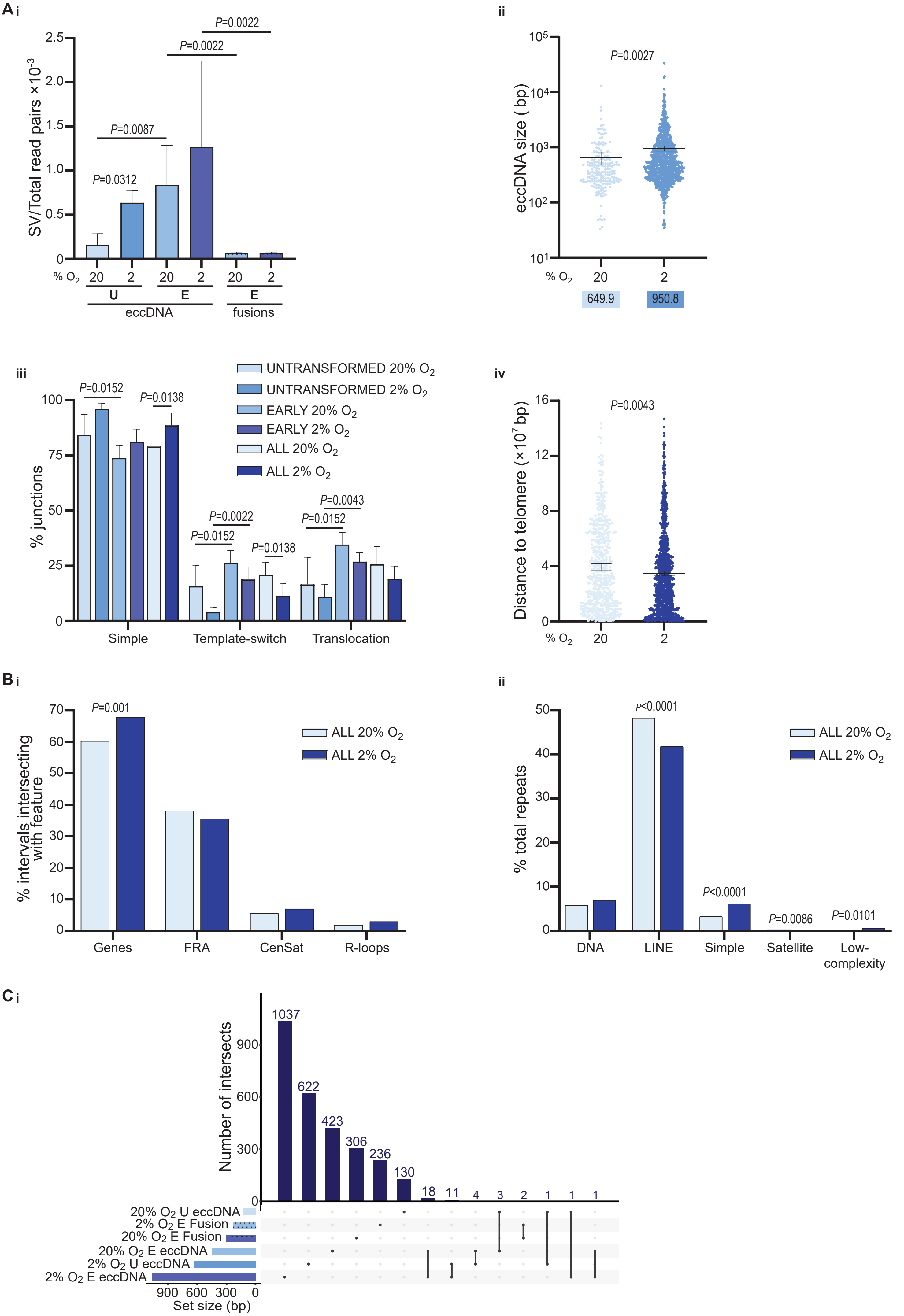

Supplemental Figure S13

C ii

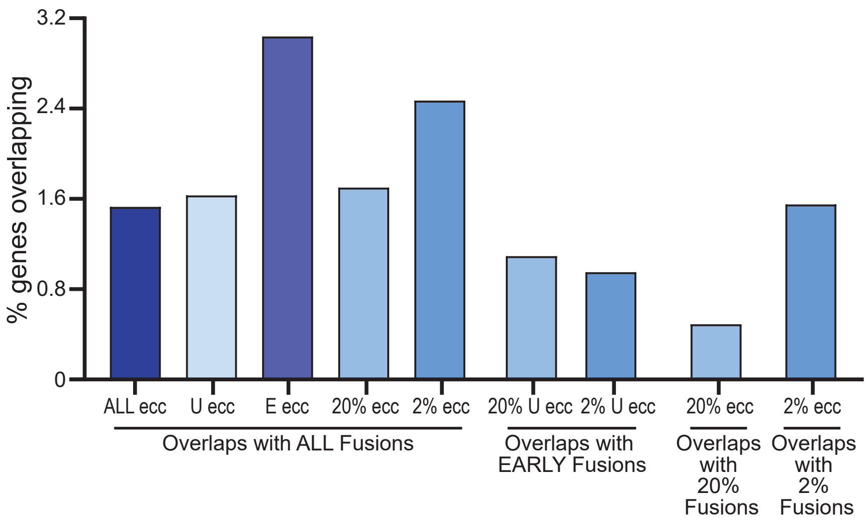

D

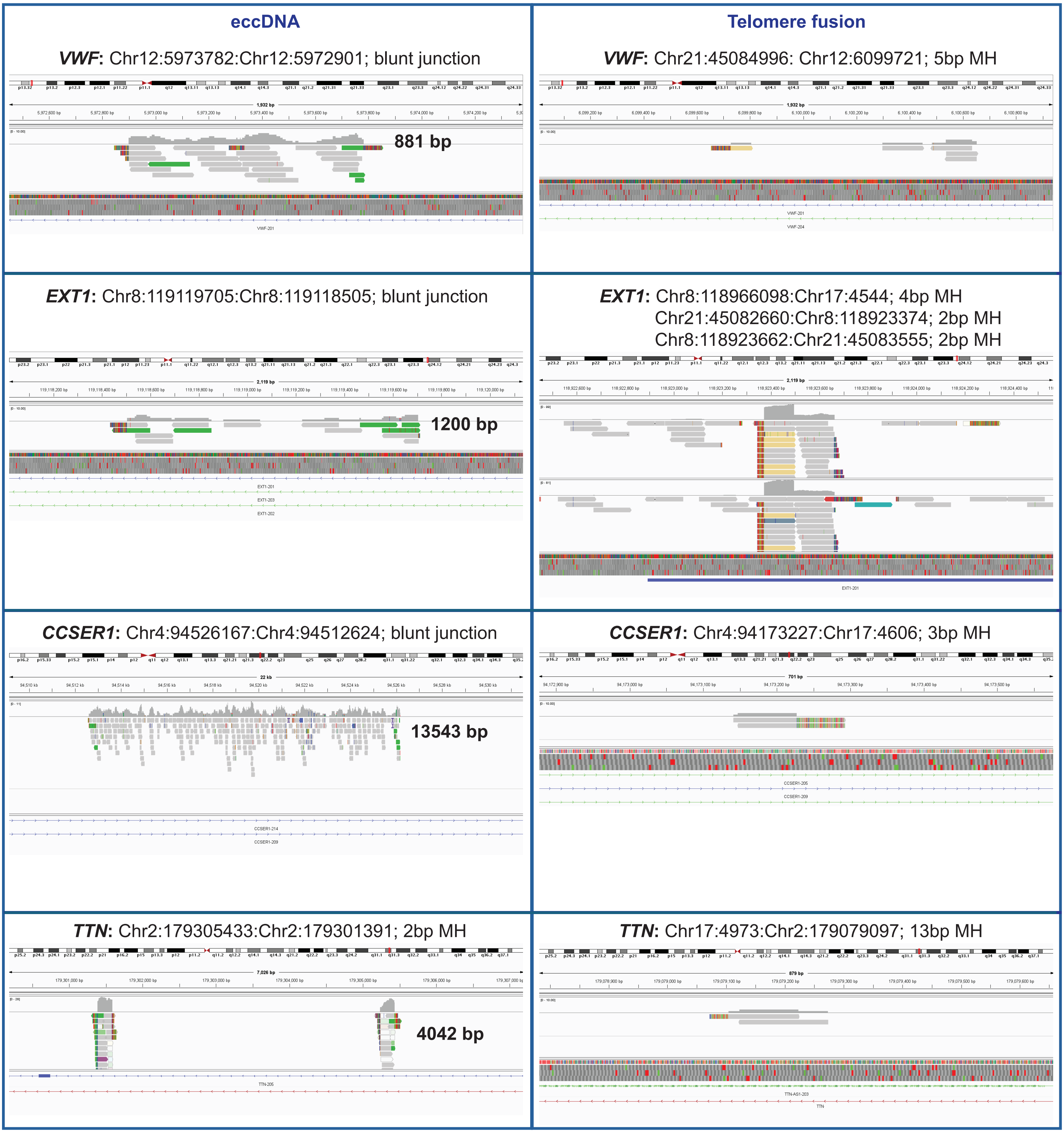

Supplemental Figure S13: eccDNA profiles reflect environmental stressors

Ai The numbers of structural variants (SV) normalised to total sequenced read pairs are presented for 3 replicas of each Untransformed (U) and Early crisis (E) eccDNA and telomere fusion samples prepared from cells exposed to low (2%) or standard tissue culture (20%) oxygen (O_2) for 24 hr. Means with 95% CI are displayed. Wilcoxon matched-pairs signed rank non-parametric tests were used to compare 2% with 20% O_2 samples; Mann-Whitney unpaired non-parametric U-tests were used to compare Untransformed with Early crisis and eccDNA with fusion datasets. **ii** A $\log_{10}$ scale scatter plot depicting the mean sizes with 95% CI of all eccDNA amplified from Untransformed cells cultured in 20% or 2% oxygen O_2 for 24 hr (means annotated below the X-axis; bp, base pairs). Statistical comparison was by unpaired parametric *t*-test with Welch's correction for unequal SD. **iii** The proportions of eccDNA junctions amplified from Untransformed and Early crisis 2% and 20% O_2-treated cells that comprise DNA segments derived from the same strand ('Simple') or opposing strands ('Template-switch) or distant chromosome locations ('Translocation') are depicted as a bar chart of means with 95% CI. Wilcoxon matched-pairs signed rank non-parametric tests were used to compare 2% with 20% O_2 samples; Mann-Whitney unpaired non-parametric U-tests were used to compare Untransformed with Early crisis samples and crisis-stage agnostic ('ALL') samples. **iv** The distances of all genes captured in eccDNA and telomere fusions amplified from cells cultured in 2% or 20% O_2 for 24 hr to the telomere on the same chromosome arm are illustrated in a scatter plot with means and 95% CI and compared using Mann-Whitney unpaired non-parametric U-tests. **Bi** The proportions of all eccDNA derived from cells cultured in 2% or 20% O_2 for 24 hr that intersect with Genes, Fragile sites (FRA), peri/centromeric satellite repeats (CenSat) and R-loops, Expressed genes and CenSat repeats are presented in a bar chart with differences compared using the N-1 Chi-square method. **ii** Total repeats intersections determined for the same samples were analysed for specific repeat content (delineated by RepeatMasker (Smit et al. 2013)) using the same methodology. **Ci** An UpSet plot illustrating genomic intervals that overlap in eccDNA datasets and telomere fusions sequenced from $MRC5^{E6E7}$ crisis cells. Vertical lines connect the intersecting datasets; intersection totals are indicated above the bars; nucleotide base pairs comprising each dataset (set size) are indicated by the horizontal bars, lower left. U, Untransformed; E, Early crisis. **ii** The proportions of all genes overlapping between eccDNA (ecc) and telomere fusion (transformed cells only) datasets compiled for Untransformed (U) and Early crisis (E) or all (ALL) MRC5 cells cultured in standard tissue culture (20%) and low (2%) O_2 are displayed as a bar chart and compared using the N-1 Chi-square method. **D** Examples of genes detected in eccDNA and telomere fusions within these datasets are illustrated. The left

panels display the Integrative Genomics Viewer (IGV) displays and junction locations and features for the eccDNA and the right panels portray the corresponding telomere fusions. Microhomology (MH) in base pairs (bp) is indicated where appropriate. Recombinations involving *VWF* and *CCSER1* were detected in eccDNA and fusions amplified from low O_2 samples; *TTN* was captured within eccDNA and fusions amplified from standard O_2 cultures; *EXT1* was identified in eccDNA from Untransformed standard O_2 treated cells and fusions within both low and standard O_2 cultures.

Supplemental Figure S14

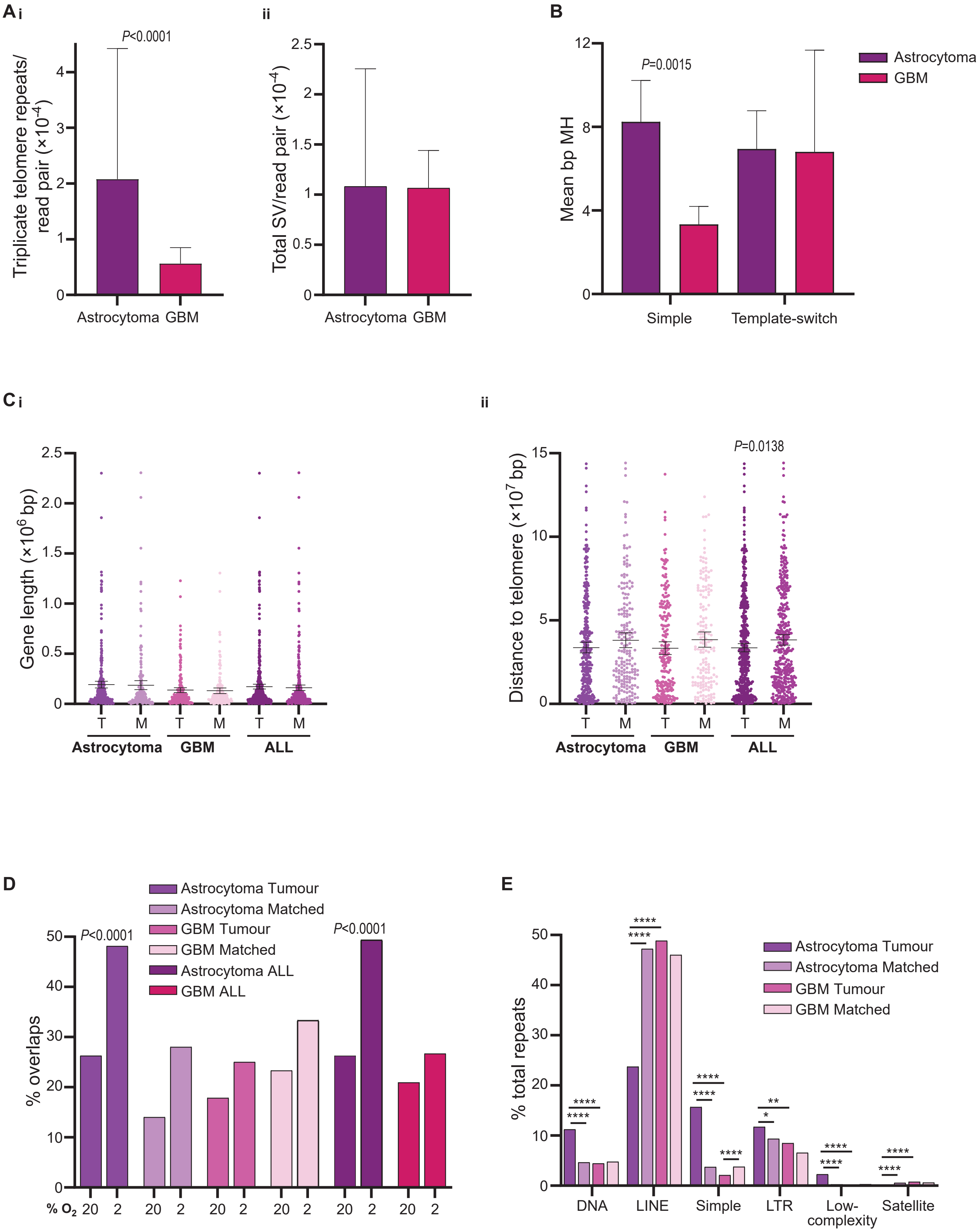

Supplemental Figure S14

Fi

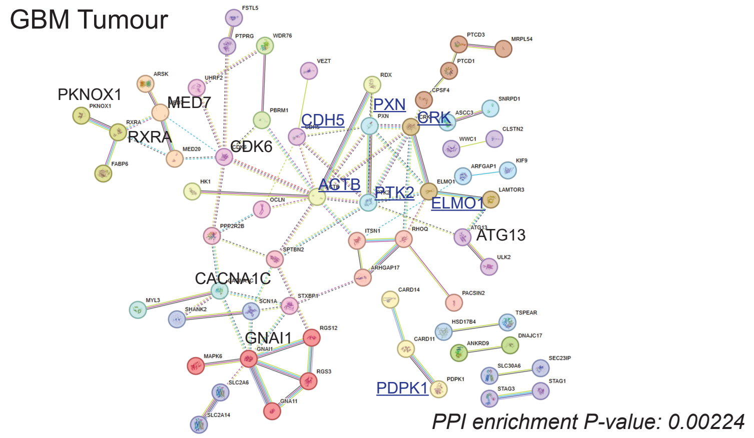

ii

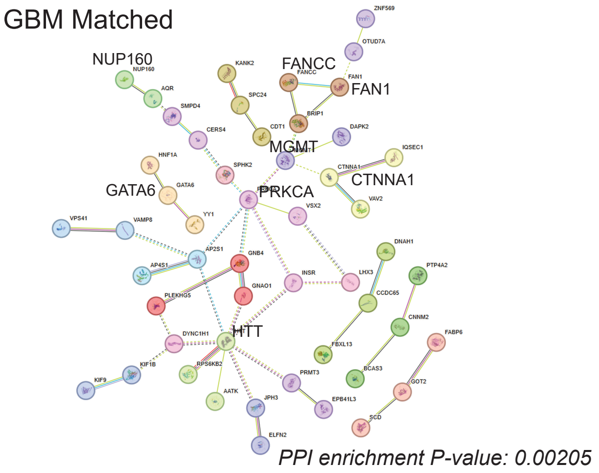

iii

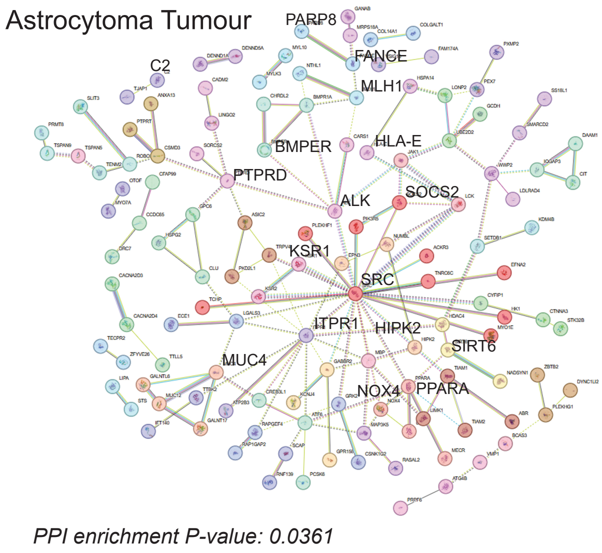

G

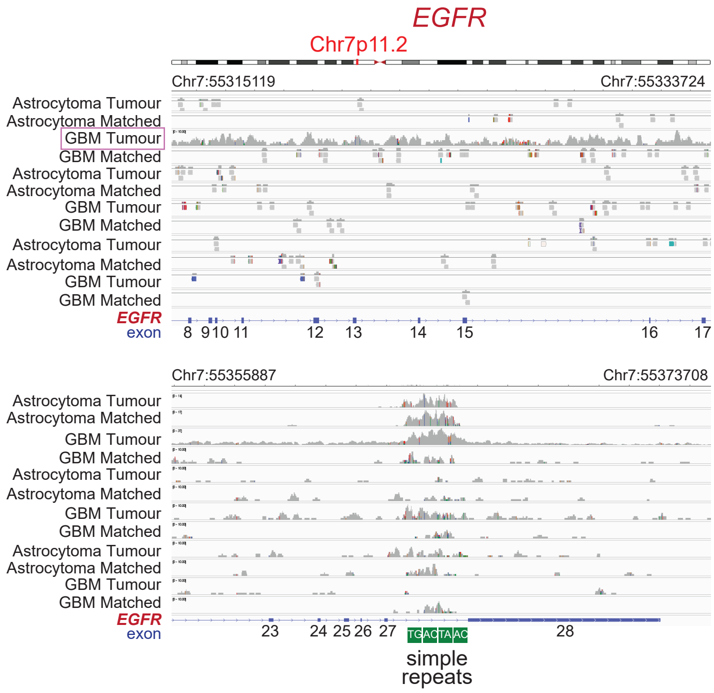

Supplemental Figure S14: eccDNA profiles reflect glioma disease pathology

Ai The incidence of 3 contiguous telomere repeat units (TTAGGG) or **ii** total SV per read pair derived from eccDNA sequencing data for 3 astrocytoma and 3 GBM patient tumour bulk and matched-adjacent tissue samples (6 samples in total per pathology) normalised to total read counts is presented as means with 95% CI. Mann-Whitney unpaired non-parametric U-tests were used to compare the groups. **B** The numbers of base pairs of microhomology (MH) at eccDNA junctions for simple and template-switch junctions sequenced from all astrocytoma and GBM patients are presented as means with 95% CI and analysed using Mann-Whitney unpaired non-parametric U-tests. **C** The **i** gene lengths and **ii** distances to the telomere for all genes identified within eccDNA amplified and sequenced from Astrocytoma, glioblastoma (GBM) or all glioma (ALL) tumour (T) and matched tissue (M) samples in base pairs (bp). The scatter plots show means with 95% CI and comparisons were made using unpaired non-parametric Mann-Whitney U-tests. **D** Glioma eccDNA were intersected with eccDNA and telomere fusions sequenced from MRC5 cells cultured in 2% or 20% O_2 for 24 hr using BEDTools 'Multiple Intersect' (Quinlan and Hall 2010). The proportions of overlaps between different glioma datasets with the totality of standard (20%) or low (2%) O_2 datasets are displayed (as a proportion of the total intersections of each glioma dataset with at least one other dataset) with pairwise evaluation using the N-1 Chi-square method. **E** Genomic intervals for glioma sample eccDNA locations were intersected with DNA repeats (defined by RepeatMasker (Smit et al. 2013)) and the proportions of each repeat class (X-axis) are displayed in a bar chart. Paired comparisons of Tumour with Matched-adjacent tissue or Astrocytoma Tumour with GBM Tumour employed the N-1 Chi-square method. **F** STRING (Szklarczyk et al. 2023) functional protein interaction networks with MCL (Markov Cluster Algorithm) clustering (inflation parameter of 4) are displayed for the genes intersecting with eccDNA derived from **i** GBM Tumour, **ii** GBM Matched-adjacent tissue or **iii** Astrocytoma Tumour. Disconnected nodes are hidden and selected proteins are annotated for clarity. Proteins implicated in the functional VEGFA-VEGFR2 pathway in GBM Tumour samples are in blue and underlined. The protein-protein interaction (PPI) statistical evaluations are indicated below the networks. No notable functional enrichments were observed for the Astrocytoma Matched-adjacent tissue. **G** Integrative Genomics Viewer (IGV) images of enriched eccDNA sequence data aligning to extended stretches of the *EGFR* gene for a single GBM tumour sample (upper panel; boxed in red) compared with the non-specific punctate signal determined at a series of simple repeats (green blocks) within the terminal intron of this gene for all glioma samples (lower panel). The human genome reference

T2T-CHM13/hs1 positions are indicated above the viewpoints. The *EGFR* exons are depicted as numbered blue blocks below.

Supplemental Figure S15

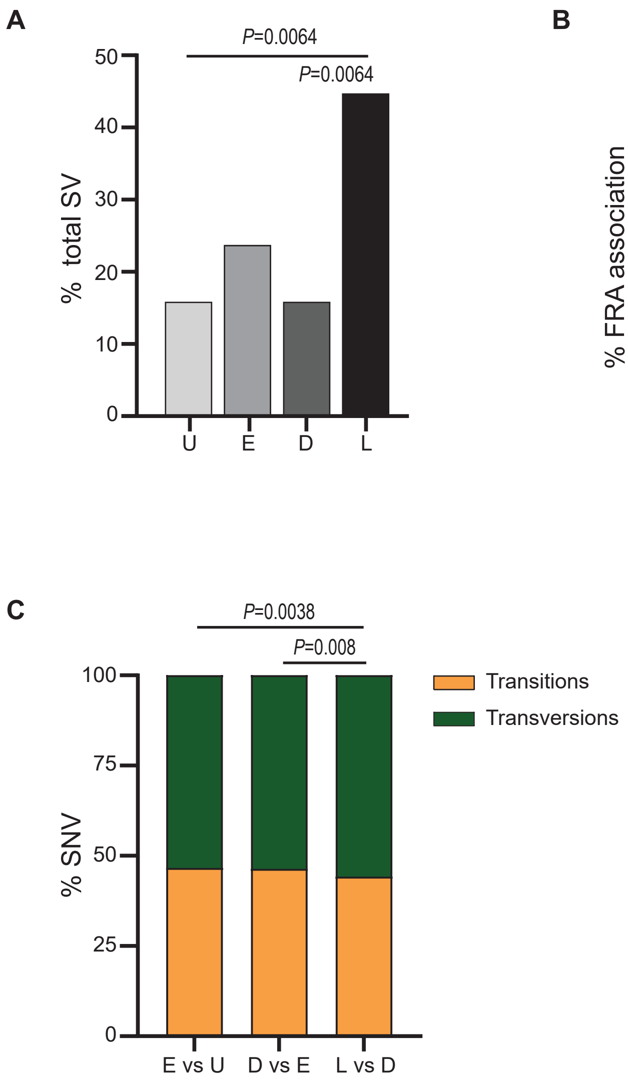

Supplemental Figure S15: Targeted capture sequencing identification of structural and single-nucleotide variants

A The proportions of all unique SV breakpoints that were identified in Untransformed MRC5 (U) and Early (E), Deep (D) and Late (L) crisis MRC5^{E6E7} cells using the Manta structural variant and indel caller (Chen et al. 2016) with targeted capture sequencing data are displayed and assessed using the N-1 Chi-square method. **B** The proportions of all unique SV breakpoints identified in the targeted capture data (ALL SV) that overlap with fragile sites (FRA) are presented for comparison with genomic intervals coinciding with fragile sites determined using 'BEDTools intersect' (Quinlan and Hall 2010) for a simulated dataset of 1 million random genomic loci (Random), all sequence-identified MRC5^{E6E7} telomere fusions (Fusions) or eccDNA derived from Untransformed (U), Early (E) or Deep (D) crisis MRC5^{E6E7} cells. Statistical significance was evaluated using the N-1 Chi-square method. **C** A Stacked bar chart displaying the relative proportions of all SNV transitions (orange, lower) and transversions (green, upper) determined for the pseudo 'tumour-normal' MRC5 sample pairs (X-axis; U, untransformed; E, Early crisis; D, Deep crisis; L, Late crisis) called using VarDict (Lai et al. 2016) in the Galaxy bioinformatics suite. Comparisons were made using the N-1 Chi-square method.

Supplemental Figure S16

A

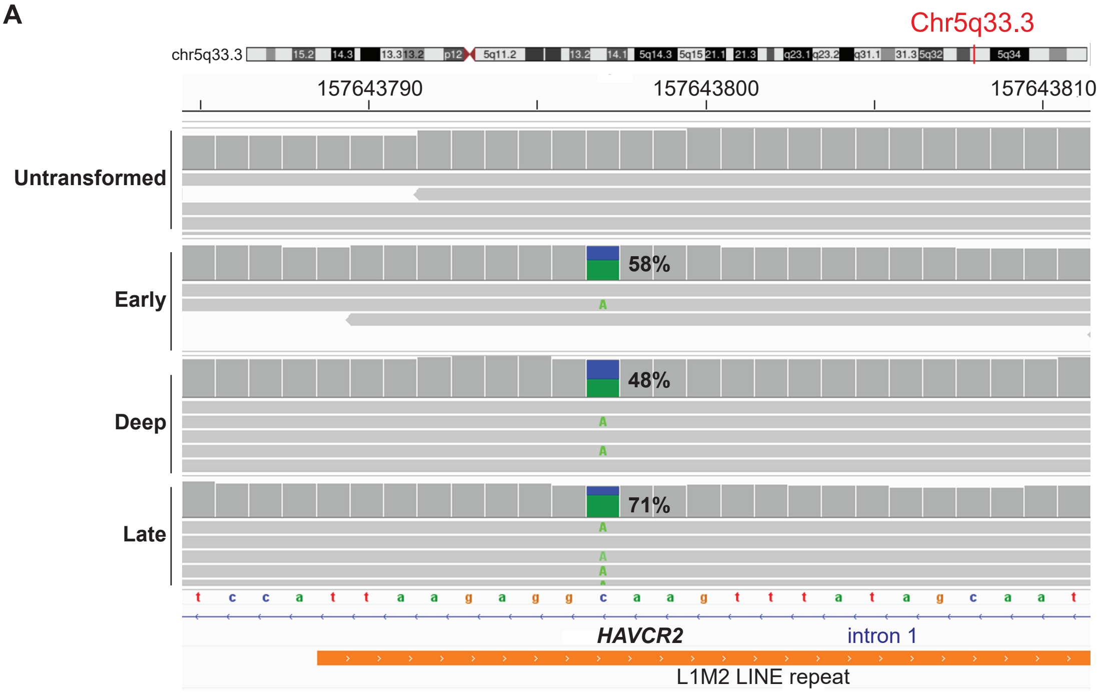

B

M00109 V$CEBPB_01 (+) RNRTKNNGMAAKNN TTAAGAGG**C**AAGTT
M00255 V$GC_01 (+) NRGGGGCGGGGCNK AAGAGG**C**AAGTTTA
M00008 V$SP1_01 (+) GRGGCRGGGW GAGG**C**AAGTT
M00272 V$P53_02 (+) NGRCWTGYCY AGG**C**AAGTTT
M00129 V$HFH1_01 NAWTGTTTATWT G**C**AAGTTTATAG
M00183 V$MYB_Q6 NNNAACKGNC G**C**AAGTTTAT

C

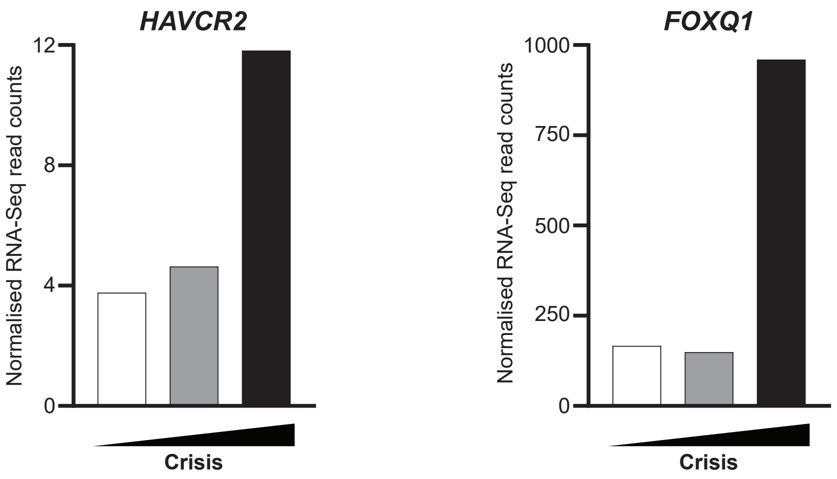

Supplemental Figure S16: Progressive C>A transversions within *HAVCR2* during replicative crisis

A Single-nucleotide variants (SNV) between sequential Untransformed-Late crisis-stage pseudo 'tumour-normal' MRC5 sample pairs were called using VarDict (Lai et al. 2016) in the Galaxy bioinformatics suite. The C>A transversion within the *HAVCR2* (*TIM3*) gene at Chr5:157643797 (visualised using IGV (Robinson et al. 2011)) is exclusive to transformed cells and enriched in the Late crisis sample (% read prevalence denoted). The position of the locus is pinpointed with the red vertical line on the Chromosome 5 ideogram at the top. The position of a LINE DNA repeat (L1M2) overlapping this site is also shown in orange below. **B** Transcription factor binding motifs potentially disrupted by the C>A transversion in *HAVCR2* were identified using the TFBind search facility (Tsunoda and Takagi 1999). The variant C nucleotide is displayed in red text within the context of the binding sites of CEBPβ, GC, SP1, TP53 (P53), FOXQ1 (HFH1) and MYB. **C** Normalised RNA-seq data for *HAVCR2* and *FOXQ1* re-analysed from our former study (Liddiard et al. 2021) using HPV16 *E6E7*-transformed MRC5 human fibroblasts sampled at progressive sampling points during replicative crisis, indicated by the black triangle below.

Supplemental Figure S17

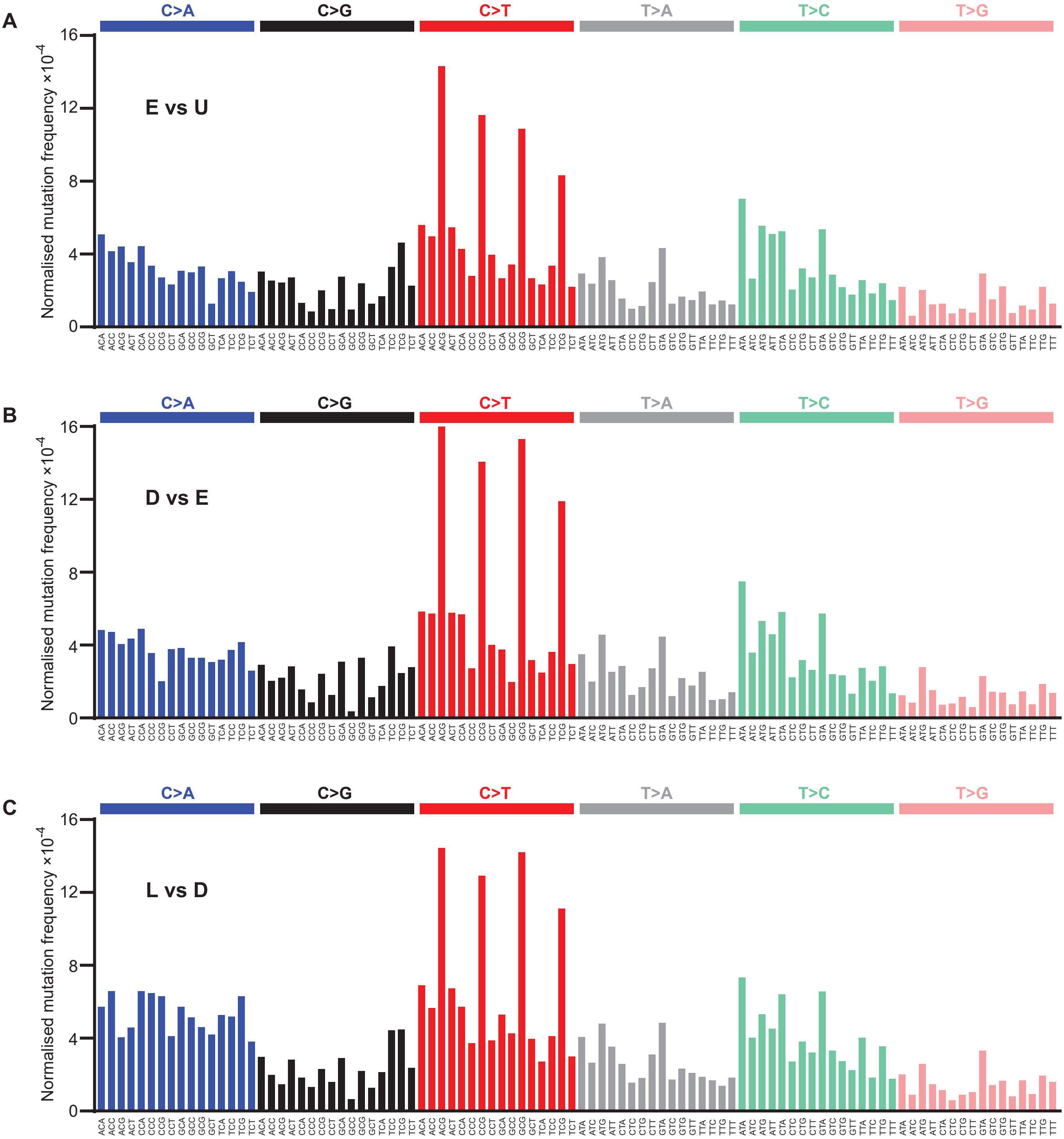

Supplemental Figure S17: Mutational signatures of ageing correspond with crisis transition

Mutational signatures manually generated from VarDict SNV calls for the same MRC5 sample pairs (**A** Early vs Untransformed, **B** Deep vs Early, **C** Late vs Deep) presented in their T2T CHM13v2.0/hs1 human reference trinucleotide context (X-axis) and normalised to the trinucleotide opportunity of the targeted capture custom panel. The coloured strips above the bars indicate the specific SNV presented in the pyrimidine space. Profiles can be compared with the mutational signatures of diverse cancer samples displayed on the Signal website (Degasperi et al. 2022).

Supplemental Figure S18

A

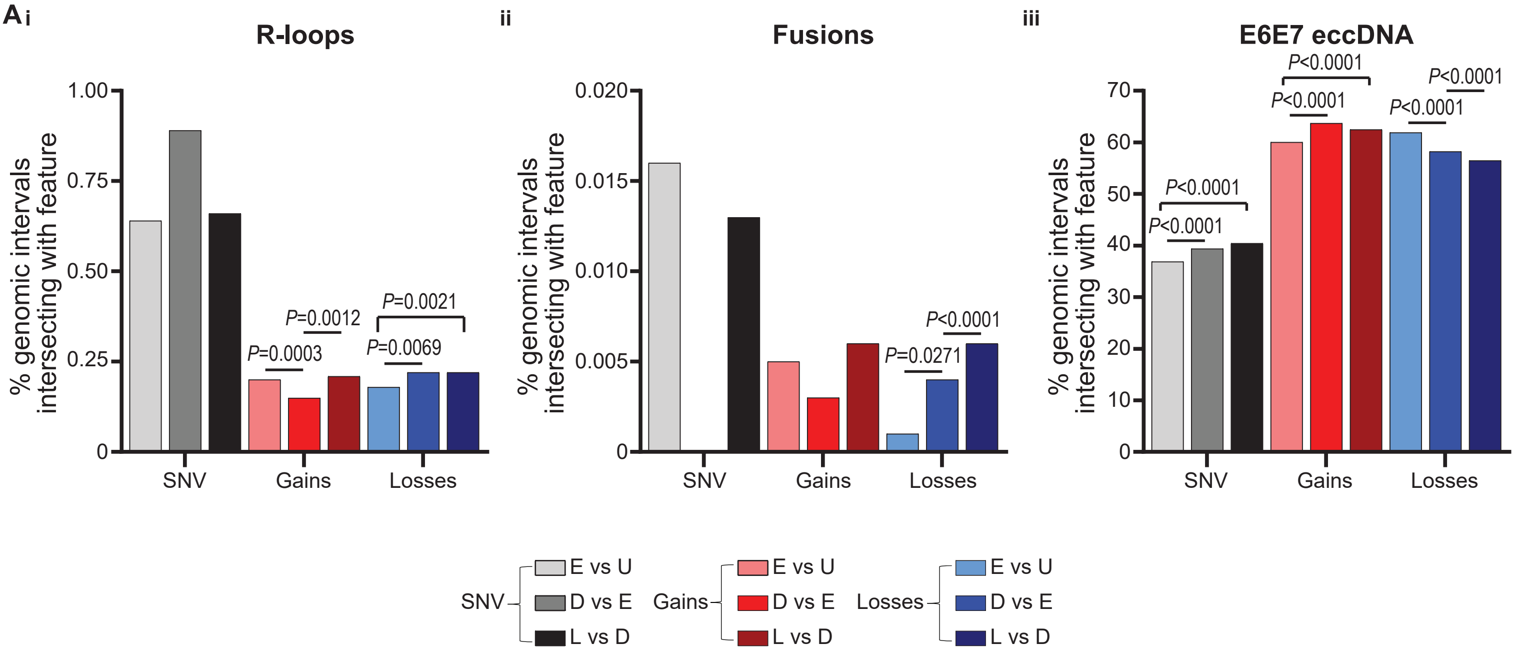

B

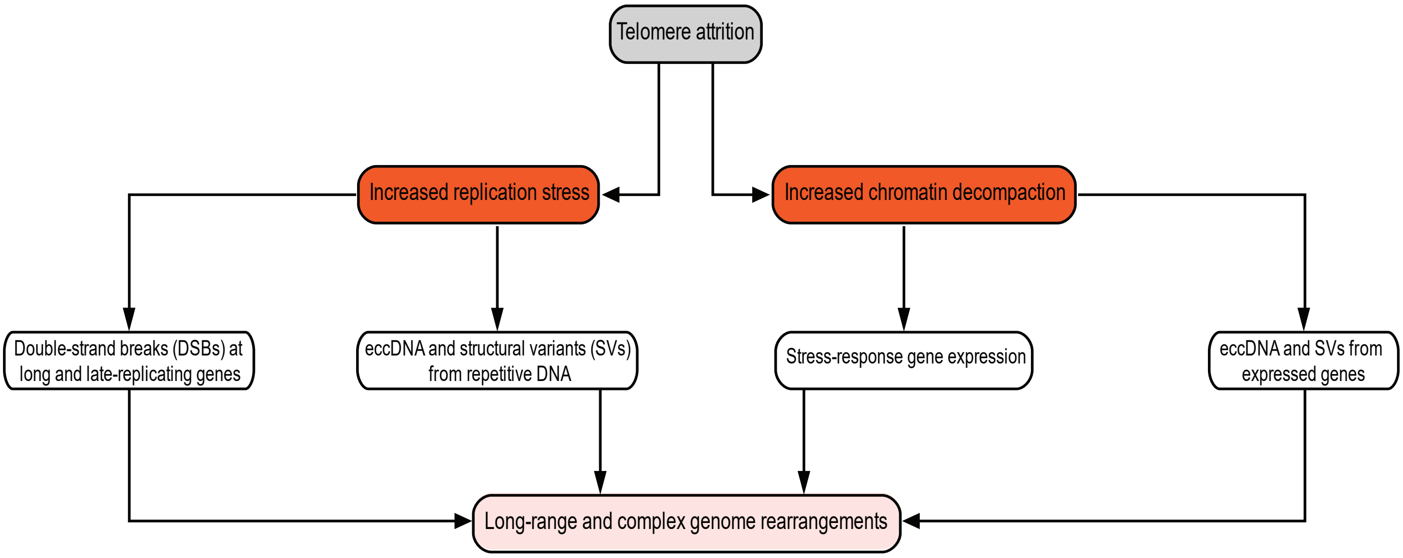

Supplemental Figure S18: Copy number alterations correlation with structural variants in MRC5 crisis samples

A VarScan 2 (Koboldt et al. 2012) 'copynumber' pseudo 'tumour-normal' pairwise analyses of Untransformed MRC5, Early, Deep and Late crisis MRC5^{E6E7} cells were performed in the Galaxy bioinformatics suite (Galaxy Community 2024). Copy Number Gains and Losses were compared with pairwise SNV outputs for the same datasets produced using VarDict (Lai et al. 2016), colour key indicated below the bar charts. The frequencies of overlaps with **i** R-loops, **ii** crisis MRC5^{E6E7} telomere fusions and **iii** eccDNA derived from transformed-only (E6E7) samples are displayed and assessed using the N-1 Chi-square method. **B** Flowchart simplifying the connections between telomere attrition, chromatin decompaction, replication stress, stress-induced transcription and the formation of structural variants in replicative crisis.

Supplemental Table S1: Chromosomal distributions and statistical comparisons of eccDNA derived from Untransformed (U) MRC5 and Early (E) and Deep (D) crisis MRC5^{E6E7} cells relating to Figure 5A

**# events**																									
**Sample**	**Total**	**Chr1**	**Chr2**	**Chr3**	**Chr4**	**Chr5**	**Chr6**	**Chr7**	**Chr8**	**Chr9**	**Chr10**	**Chr11**	**Chr12**	**Chr13**	**Chr14**	**Chr15**	**Chr16**	**Chr17**	**Chr18**	**Chr19**	**Chr20**	**Chr21**	**Chr22**	**ChrX**	**ChrY**
U	8208	720	626	473	347	429	407	454	336	389	402	429	385	246	283	240	308	382	145	399	262	130	250	149	17
E	15342	1521	1004	835	658	802	724	837	664	702	741	733	715	382	551	527	661	785	284	735	529	195	394	326	37
D	26166	2443	1865	1491	1230	1423	1294	1517	1151	1067	1299	1050	1291	750	853	946	1086	1292	549	1272	762	296	653	534	52
**% events**																									
**Sample**	**Total**	**Chr1**	**Chr2**	**Chr3**	**Chr4**	**Chr5**	**Chr6**	**Chr7**	**Chr8**	**Chr9**	**Chr10**	**Chr11**	**Chr12**	**Chr13**	**Chr14**	**Chr15**	**Chr16**	**Chr17**	**Chr18**	**Chr19**	**Chr20**	**Chr21**	**Chr22**	**ChrX**	**ChrY**
U	99.79	8.77	7.63	5.76	4.23	5.23	4.96	5.53	4.09	4.74	4.90	5.23	4.69	3.00	3.45	2.92	3.75	4.65	1.77	4.86	3.19	1.58	3.05	1.82	0.21
E	99.76	9.91	6.54	5.44	4.29	5.23	4.72	5.46	4.33	4.58	4.83	4.78	4.66	2.49	3.59	3.44	4.31	5.12	1.85	4.79	3.45	1.27	2.57	2.12	0.24
D	99.80	9.34	7.13	5.70	4.70	5.44	4.95	5.80	4.40	4.08	4.96	4.01	4.93	2.87	3.26	3.62	4.15	4.94	2.10	4.86	2.91	1.13	2.50	2.04	0.20
**χ^2 P-value[a]**																									
**Comparison**		**Chr1**	**Chr2**	**Chr3**	**Chr4**	**Chr5**	**Chr6**	**Chr7**	**Chr8**	**Chr9**	**Chr10**	**Chr11**	**Chr12**	**Chr13**	**Chr14**	**Chr15**	**Chr16**	**Chr17**	**Chr18**	**Chr19**	**Chr20**	**Chr21**	**Chr22**	**ChrX**	**ChrY**
U vs. E		0.0045	0.0017	0.3068	0.8282	1.000	0.4118	0.8221	0.3841	0.5779	0.8117	0.1288	0.9172	0.0207	0.5796	0.0322	0.0393	0.1133	0.6618	0.811	0.2914	0.0518	0.0315	0.1185	0.6467
U vs. D		0.1191	0.1275	0.8381	0.0758	0.4623	0.9709	0.3587	0.2284	0.0096	0.8269	<0.0001	0.3782	0.5404	0.4009	0.0024	0.1089	0.2869	0.0638	1.000	0.1928	0.0013	0.0066	0.2128	0.8604
E vs. D		0.0565	0.0222	0.2663	0.053	0.3593	0.2931	0.1484	0.7364	0.015	0.5542	0.0002	0.2156	0.0218	0.0726	0.339	0.4333	0.417	0.0796	0.7482	0.0022	0.2026	0.6609	0.5805	0.8273

[a] Comparison of proportions of eccDNA events along the specified chromosome for the samples indicated using the N-1 Chi-squared statistic

Supplemental Table S2: Glioma patient sample characteristics

eccDNA sample	Histological diagnosis	World Health Organization (WHO) Grade	Primary or recurrent tumour	Patient age at operation (years)
1	*IDH*-mutated astrocytoma	2	recurrent	41-45
2	Glioblastoma Multiforme (GBM)	4	primary	71-75
3	*IDH*-mutated astrocytoma	2	primary	31-35
4	Glioblastoma Multiforme (GBM)	4	primary	66-70
5	*IDH*-mutated astrocytoma	3	primary	41-45
6	Glioblastoma Multiforme (GBM)	4	primary	61-65

	Mutation status									Methylation	Variant detection	
eccDNA sample	*IDH1*	*IDH2*	*ATRX*	*TP53*	*PTEN*	*BRAF*	*EGFR*	*TERT* promoter	*H3F3A*	*MGMT*	*EGFRvIII*	*NTRK* fusions
1	MT[a]	WT[b]	MT	MT	WT	WT	WT	WT	N/A[c]	N/A	N/A	No
2	WT	WT	retained	WT	WT	WT	exon 14-15 deletion	MT	N/A	No	Yes	No
3	MT	WT	MT	MT	WT	WT	WT	WT	N/A	Yes	No	No
4	WT	WT	retained	WT	MT	WT	WT	MT	WT	Yes	N/A	No
5	MT	WT	MT	MT	WT	WT	WT	WT	N/A	Yes	No	No
6	WT	WT	retained	WT	MT	WT	WT	MT	WT	Yes	No	No

[a] Mutated gene

[b] Wildtype gene

[c] Data not available or applicable

				Mean ChrXpYp telomere length (kb)			
eccDNA sample	Ki-67[d] (max %)	H3K27M[e] substitution	GFAP expression[f]	Tumour Bulk: STELA[g]	Tumour Bulk: HT-STELA[h]	Matched-tissue: STELA	Matched-tissue: HT-STELA
1	8	negative	positive	5.08	N/A	5.18	6.76
2	20	negative	positive	3.39	3.99	5.41	4.22
3	7	negative	positive	4.71	5.23	5.27	5.27
4	15	negative	positive	5.12	6.12	N/A	4.66
5	8	negative	positive	6.11	5.18	6.14	6.36
6	50	negative	positive	3.56	4.02	3.34	3.29

[d] Detection of Ki-67 nuclear protein as an indicator of proliferative index (% of positive cells)

[e] Detection of substitution of lysine to methionine at position 27 in histone H3

[f] Detection of Glial fibrillary acidic protein (GFAP) expression

[g] Single-molecule telomere length analysis

[h] High-throughput telomere length analysis

Supplemental Table S3: Genes involved in structural variants (SV) detected in MRC5^{E6E7} cells and depicted in Figure 6A

Circos annotation	Gene symbol	Gene location
1	*CDK11B*	Chr1p36.33
2	*LRRC38*	Chr1p36.21
3	*ZRANB2-AS2*	Chr1p31.1
4	*ADGRL2*	Chr1p31.1
5	*LRRC8C*	Chr1p22.2
6	*RGS7*	Chr1q43
7	*NPAS2*	Chr2q11.2
8	*SULT1C2*	Chr2q12.3
9	*LRP1B*	Chr2q22.1-22.2
10	*BTD*	Chr3p25.1
11	*PLCXD2*	Chr3q13.2
12	*PHLDB2*	Chr3q13.2
13	*CCDC80*	Chr3q13.2
14	*LINC02066*	Chr3q25.1
15	*MCF2L2*	Chr3q27.1
16	*IQCG*	Chr3q29
17	*SPON2*	Chr4p16.3
18	*AC116345.1*	Chr5q13.2
19	*MEF2C-AS1*	Chr5q14.3
20	*AC117528.1*	Chr5q15
21	*CD109*	Chr6q13
22	*PRKN*	Chr6q26
23	*PSMG3-AS1*	Chr7p22.3
24	*CHN2*	Chr7p14.3
25	*PMS2P3*	Chr7q11.23
26	*MAGI2*	Chr7q21.11
27	*TES*	Chr7q31.2
28	*RBPMS*	Chr8p12
29	*ZFPM2*	Chr8q23.1
30	*UNC13B*	Chr9p13.3
31	*ZNF488*	Chr10q11.22
32	*LIPA*	Chr10q23.31
33	*CFAP58*	Chr10q25.1
34	*LPXN*	Chr11q12.1
35	*RAB6A*	Chr11q13.4
36	*AP001830.2*	Chr11q22.2
37	*APOBEC1*	Chr12p13.31
38	*RBMS2*	Chr12q13.3
39	*USP30*	Chr12q24.11
40	*GTF2F2*	Chr13q14.12-14.13
41	*GPC6*	Chr13q31.3-32.1
42	*AC244502.1*	Chr14q11.2
43	*NEMF*	Chr14q21.3
44	*PRKCH*	Chr14q23.1
45	*TEX22*	Chr14q32.33
46	*SNX29*	Chr16p13.13
47	*ASIC2*	Chr17q11.2
48	*FBXO47*	Chr17q12

49	*PLXDC1*	Chr17q12
50	*PCBP3*	Chr21q22.3
51	*TBL1X*	ChrXp22.31-22.2
52	*SHROOM4*	ChrXp11.22
53	*NEXMIF*	ChrXq13.3
54	*CENPI*	ChrXq22.1

Supplemental Methods

Glioma patient samples

Three each of fresh frozen lower-grade diffuse astrocytoma and glioblastoma multiforme (GBM) tumour bulk and matched-adjacent tissue samples were obtained from adult patients following informed consent, under the approval of the Welsh Neuroscience Research Tissue Bank (reference 19/WA/0058). Image-guided intra-operative multi-site sampling was conducted to ensure representative biopsies were taken. For tumour bulk, sampling was based on FLAIR-hyperintensity on T2-weighted Magnetic Resonance Imaging (MRI) for astrocytomas and contrast-enhancing disease for GBMs. Matched-adjacent non-T2 hyperintense or non-gadolinium enhancing samples were obtained from the tumour margins. The resulting 12 samples were briefly thawed on ice for extrachromosomal circular DNA (eccDNA) purification and approximately 4 mm sections rapidly pulverised using a new sterile disposable scalpel and petri dish per sample. Ground tissue was immediately added to 0.6 mL Plasmid Mini AX resuspension solution (A&A Biotechnology) in sterile 2 mL microfuge tubes for processing and circular DNA extraction.

Genomic DNA extraction

Total genomic DNA was extracted from MRC5 and $MRC5^{E6E7}$ cells and glioma tissue samples using standard Tris-HCl lysis buffer in the presence of Proteinase K and RNase A, followed by phenol/chloroform purification, precipitation with sodium acetate, and solubilisation in 10 mM Tris-HCl (pH 7.5). DNA was quantified using high sensitivity and broad range dsDNA (double-stranded DNA) assays on the Qubit™ 4 Fluorometer, as appropriate.

Telomere length PCR

Telomere length (TL) at Chromosome 17p (Chr17p) and Chromosome XpYp (ChrXpYp) was determined using 2.5 ng fibroblast genomic DNA in 4 replica reactions following the Single Telomere Length Analysis (STELA) protocol with Chr17p or ChrXpYp specific telomere-adjacent primers in combination with the Teltail primer, as described (Baird et al. 2003; Britt-Compton et al. 2006; Capper et al. 2007). For ChrXpYp allele-specific amplifications, 3.75 ng each sample was used in 4 replica reactions.

Following 0.5% Tris-acetate-EDTA (TAE) agarose gel electrophoresis, resolved TL amplicons were detected by Southern blotting using a random-primed α-33P-radiolabelled telomere repeat sequence probe combined with a probe to detect the molecular weight markers. Quantification of chromosome-specific TL was performed using ImageQuant TL v 8.2 (Cytiva).

TL analyses of glioma patients were conducted using both STELA and a high throughput permutation (Norris et al. 2021).

Telomere fusion PCR and amplicon sequencing

Telomere fusion amplicons were generated from fibroblast genomic DNA by multiplex long-range PCR using primers targeting the Chr17p and ChrXpYp subtelomeres and Chr21q family of homologous telomeres.

To define the onset of Chr17p and ChrXpYp telomere dysfunction ahead of long-range chromatin interaction experiments, the fusion PCR reactions employed 2 primers in the same orientation targeting either the Chr17p or the ChrXpYp subtelomeres (Supplemental Figure S1B). Four replica reactions each using 50 ng input genomic DNA were performed. The primer sequences are listed in Supplemental Methods Table S1.

To investigate the impact of low O_2 conditions, 3 primers targeting Chr17p, ChrXpYp and Chr21q were used (17p6, XpYpM and 21q1 primers, respectively) (Britt-Compton et al. 2006; Capper et al. 2007; Letsolo et al. 2010). For fusion amplicon sequencing, 40 replica 3 primer fusion reactions using 100 ng input DNA each were conducted per sample. Replicas were subsequently pooled for purification using Agencourt AMPure XP magnetic beads. Purified amplicon pools were sheared to a mean length of 450 base pairs (bp) using the Covaris M220 focused ultrasonicator and libraries prepared using the New England Biolabs NEBNext® Ultra™ II DNA Library Prep Kit for Illumina® (E7645S) with Primer Set 3 of the NEBNext® Multiplex Oligos for Illumina® (E7710S). Paired-end sequencing (2 × 150 bp) of multiplexed samples was executed using the Illumina NextSeq 550 Mid Output platform.

For Sanger sequencing of fusion amplicons, reamplification PCRs were first performed using the same or nested primers to generate sufficient product for UV visualisation. Amplicons were resolved by agarose gel electrophoresis, excised and extracted using the Monarch DNA Gel Extraction Kit (NEB). Purified amplicons were quantified using the Qubit™ 4 Fluorometer and sequenced using the Eurofins Genomics TubeSeq service. Alignments were identified and verified using the University of California Santa Cruz Genomics Institute (UCSC) Genome Browser (Nassar et al. 2023) BLAT (Kent 2002) webtool.

Crisis cell telomere fusion and RNA-seq data

Sequence data re-analysed from our previous publication (Liddiard et al. 2021) is available from National Center for Biotechnology Information Sequence Read Archive (https://www.ncbi.nlm.nih.gov/sra) under project code PRJNA659174.

Identification of telomere fusion and eccDNA structural variants

Code employed in this study is available at https://github.com/e-coral/reads2SVs and in the Supplemental code folder within the Supplemental material.

From the FASTQ sequencing output files, duplicate reads were marked using SAMBLASTER (version 0.1.26) (Faust and Hall 2014). Reads were sorted and indexed using SAMtools 1.18 (Danecek et al. 2021). Illumina short reads were aligned to the T2T-CHM13v2.0 release of the human genome (Nurk et al. 2022) using the BWA-MEM algorithm (Li 2013) via the BWA tool (version 0.7.17-r1188). PacBio long reads were aligned to the same reference genome using BWA-MEM (Version: 0.7.19-r1273) in default mode. Telomere fusion and eccDNA junctions ('structural variants'; SV) were called using dysgu (version 1.6.1 for Illumina sequencing; version 1.8.7 for PacBio sequencing) (Cleal and Baird 2022). Sites exhibiting non-specific signal (AC repeat blocks within *IGH*, *TAFA5* and *PIGU*; mitochondrial homology at *DHFR*) were excluded from downstream analyses and individual recombinations of interest were investigated using BLAT (Kent 2002). Junction analyses and curation of inverted telomere repeats in fusions and eccDNA were conducted by manual BLAT of sequence reads.

Structural variant resolution using targeted capture sequencing

Targeted capture sequencing reads for Untransformed MRC5 and Early (PD23), Deep (PD47) and Late (PD57) crisis $MRC5^{E6E7}$ cells were mapped to the T2T-CHM13v2.0 human reference (Nurk et al. 2022). Sample BAM files and the T2T-CHM13v2.0 human reference FASTA file were uploaded into the main and EU servers of the Galaxy bioinformatics suite version 25.0 (Galaxy Community 2024).

Structural variants (SV) were called from pseudo tumour-normal MRC5 sample pairs, where the 'tumour' sample constituted the more advanced in the crisis series and the 'normal' sample was the precursory sample (Early crisis vs Untransformed, Deep vs Early crisis, Late vs Deep crisis) using Manta (Chen et al. 2016) Galaxy Version 1.6+galaxy9). The default configuration was modified to turn depth filters off and include unfiltered variants. 'Breakend' (BND) SV were filtered from the Variant Call Format (VCF) outputs for manual inspection in IGV (Robinson et al. 2011) and

determination of sample origins and specificity. Non-unique SV and those aligning to capture probe sites (that could represent artificial chimeras) were excluded from further study.

Single nucleotide variant calling from targeted capture sequencing

VarDict (Lai et al. 2016) (Galaxy Version 1.8.3+galaxy1) was used to call somatic single nucleotide variants (SNV) and indels for the same pseudo tumour-normal MRC5 sample pairings as detailed above against the T2T-CHM13v2.0 human reference (Altemose et al. 2022; Nurk et al. 2022) and with local realignment enabled. We performed reverse analyses (setting the more advanced crisis sample as the 'normal' rather than the 'tumour' in the sample pairings) as a stringent control to verify that the observed results were relevant to crisis progression. Since VarDict is a highly-sensitive tool, outputs were stringently filtered using VCF filter (Galaxy Version 1.0.0_rc3+galaxy3) prior to SNV analyses. 'Passed' events (with ChrM removed) were filtered on single nucleotide events categorised as 'LikelySomatic' or 'StronglySomatic', read-depths of > 10 and SSF VarDict P-value calls of < 0.05. Evaluation of nucleotide transitions and transversions amongst SNV, collapsed and expressed in the pyrimidine space, was done by manual data sorts and counts.

'Mutational signatures' were derived manually to ensure accurate assessment of variation within the repetitive regions of the genome (Aganezov et al. 2022) relevant to this study within the context of the T2T-CHM13v2.0 human reference (Altemose et al. 2022; Nurk et al. 2022). The trinucleotide context of each SNV on both the sense and antisense strand was extracted from the USCC Table Browser. To normalise trinucleotide frequencies associated with each SNV to the trinucleotide opportunity, trinucleotide counts for the custom target capture panel (covering 8,050,562 bp) were performed using compseq (Galaxy Version 5.0.0.1) within the Galaxy bioinformatics suite. For each separate SNV, the observed frequency of each trinucleotide context was compared to the total trinucleotide count within the custom panel to produce the normalised mutation frequency.

Copy number variant calling from targeted capture sequencing

Using a similar pairwise sample comparison strategy as with SNV calling, VarScan 2 (Koboldt et al. 2012, 2013) copynumber was deployed within the Galaxy bioinformatics suite (Galaxy Version 2.4.3.2) (Galaxy Community 2024). Individual targeted capture sequencing sample BAM files were first converted to pileup datasets using Generate pileup (Galaxy Version 1.1.3) without consensus calling according to the MAQ model. Relative tumour copy number was established from pseudo tumour-normal pairs (Early crisis vs Untransformed, Deep vs Early crisis, Late vs Deep

crisis MRC5) with the following settings: minimum read depth of 20, minimum base quality to count for coverage of 20, minimum read mapping quality to count for coverage of 10, minimum number of consecutive bases to report a segment of 10, maximum size before a new segment is made of 100, P-value threshold for significant copy number change-point of 0.01 and a normal/tumour input data ratio for copy number adjustment of 1 (based on balanced read outputs). Each output was separated into copy number 'gains' ($\log_2$ ratio tumour/normal > 0.3) and 'losses' ($\log_2$ ratio tumour/normal < -0.3) for analyses.

Generation of random loci

A dataset of 1 million single nucleotide random genomic locations (excluding ChrM) was generated using BEDTools RandomBed (Galaxy Version 2.31.1+galaxy1) for comparison with intersections of experimental datasets.

Data annotations

Regions of interest (SV and signal peaks) were annotated with features of interest using the Python module available at https://github.com/e-coral/annotations and detailed in the Supplemental code folder in the Supplemental material. Features of interest included: gene identities and lengths (Fiddes et al. 2018; Shumate and Salzberg 2021; Goldfarb et al. 2025), expressed genes, DNA repeat motifs (Smit et al. 2013) (including centromere satellite [CenSat] repeats (Altemose et al. 2022)) and fragile sites (Kumar et al. 2019), as well as the distance between features and the telomere on the same chromosome arm. Coincidence between regions and features of interest was determined by identifying overlaps using the Nested Containment List (NCLS) Python package (Stovner and Sætrom 2020).

BEDTools intersections of experimental datasets with feature tracks were also performed in the Galaxy bioinformatics suite (Galaxy Community 2024) (BEDTools Intersect intervals; Galaxy Version 2.31.1+galaxy0) (Quinlan and Hall 2010). To determine the proportions of dataset genomic intervals overlapping with specific features, a "left outer join" was performed, returning the presence or absence of an overlap for each event in the input file. To evaluate the diversity and frequency of DNA repeat motifs associating with experimental datasets, the original entry in B (RepeatMasker input file (Smit et al. 2013)) was written for each overlap with no null events returned. The abundance of each repeat motif as a proportion of the total repeats overlaps for each sample was reported.

Genes:

Genes and their positions within the T2T-CHM13v2.0 human reference (Nurk et al. 2022) were obtained from two sources: RefSeq (O'Leary et al. 2016; Goldfarb et al. 2025) for T2T (John Hopkins University Center for Computational Biology 2023) and CAT/Liftoff (Fiddes et al. 2018; Shumate and Salzberg 2021) via the UCSC Table Browser (Karolchik et al. 2004). Gene lengths were calculated as the distance between the start and end position for each gene for each of the original gene data sources.

Gene promoters:

The positions of human gene promoters were taken from the Eukaryotic Promoter Database (Cavin Périer et al. 1998) tracks obtained via the UCSC Table Browser (Karolchik et al. 2004). Genome assembly GRCh38 coordinates were converted to the T2T-CHM13v2.0 human reference (Nurk et al. 2022) using the liftOver tool (Hinrichs et al. 2006).

Expressed genes:

Normalised RNA-seq read counts pertaining to $MRC5^{E6E7}$ cells transiting replicative crisis were taken from our previous study (Liddiard et al. 2021). Genes with signal > 1 at any of the 3 sampling points (PD45, PD49 or PD57) were classified as 'expressed' and their GRCh38 genomic locations (duplicate entries removed) transposed to the T2T-CHM13v2.0 human reference (Nurk et al. 2022) using the UCSC liftOver tool (Hinrichs et al. 2006).

DNA repeats:

RepeatMasker defined repetitive motifs (Smith and Waterman 1981) for the T2T-CHM13v2.0 human reference (Nurk et al. 2022) were obtained from the UCSC resources repository (Nassar et al. 2023). Centromere satellite (CenSat) repeats (Altemose et al. 2022) were obtained separately using the Table Browser feature (Karolchik et al. 2004).

Fragile sites:

Cytogenetic information pertaining to both common and rare fragile sites was obtained from the HUGO Gene Nomenclature Committee (Seal et al. 2023). Positional information was cross-referenced with the UCSC resources repository (Nassar et al. 2023) to create a T2T-CHM13v2.0 reference file. Additional fragile sites documented in the HumCFS database (Kumar et al. 2019) were manually appended.

R-loops:

R-loop consensus tracks based on 23 published S9.6 antibody DRIP-seq datasets (Chédin et al. 2021) were taken from (Kishkevich et al. 2022). The track filtered on a peak score threshold of 200 was transposed to the T2T-CHM13v2.0

human reference (Nurk et al. 2022) using the UCSC liftOver tool (Hinrichs et al. 2006) with additional manual curation of ChrUn sites. ChrM locations were removed.

Distance to the telomere:

To report the distance between a feature of interest and the telomere on the same chromosome arm, telomere (start of telomere repeat array) and centromere (based on cytobands ideogram) positions were first defined for the T2T-CHM13v2.0 human reference (Nurk et al. 2022). Centromere positions were used to orient the feature of interest on the appropriate chromosome arm (p- or q-arm). Distance to the telomere boundary was then calculated from the start (p-arm) or end (q-arm) of the feature, as appropriate.

Intersections of multiple datasets

To assess parity between datasets and identify sites of recurrent vulnerability, we utilised the BEDTools Multiple Intersect tool (BEDTools multiinter) (Quinlan and Hall 2010) within the Galaxy bioinformatics suite (Galaxy Version 2.31.1; Bjoern A. Gruening (2014), Galaxy wrapper) (Galaxy Community 2024). Annotations were removed from BED files before intersection and Ryan Layers' clustering algorithm was invoked to group overlapping intervals into clusters (Quinlan and Hall 2010). Empty regions were not reported to constrain file outputs sizes. Results were filtered for genomic intervals shared by $\geq$ 2 datasets.

The UpSet (Lex et al. 2014) plot tool (Galaxy Version 0.6.5+galaxy2) (Khan and Mathelier 2017) was used to produce pairwise intersects of multiple (unannotated) BED files for visualisation (see also data presentation below).

Gene Ontology and protein networks

Ontology searches of output gene lists were performed using the Gene Ontology enrichment analysis tool (Mi et al. 2013), Gene Set Enrichment Analysis (GSEA) (MSigDB 2024.1) (Mootha et al. 2003; Subramanian et al. 2005) and Shiny GO 0.80 (Ge et al. 2020), with pathway analyses utilising KEGG (Kyoto Encyclopedia of Genes and Genomes) mapping (Kanehisa et al. 2021) and Pathview (Luo and Brouwer 2013). Differential ontology assessments employed GOrilla (Eden et al. 2009). Functional protein interaction networks were explored using STRING (version 12.0 © STRING Consortium 2025) (Szklarczyk et al. 2023).

DNA motifs

DNA sequences flanking the SV or region of interest were extracted from the UCSC Table Browser (Karolchik et al. 2004). Evaluation of non-B DNA motifs within sequence extending 5 kb from the 3′ termini of genes associated with 3′ ssDNA was made using the nBMST webtool (Cer et al. 2012). The incidence of each motif within the input sequences was calculated from the motif occurrence and total length of the input sequences.

To identify novel and known DNA binding factor motifs associated with genes captured by and fused to telomeres, 5 kb DNA sequence both 5′ and 3′ the Capture-C interaction point within each gene was analysed using the XTREME tool within the MEME motif discovery suite version 5.5.5 (Grant and Bailey 2021). Sequences were queried against human motifs (HOCOMOCO human v11 FULL database) with widths of 4-12 bp. Relative enrichment was determined by comparison with either scrambled input or relevant control datasets, with E-values set at $\leq$ 0.05.

To propose transcription factor binding sites potentially disrupted by single nucleotide variants detected in targeted capture experiments, events with 300 bp flanking sequence (1 read length either side of the variant) were queried using the TFBIND webtool (Tsunoda and Takagi 1999).

cBioPortal mining

To assess clinical significance of mutations or deregulated expression of genes of interest, public cancer databases were interrogated. Genes of interest were queried against 2922 samples sequenced in a study by the Pan-Cancer Analysis of Whole Genomes (PCAWG) Consortium of the International Cancer Genome Consortium (ICGC) and The Cancer Genome Atlas (TCGA) (ICGC/TCGA Pan-Cancer Analysis of Whole Genomes Consortium 2020) via the cBioPortal for Cancer Genomics (https://www.cbioportal.org/). Mutations, copy number and mRNA expression *Z*-scores (threshold of 2.0) relative to all samples for each queried gene were returned. Oncoprint was employed to display and evaluate abnormality categories and proportions amongst the sampled population. Survival information for patients bearing altered compared with unaltered genes was extracted to assess prognostic significance. Logrank test P-values and hazard ratios (HR) with 95% confidence intervals (CI) were performed by the webtool.

Data presentation

Bar, stacked, line and pie charts were plotted using GraphPad Prism 10.

Protein interaction networks and lollipop plots were exported from the STRING consortium webtool (https://string-db.org/cgi/aboutdatabases) (Szklarczyk et al. 2023). Network nodes represent proteins and interaction edges indicate predicted or known functional associations. Lollipop plots provide visualisation of the functional enrichments within

datasets. Each enriched term is depicted as a coloured circle that indicates both the number of proteins in the network associated with the term and the False Discovery Rate (FDR). The STRING enrichment signal is a composite score that balances enrichment magnitude (observed/expected) and statistical significance (FDR), reducing bias toward either very large or very small functional categories.

Views of genomic locations were exported from the Integrative Genomics Viewer (version 2.17.2) (Robinson et al. 2011) that depicts genomic features aligned to reference coordinates.

Kaplan-Meier patient survival curves were exported from cBioPortal (ICGC/TCGA Pan-Cancer Analysis of Whole Genomes Consortium 2020) to reveal the differences in survival of groups with altered or unaltered genes or gene expression. The Logrank Test evaluates the null hypothesis that there is no difference between the groups and the Hazard Ratio derived from this indicates the magnitude of the effect.

DNA motif logos for consensus binding and sample-enriched sequences were produced by the Tomtom tool (Gupta et al. 2007) within the MEME suite (Bailey et al. 2015) of motif-based DNA sequence analysis tools. Each logo X-axis indicates the position along the motif, while the Y-axis shows information content (bits). At each position, the total stack height reflects sequence conservation and the relative heights of the letters represent the nucleotide frequencies at that position.

Circos plots displaying genomic recombinations and long-range interactions were produced using ShinyCircos V2.0 (Yu et al. 2018). The circular chromosomal layout (sequential Chr1-22, ChrX, ChrY) highlights inter- and intra-chromosomal relationships and the perimeter tracks can display either discrete genomic positions or quantitative signals, such as feature frequency or read counts.

UpSet (Lex et al. 2014) (Galaxy Version 0.6.5+galaxy2) (Khan and Mathelier 2017), Parallel Coordinates (Galaxy Version 0.2; https://plotly.com/) and Principal Component Analysis (PCA) plots (https://plotly.com/) were generated in the Galaxy bioinformatics suite (Galaxy version 25.0.2.dev0) (Galaxy Community 2024). UpSet plots summarise the overlaps between multiple datasets, with connecting lines indicating the datasets involved in each intersection, vertical bars showing the number of overlaps and the lower horizontal bars depicting the total dataset sizes. Parallel Coordinates charts display multivariate data by mapping each parameter to a parallel axis and connecting observations with a continuous line across all variables. PCA plots reduce the complexity of high-dimensional data by distilling it into principal components that describe the most variation in the data. Data is plotted against the

principle components that confer the most variance to identify similarities and differences between sets. Points closer together on the plot are more similar than points further apart, allowing visualisation of groupings and influential variables.

Use of generative AI

OpenAI ChatGPT (GPT-5; August 2025 version) was employed to assess the variance contributed by individual features input into the Principal Component analyses in order to determine the sufficiency and effectiveness of selected constituents. Results were verified using Microsoft Copilot (GPT-4; August 2025).

Statistics

Unless otherwise documented, statistical analyses were conducted using GraphPad Prism 10 and Medcalcs free statistical calculators (https://www.medcalc.org/). Appropriate parametric or nonparametric statistical tests were selected based on data distribution (Gaussian or skewed) and experimental design (paired or independent samples, discrete or continuous data). Unless otherwise described, data are presented as means with 95% confidence intervals (CIs). Statistical evaluations of Gene Ontology searches and differential sample enrichments were embedded in the relevant webtools.

Supplemental Methods References

Aganezov S, Yan SM, Soto DC, Kirsche M, Zarate S, Avdeyev P, Taylor DJ, Shafin K, Shumate A, Xiao C, et al. 2022. A complete reference genome improves analysis of human genetic variation. *Science* **376**: eabl3533.

Altemose N, Logsdon GA, Bzikadze AV, Sidhwani P, Langley SA, Caldas GV, Hoyt SJ, Uralsky L, Ryabov FD, Shew CJ, et al. 2022. Complete genomic and epigenetic maps of human centromeres. *Science* **376**: eabl4178.

Bailey TL, Johnson J, Grant CE, Noble WS. 2015. The MEME Suite. *Nucleic Acids Res* **43**: W39-49.

Baird DM, Rowson J, Wynford-Thomas D, Kipling D. 2003. Extensive allelic variation and ultrashort telomeres in senescent human cells. *Nat Genet* **33**: 203–207.

Britt-Compton B, Rowson J, Locke M, Mackenzie I, Kipling D, Baird DM. 2006. Structural stability and chromosome-specific telomere length is governed by cis-acting determinants in humans. *Hum Mol Genet* **15**: 725–733.

Capper R, Britt-Compton B, Tankimanova M, Rowson J, Letsolo B, Man S, Haughton M, Baird DM. 2007. The nature of telomere fusion and a definition of the critical telomere length in human cells. *Genes Dev* **21**: 2495–2508.

Cavin Périer R, Junier T, Bucher P. 1998. The eukaryotic promoter database EPD. *Nucleic Acids Res* **26**: 353–357.

Cer RZ, Bruce KH, Donohue DE, Temiz NA, Mudunuri US, Yi M, Volfovsky N, Bacolla A, Luke BT, Collins JR, et al. 2012. Searching for non-B DNA-forming motifs using nBMST (non-B DNA motif search tool). *Curr Protoc Hum Genet* **Chapter 18**: Unit 18.7.1-22.

Chédin F, Hartono SR, Sanz LA, Vanoosthuyse V. 2021. Best practices for the visualization, mapping, and manipulation of R-loops. *EMBO J* **40**: e106394.

Chen X, Schulz-Trieglaff O, Shaw R, Barnes B, Schlesinger F, Källberg M, Cox AJ, Kruglyak S, Saunders CT. 2016. Manta: rapid detection of structural variants and indels for germline and cancer sequencing applications. *Bioinformatics* **32**: 1220–1222.

Cleal K, Baird DM. 2022. Dysgu: efficient structural variant calling using short or long reads. *Nucleic Acids Res* **50**: e53.

Danecek P, Bonfield JK, Liddle J, Marshall J, Ohan V, Pollard MO, Whitwham A, Keane T, McCarthy SA, Davies RM, et al. 2021. Twelve years of SAMtools and BCFtools. *Gigascience* **10**.

Eden E, Navon R, Steinfeld I, Lipson D, Yakhini Z. 2009. GOrilla: a tool for discovery and visualization of enriched GO terms in ranked gene lists. *BMC Bioinformatics* **10**: 48.

Faust GG, Hall IM. 2014. SAMBLASTER: fast duplicate marking and structural variant read extraction. *Bioinformatics* **30**: 2503–2505.

Fiddes IT, Armstrong J, Diekhans M, Nachtweide S, Kronenberg ZN, Underwood JG, Gordon D, Earl D, Keane T, Eichler EE, et al. 2018. Comparative Annotation Toolkit (CAT)-simultaneous clade and personal genome annotation. *Genome Res* **28**: 1029–1038.

Galaxy Community. 2024. The Galaxy platform for accessible, reproducible, and collaborative data analyses: 2024 update. *Nucleic Acids Res* **52**: W83–W94.

Ge SX, Jung D, Yao R. 2020. ShinyGO: a graphical gene-set enrichment tool for animals and plants. *Bioinformatics* **36**: 2628–2629.

Goldfarb T, Kodali VK, Pujar S, Brover V, Robbertse B, Farrell CM, Oh D-H, Astashyn A, Ermolaeva O, Haddad D, et al. 2025. NCBI RefSeq: reference sequence standards through 25 years of curation and annotation. *Nucleic Acids Res* **53**: D243–D257.

Grant CE, Bailey TL. 2021. XSTREME: Comprehensive motif analysis of biological sequence datasets. *BioRxiv*.

Gupta S, Stamatoyannopoulos JA, Bailey TL, Noble WS. 2007. Quantifying similarity between motifs. *Genome Biol* **8**: R24.

Hinrichs AS, Karolchik D, Baertsch R, Barber GP, Bejerano G, Clawson H, Diekhans M, Furey TS, Harte RA, Hsu F, et al. 2006. The UCSC Genome Browser Database: update 2006. *Nucleic Acids Res* **34**: D590-8.

ICGC/TCGA Pan-Cancer Analysis of Whole Genomes Consortium. 2020. Pan-cancer analysis of whole genomes. *Nature* **578**: 82–93.

Kanehisa M, Furumichi M, Sato Y, Ishiguro-Watanabe M, Tanabe M. 2021. KEGG: integrating viruses and cellular organisms. *Nucleic Acids Res* **49**: D545–D551.

Karolchik D, Hinrichs AS, Furey TS, Roskin KM, Sugnet CW, Haussler D, Kent WJ. 2004. The UCSC Table Browser data retrieval tool. *Nucleic Acids Res* **32**: D493-6.

Kent WJ. 2002. BLAT--the BLAST-like alignment tool. *Genome Res* **12**: 656–664.

Khan A, Mathelier A. 2017. Intervene: a tool for intersection and visualization of multiple gene or genomic region sets. *BMC Bioinformatics* **18**: 287.

Kishkevich A, Tamang S, Nguyen MO, Oehler J, Bulmaga E, Andreadis C, Morrow CA, Jalan M, Osman F, Whitby MC. 2022. Rad52's DNA annealing activity drives template switching associated with restarted DNA replication. *Nat Commun* **13**: 7293.

Koboldt DC, Larson DE, Wilson RK. 2013. Using VarScan 2 for Germline Variant Calling and Somatic Mutation Detection. *Curr Protoc Bioinformatics* **44**: 15.4.1-15.4.17.

Koboldt DC, Zhang Q, Larson DE, Shen D, McLellan MD, Lin L, Miller CA, Mardis ER, Ding L, Wilson RK. 2012. VarScan 2: somatic mutation and copy number alteration discovery in cancer by exome sequencing. *Genome Res* **22**: 568–576.

Kumar R, Nagpal G, Kumar V, Usmani SS, Agrawal P, Raghava GPS. 2019. HumCFS: a database of fragile sites in human chromosomes. *BMC Genomics* **19**: 985.

Lai Z, Markovets A, Ahdesmaki M, Chapman B, Hofmann O, McEwen R, Johnson J, Dougherty B, Barrett JC, Dry JR. 2016. VarDict: a novel and versatile variant caller for next-generation sequencing in cancer research. *Nucleic Acids Res* **44**: e108.

Letsolo BT, Rowson J, Baird DM. 2010. Fusion of short telomeres in human cells is characterized by extensive deletion and microhomology, and can result in complex rearrangements. *Nucleic Acids Res* **38**: 1841–1852.

Lex A, Gehlenborg N, Strobelt H, Vuillemot R, Pfister H. 2014. Upset: visualization of intersecting sets. *IEEE Trans Vis Comput Graph* **20**: 1983–1992.

Liddiard K, Grimstead JW, Cleal K, Evans A, Baird DM. 2021. Tracking telomere fusions through crisis reveals conflict between DNA transcription and the DNA damage response. *NAR Cancer* **3**: zcaa044.

Li H. 2013. Aligning sequence reads, clone sequences and assembly contigs with BWA-MEM. *arXiv*.

Luo W, Brouwer C. 2013. Pathview: an R/Bioconductor package for pathway-based data integration and visualization. *Bioinformatics* **29**: 1830–1831.

Mi H, Muruganujan A, Casagrande JT, Thomas PD. 2013. Large-scale gene function analysis with the PANTHER classification system. *Nat Protoc* **8**: 1551–1566.

Mootha VK, Lindgren CM, Eriksson K-F, Subramanian A, Sihag S, Lehar J, Puigserver P, Carlsson E, Ridderstråle M, Laurila E, et al. 2003. PGC-1alpha-responsive genes involved in oxidative phosphorylation are coordinately downregulated in human diabetes. *Nat Genet* **34**: 267–273.

Nassar LR, Barber GP, Benet-Pagès A, Casper J, Clawson H, Diekhans M, Fischer C, Gonzalez JN, Hinrichs AS, Lee BT, et al. 2023. The UCSC Genome Browser database: 2023 update. *Nucleic Acids Res* **51**: D1188–D1195.

Norris K, Walne AJ, Ponsford MJ, Cleal K, Grimstead JW, Ellison A, Alnajar J, Dokal I, Vulliamy T, Baird DM. 2021. High-throughput STELA provides a rapid test for the diagnosis of telomere biology disorders. *Hum Genet* **140**: 945–955.

Nurk S, Koren S, Rhie A, Rautiainen M, Bzikadze AV, Mikheenko A, Vollger MR, Altemose N, Uralsky L, Gershman A, et al. 2022. The complete sequence of a human genome. *Science* **376**: 44–53.

O'Leary NA, Wright MW, Brister JR, Ciufo S, Haddad D, McVeigh R, Rajput B, Robbertse B, Smith-White B, Ako-Adjei D, et al. 2016. Reference sequence (RefSeq) database at NCBI: current status, taxonomic expansion, and functional annotation. *Nucleic Acids Res* **44**: D733-45.

Quinlan AR, Hall IM. 2010. BEDTools: a flexible suite of utilities for comparing genomic features. *Bioinformatics* **26**: 841–842.

Robinson JT, Thorvaldsdóttir H, Winckler W, Guttman M, Lander ES, Getz G, Mesirov JP. 2011. Integrative genomics viewer. *Nat Biotechnol* **29**: 24–26.

Seal RL, Braschi B, Gray K, Jones TEM, Tweedie S, Haim-Vilmovsky L, Bruford EA. 2023. Genenames.org: the HGNC resources in 2023. *Nucleic Acids Res* **51**: D1003–D1009.

Shumate A, Salzberg SL. 2021. Liftoff: accurate mapping of gene annotations. *Bioinformatics* **37**: 1639–1643.

Smith TF, Waterman MS. 1981. Identification of common molecular subsequences. *J Mol Biol* **147**: 195–197.

Smit AFA, Hubley R, Green P. 2013. *RepeatMasker Open-4.0*. Institute for Systems Biology.

Stovner EB, Sætrom P. 2020. PyRanges: efficient comparison of genomic intervals in Python. *Bioinformatics* **36**: 918–919.

Subramanian A, Tamayo P, Mootha VK, Mukherjee S, Ebert BL, Gillette MA, Paulovich A, Pomeroy SL, Golub TR, Lander ES, et al. 2005. Gene set enrichment analysis: a knowledge-based approach for interpreting genome-wide expression profiles. *Proc Natl Acad Sci USA* **102**: 15545–15550.

Szklarczyk D, Kirsch R, Koutrouli M, Nastou K, Mehryary F, Hachilif R, Gable AL, Fang T, Doncheva NT, Pyysalo S, et al. 2023. The STRING database in 2023: protein-protein association networks and functional enrichment analyses for any sequenced genome of interest. *Nucleic Acids Res* **51**: D638–D646.

Tsunoda T, Takagi T. 1999. Estimating transcription factor bindability on DNA. *Bioinformatics* **15**: 622–630.

Yu Y, Ouyang Y, Yao W. 2018. shinyCircos: an R/Shiny application for interactive creation of Circos plot. *Bioinformatics* **34**: 1229–1231.

Supplemental Methods Table S1: Primers and probes designed and used in this study

Target	T2T CHM13v2.0/hs1[a] location	5′-3′ sequence	Assay	Tm[b] (°C)	Amplicon size[c] (bp)
Chr17p subtelomere	Chr17:5283-5264	GGCTGAACTATAGCCTCTGC	17p intrachromosomal fusion PCR	62	N/A
Chr17p subtelomere	Chr17:2793-2768	GATAGCAAGTTGCCTTACATCACTTG	17p intrachromosomal fusion PCR	62	N/A
ChrXpYp subtelomere	ChrX:3496-3477; ChrY:7328-7309	ACCAGGTTTTCCAGTGTGTT	XpYp intrachromosomal fusion PCR	62	N/A
ChrXpYp subtelomere	ChrX:2689-2671; ChrY:6521-6503	TAGACGGGGACTCCCGAGA	XpYp intrachromosomal fusion PCR	62	N/A
Chr17p subtelomere (17p)	Chr17:3469-3588	CATGCTCTGATGTTTTCTCCATTAGGTGGTCTTGCTAGCACCATCACGGGCAGAGCCAGGTTGTAAATTGACTGTTGTCTGCTGCCAATCTCCTGGTGGGGGAGAGATATGGAAGTGCCA	Chromatin Capture-C	N/A	N/A
Chr17p subtelomere (17p)	Chr17:3797-3916	TGCCAACCAGAAACAGTAGGTTCCCAGGTTACCTTCTGTCTGACTTTGCTACAGATTGGAGGCTCCCACAACACCCTTATTGGGTTCAGCAATTTGCTACATCACATTACACAACCCATG	Chromatin Capture-C	N/A	N/A
ChrXpYp subtelomere (XpT)	ChrX:2433-2552; ChrY:6265-6384	CATGAGCTCCTGGTCTGTAACACAGTTCCCCTGTGGGGATTTAGGGACTTGGGCCTTCTGTCTTTGGGATCTACTCTCTATGGGCCACACAGATATGTCTTCCAACTTCCCTACACAGGG	Chromatin Capture-C	N/A	N/A
ChrXpYp subtelomere (XpT)	ChrX:2803-2922; ChrY:6635-6754	GGACTCGGGCCTCCCCTCTCTAGTGGTCTGGTCATCAGGCCAGGGGCACGTGGAAGAAGCTATCGTGGCAAAGGGAGCAGTCATATCCCCAAAATCTGTGGTTGGTTTACCACCACCATG	Chromatin Capture-C	N/A	N/A
ChrXpYp internal intergenic locus (XpG)	ChrX:726530-726649; ChrY:745899-746018	CATGGGTTTCAAGAACAAATAGATCCATTTAGATAAATAAATCATATCAGTATAATCAAACACCTAGTTTGGGTGATGAGACAGTAGTTTACATAAGAGATGATTGATAGCAGCCTGAAA	Chromatin Capture-C	N/A	N/A
ChrXpYp internal intergenic locus (XpG)	ChrX:726870-726989; ChrY:746239-746358	GCAAGTGGTAGAGGGAGCCCCAGAGGCTCTCTCTCTCCTCATTAAGGAGCTCAAGTGGAAGCAGGAGCAATTTGTCATTGTCTAAAGTGGGAAGACCTTGAAATGAGTCCTGCTTTCATG	Chromatin Capture-C	N/A	N/A
DYNC1H1	Chr14:96228790-96228909	CATGGTATTGTCCTTGATTCCCATTGTGTGTCTCTTGAAAATGAATGCTGAGCTGTTTCACTGGCACCACTCCGTACAAGGTACCATCACCTCTCACTTGGATTATTAATATTCCCTCCT	Chromatin Capture-C	N/A	N/A
DYNC1H1	Chr14:96228920-96229039	TCTGCCTTTGCCTCCCTAAAATCTGTTTTCAACACAGCAGCCAAAGCAAGTTTTAGATTCTAAGTCAATCCTGACACTCTTTTCGGAGCCCTTGAATGACTTCCTATCTCACTCATCATG	Chromatin Capture-C	N/A	N/A
NLRP9	Chr19:58828233-58828352	CATGGCCCAGGCAAATCAAGATAAGTAAAATATCAGGGAACAACTGTGTTTCTATCCTCTCATACATTATTTGTATAAAAGTAATAATAAAAACTTTATTAGGATTAAAAGAAACGGAAG	Chromatin Capture-C	N/A	N/A
NLRP9	Chr19:58828474-58828593	AGCACTCTTCCTTTCTCACCTGAGAAACATCTATTCTCCATCCACTCACATCCAAGCAAAGGTCATCCCTAACCAGGACTGCTCGCTCTCTGGCCCTCCAACTAGATTTCATTTTACATG	Chromatin Capture-C	N/A	N/A

TOP3B	Chr22:22372935-22373054	CATGTGTGGTGTTAATGCACCTGCTCTTTCAACCCAGCCAGGAGGTGTCACTGACCTGGCCTACGGAGGCCTGAAGCCCAGATCTGGGAGCTGGTCCCACGTGGGGACCTGGGTCCCCAG	Chromatin Capture-C	N/A	N/A
TOP3B	Chr22:22373202-22373321	CCTGGGCGATCAGGTTCAGCTGCTTCTCCACTGCACTGCGGATGGTGGGGAGCACCAGCTCTGCATCTGCAAGTGGGCAGGGGGCAGATATTGCCCTGAGCACTCGCACCCAGGGCCATG	Chromatin Capture-C	N/A	N/A
EXT1	Chr8:119232356-119232455	CATGTTCATAAAGTAAAGCAATGGAAAAAAGATCCATATACAAACACCTGACTCACGCTTCACGTATTAAAAGTTAATATGTAACAGGGAGAAACTCTGT	Chromatin Capture-C	N/A	N/A
EXT1	Chr8:119232743-119232842	TTTTAAATAAAAACAATTATAGGTTGAATATCTGTTATCTGAAATGAATGCTAGTTATCCCTTATCTAAAGAAAGTTAATTCCGCACAACCTGAAGCATG	Chromatin Capture-C	N/A	N/A
CCSER1	Chr4:94454040-94454139	CATGTGAATTTTATTGAAGAAAAGCTTAGTCAATTGAGGTAAGAGCAACTATTCTGTCACAAGTTTGGGATGATCAGATATGCTACATTTGTTGTTTACT	Chromatin Capture-C	N/A	N/A
CCSER1	Chr4:94454611-94454710	GTGAAAAAAATTGAGAGATCCTGAAAAATATAATCAGTTCTACTATTCAGGAGATTGTACTTCTACTTCAAAACAGGTTCCTGTAACCGTAGAGACCATG	Chromatin Capture-C	N/A	N/A
RUNX1 eccDNA junction	Chr21:33857273-33857292	TGGAAGCTACGGAGGATTGC	eccDNA junction validation	55	156
RUNX1 eccDNA junction	Chr21:33853899-33853880	CGTAACTAACCTGCACGTCG	eccDNA junction validation	55	156
WWOX eccDNA junction	Chr16:84592253-84592272	TCGGTGGTGAAGTGGGGATA	eccDNA junction validation	55	133
WWOX eccDNA junction	Chr16:84590849-84590830	TGCTGTGGACGTCTGAATCA	eccDNA junction validation	55	133

[a] Human Telomere-to-Telomere reference version CHM13v2.0/hs1

[b] PCR annealing temperature

[c] Amplicon sizes are sample-dependent and measured in base pairs

Supplemental code index

The code used for annotations is in code.zip/annotations.

The code used to align reads and call structural variants is in code.zip/reads2SVs.

The KAS-seq analysis commands are in code.zip/diffbind_commands.R and code.zip/kas-seq_epic2_diffbind_analysis_commands.txt.
