# Supplementary material for: Telomere-driven replicative crisis is driven by large-scale changes in genomic architecture
Source: Genome Res. 2026 Aug;36(8):1529–47. doi: 10.1101/gr.281373.125 (PMC13431167; doi:10.1101/gr.281373.125)
Supplement: Supplement 2 [file Supplemental_code.zip › Supplemental_code/Supplemental_code_index.docx]

**Supplemental code index**

The code used for annotations is in code.zip/annotations.

The code used to align reads and call structural variants is in code.zip/reads2SVs.

The KAS-seq analysis commands are in code.zip/diffbind_commands.R and code.zip/kas-seq_epic2_diffbind_analysis_commands.txt.
